# Determinants of pneumococcal carriage by age in Africa (2000–2021): a systematic analysis

**DOI:** 10.3389/fmed.2025.1683313

**Published:** 2025-12-05

**Authors:** Eliza Mari Kwesi-Maliepaard, Nicholas Kwasi-Do Ohene Opoku, Beverly Egyir, Nicholas TKD Dayie, Kwadwo Asamoah Kusi, Augustina Frimpong

**Affiliations:** 1 Department of Immunology, Noguchi Memorial Institute for Medical Research, College of Health Sciences, University of Ghana, Legon, Ghana; 2 Department of Biochemistry and Biotechnology, Kwame Nkrumah University of Science and Technology, Kumasi, Ghana; 3 Department of Bacteriology, Noguchi Memorial Institute for Medical Research, College of Health Sciences, University of Ghana, Legon, Ghana; 4 Department of Medical Microbiology, University of Ghana Medical School, Accra, Ghana

**Keywords:** pneumococcal carriage, *Streptococcus pneumoniae*, vaccination impact, children, HIV/AIDS, elderly population, Africa

## Abstract

**Background:**

*Streptococcuspneumoniae* (*S. pneumoniae*) is one of the major pathogenic bacteria involved in pneumonia, with pneumococcal pneumonia often preceded by nasopharyngeal carriage of *S. pneumoniae*. The burden of pneumococcal pneumonia is highest in low-and middle-income countries, particularly in Africa. This systematic review examined the determinants of pneumococcal carriage across different age groups in Africa by analysing studies conducted between 2000 and 2021.

**Methods:**

Our search strategy and inclusion criteria focused on studies describing pneumococcal carriage in Africa, from which we extracted data on group size, pneumococcal carriage, identification methods, vaccination status, respiratory tract infections, and underlying diseases.

**Results:**

Our findings indicate the highest pneumococcal carriage rates in children under 5 years, while data on carriage in the elderly (60 years and above) remains limited. HIV/AIDS, as an underlying disease, was associated with increased pneumococcal carriage. Carriage of serotypes included in the pneumococcal vaccines was decreased in those vaccinated compared to non-vaccinated. However, we did not observe an effect of pneumococcal vaccination on total carriage, suggesting replacement of vaccine-type serotypes by non-vaccine serotypes.

**Discussion:**

This indicates the need for further research to understand the impact of pneumococcal vaccination among all age groups in diverse geographical locations. Special emphasis should be given to the elderly population, since they are understudied and have a higher burden of infectious diseases in general. This review provides an overview of pneumococcal carriage in all age groups in Africa, identifying key determinants of carriage and emphasizing the need for targeted interventions to reduce pneumococcal carriage in high-risk populations. Future research should investigate factors contributing to the observed vaccination-carriage relationship and explore strategies to enhance the effectiveness of pneumococcal vaccination programs in Africa.

**Systematic review registration:**

https://www.crd.york.ac.uk/PROSPERO/view/CRD42021274041.

## Introduction

The human nasopharynx hosts a diverse community of bacteria, the majority of which are commensal and contribute to host defense against pathogenic organisms. However, certain species, such as *Streptococcus pneumoniae* (*S. pneumoniae*), can transition from asymptomatic colonization to pathogenic infection, particularly of the lower respiratory tract (1–4). A major virulence determinant of *S. pneumoniae* is its capsule, which consists of a thick layer of polysaccharides and is located on the outermost layer of the cell envelope (3–5). The capsule not only prevents the bacterium from being mechanically removed by mucous secretion, but also modulates adhesion and transmission to epithelial cells, and limits phagocytic uptake of the bacterium (3, 5). Over 100 different capsular types have been identified, with each bacterial serotype exhibiting varying degrees of virulence and immunogenicity (6). Pneumococcal pneumonia remains a leading cause of hospitalization and mortality in infants and the elderly, especially in low-and middle-income countries (7). In children, the risk of infection is largely associated with an underdeveloped immune system, whereas in the elderly, immune senescence plays a critical role in increased risk (8). In 2015, an estimated 257,000 deaths due to pneumococcal pneumonia occurred in children under 5 years of age, and 53% of these deaths were reported in Africa (9). However, since 2000, the global burden of *S. pneumoniae* on childhood mortality has decreased significantly. This reduction is largely attributed to the development and implementation of pneumococcal vaccines in national immunization programmes (7, 9–11). Several different types of pneumococcal vaccines have been developed to date, with the two most common types being pneumococcal polysaccharide capsular vaccines (PPSV) and pneumococcal conjugated vaccines (PCV) (12). PPSVs were developed first, with PPSV23 targeting 23 polysaccharide capsules still being available today. However, PPSVs failed to immunize infants and toddlers (12). The conjugated PCVs can induce an immune response in infants and toddlers and are therefore included in childhood vaccination programmes. The most common types of PCVs target 7, 10 or 13 different serotypes and are named PCV7, PCV10 and PCV13, respectively, (12). To date, most African countries (46/54) have included pneumococcal vaccination in their childhood vaccination programmes (Supplementary Table S1). The two vaccines used on the African continent are PCV10 and PCV13, which cover the pneumococcal serotypes 1, 4, 5, 6B, 7F, 9 V, 14, 18C, 19F and 23F (PCV10) and the additional pneumococcal serotypes 3, 6A and 19A (PCV13) (13). In some high-income countries, PCV is also administered to other vulnerable groups, such as the elderly population, to reduce the risk of developing pneumonia (14, 15).

While pneumococcal conjugate vaccines (PCVs) have significantly reduced the burden of disease caused by vaccine-included serotypes, their implementation has led to the emergence of serotype replacement, a phenomenon in which non-vaccine serotypes (NVTs) increase in prevalence as vaccine-targeted strains decline (16, 17). This shift may be driven by ecological dynamics within the nasopharynx, including direct competition among serotypes and limitations in multi-colonization capacity (18). Although PCVs reduce carriage and disease associated with vaccine serotypes, these gains are often offset by rising NVT carriage and, in some cases, NVT disease, resulting in a net reduction in disease burden that is less than anticipated (19). Moreover, the extent and composition of serotype replacement may vary across geographic regions and age groups (20).

In addition to altering serotype prevalence, the introduction of PCVs has had a significant impact on antimicrobial resistance (AMR). Antibiotics are the first line of treatment for pneumococcal infections like pneumococcal pneumonia (3, 5, 21). As vaccine-targeted serotypes decline, non-vaccine serotypes (NVTs) such as 19A and 6C have emerged as dominant replacement strains in several regions and are frequently associated with multidrug resistance (22, 23). Even though PCVs have successfully reduced resistance among vaccine-included serotypes, this benefit is increasingly offset by rising AMR in NVTs, with studies reporting elevated resistance to penicillin, erythromycin, and other antibiotics in the post-PCV era (24, 25). The net effect has been a stabilization or even increase in overall AMR burden, particularly in settings with high vaccine coverage.

Most studies investigating the nasopharyngeal carriage of *S. pneumoniae* have been limited to studying small age groups, especially those groups where the carriage is expected to be high. In order to understand the dynamics of pneumococcal carriage and identify specific populations that might benefit most from receiving pneumococcal vaccines, it is crucial to analyse data across different age groups. In this paper, we performed a systematic review and meta-analysis to determine pneumococcal carriage by age on the African continent. Furthermore, we identified factors that affect pneumococcal carriage in the African population. The findings of this study will contribute to a better understanding of pneumococcal carriage dynamics in Africa and may help guide targeted interventions to reduce pneumococcal carriage and related disease burden in high-risk populations.

## Methods

### Paper selection

**Figure 1:**
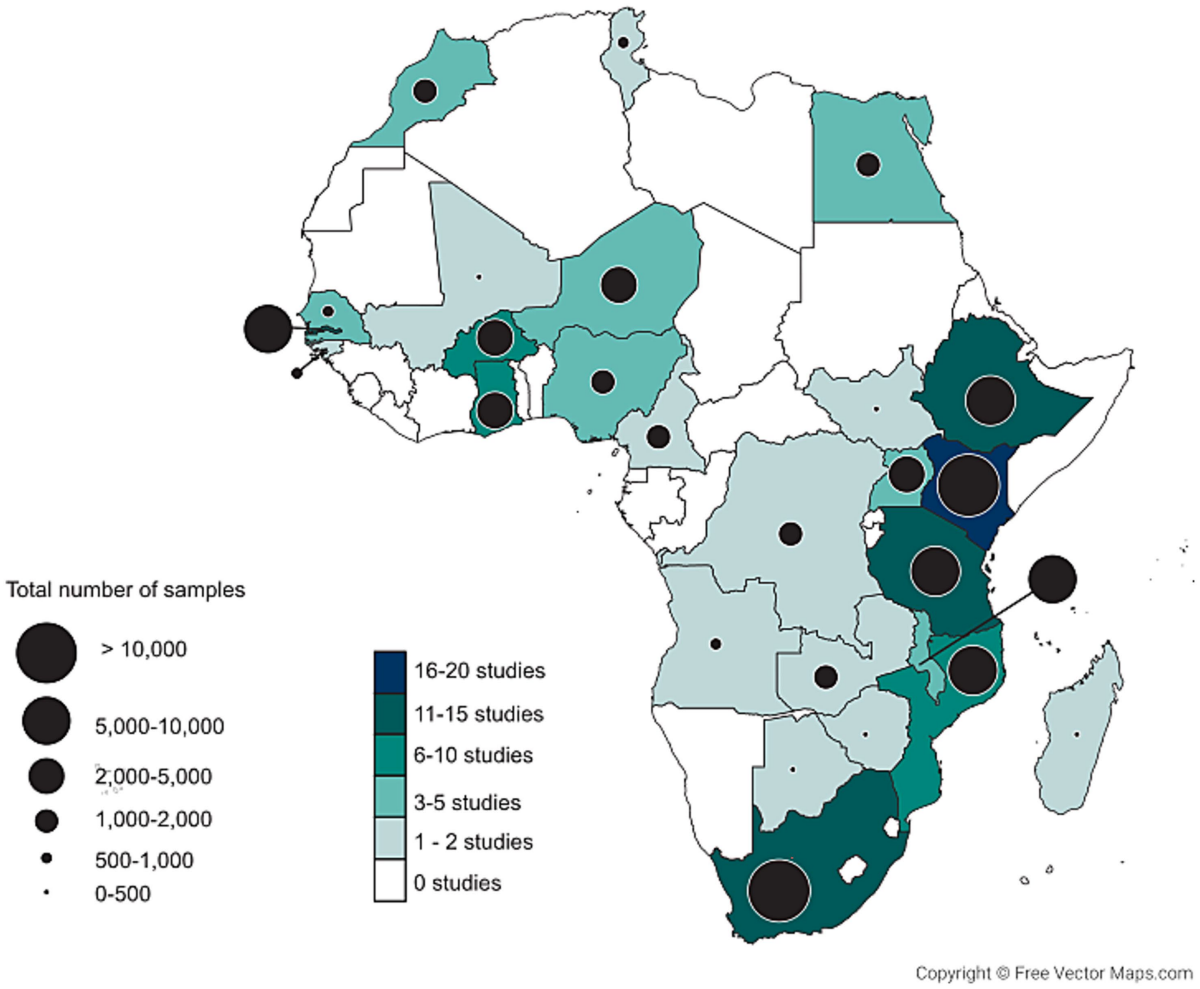
Geographical distribution of the studies and samples included in this systematic review.

This systematic review is registered at PROSPERO (https://www.crd.york.ac.uk/PROSPERO/view/CRD42021274041). We searched three databases, PubMed, Medline and Africa Index Medicus for papers containing pneumococcal carriage data. The following search terms were used: *Streptococcus pneumoniae* + Africa, *Streptococcus pneumoniae* + [name African country], pneumococcal disease + Africa, pneumococcal disease + [name African country]. Papers published between January 2000 and September 2021 were included. This search resulted in a total of 4,410 papers. Out of these there were 1,361 duplicates, after removal of the duplicates 3,049 unique papers were assessed. Firstly, titles and abstracts were scanned for eligibility. Only studies written in English that described pneumococcal carriage in humans in Africa were included. Case reports, non-peer-reviewed pieces like opinions, preprints, posters, editorials and studies with samples collected before 2000 were discarded. Furthermore, inclusion criteria were restricted to studies reporting pneumococcal carriage in the upper respiratory tract and not in other body sites. We only included one data point per participant, therefore longitudinal studies and randomized controlled trials were only included when there was baseline data available. Afterward, full-text PDFs were obtained and analysed according to the criteria described above. When the full text was not available from the publishers’ websites, we tried to reach out to the authors on ResearchGate. The quality of the articles was assessed based on the guidelines described in the Joanna Briggs critical appraisal tools (26). No articles were excluded based on the critical appraisal. For the analyses, only papers stating both the numerator and denominator of the carriage prevalence were included. Also, due to variation of carriage between age groups, we set the following criteria for the age groups to be included; in children, the maximum age bracket is 10 years, studies that only described age categories as children or adults, without any median/average age or age range were excluded, data from children and adults in the same group were also excluded. For adults, no limits were set for the age brackets. When age brackets were available the midpoint age was used, when there were no age brackets the average or median age as reported in the paper was used. When the age group was only marked as >15 years we set 60 years as the upper limit, when the age groups was marked as >49, >50 or >60 years we set 75 years as the upper limit. When information about the age range, median/average or numerator and denominator was missing we tried to reach out to the authors by email to obtain this information. Finally, 134 papers were included for meta-analysis containing data from a total of 98,036 swabs (Supplementary Figure S1). A detailed overview of the paper selection process is provided in Figure 1.

### Data analysis

The statistical analysis which incorporates the regression model and generation of graphs was done using R (v.4.2.2). In order to model carriage by age we used a similar approach as Christensen et al. (27). The carriage by age was non-linear, as a result, the mixed-effects logistic regression model with natural spline was used to model carriage prevalence as a function of age. The RC_SPLINE function was used to provide plots of the estimated restricted cubic spline function relating to a single predictor to the response for the logistic model. Initially, we placed the knots based on the locations of percentiles. However, this did not give a good fit. We, therefore, added two additional knots at 1 and 5 years, resulting in a good fit. The location of the additional knots was chosen based on the fact that many studies were limited to studying either babies <1 year of age or children <5 years of age. To further improve the predicted pneumococcal carriage by age we added a mixed-effect logistic regression model to the natural spline. The model included the reference and country as random effects and the era of sampling, pneumococcal identification method, underlying disease, vaccination status, and lung infection status as fixed effects. We used the Akaike’s information criterion (AIC) to select the best fitting model (a lower value suggests a better model fit) (28, 29).

Serotyping data was extracted from 66 of the 134 papers included for carriage prediction. The number of PCV serotypes and then the number of total isolates serotypes was extracted and the percentage of PCV serotypes was calculated. Vaccination status was determined as ‘no’ when <25% of the participants were vaccinated, ‘mix’ when 25–74% of the participants was vaccinated, and ‘yes’ when ≥75% of the participants was vaccinated. Differences in the percentage of PCV serotypes between the three vaccination statuses were calculated using multiple comparisons of ordinary one-way ANOVA in GraphPad Prism (v 9.0.0.).

Antimicrobial sensitivity data was extracted from 48 of the 134 papers included for carriage prediction. The percentage of resistant, intermediate and sensitive bacterial strains for any antimicrobial reported was extracted from the text or calculated based on the absolute numbers. We reported levels of non-sensitivity. Non-sensitivity was calculated by combining resistant and intermediate sensitivity percentages or by subtracting the percentage of sensitive strains. Differences in non-sensitivity for selected antibiotics over time were calculated using multiple comparisons of ordinary one-way ANOVA in GraphPad Prism (v 9.0.0).

## Results

### Characteristics of the study

We identified 134 papers describing pneumococcal carriage in Africa (Supplementary Figure S1; Supplementary Table S2). Study sizes ranged from two to 2,405 persons per age group. Pneumococci were mostly identified and serotyped by a variety of methods (Supplementary Table S2 and S3). Samples were collected in 26 of the 54 African countries, with most of the data coming from a few countries in East Africa, South Africa and The Gambia (Figure 1). Samples from the East African countries Kenya, Mozambique, Tanzania, Ethiopia and Malawi accounted for 45.9% of all samples analysed in this review. Samples from South Africa and The Gambia accounted for 17.7 and 8.4%, respectively.

### A predictive model of pneumococcal carriage by age

**Figure 2:**
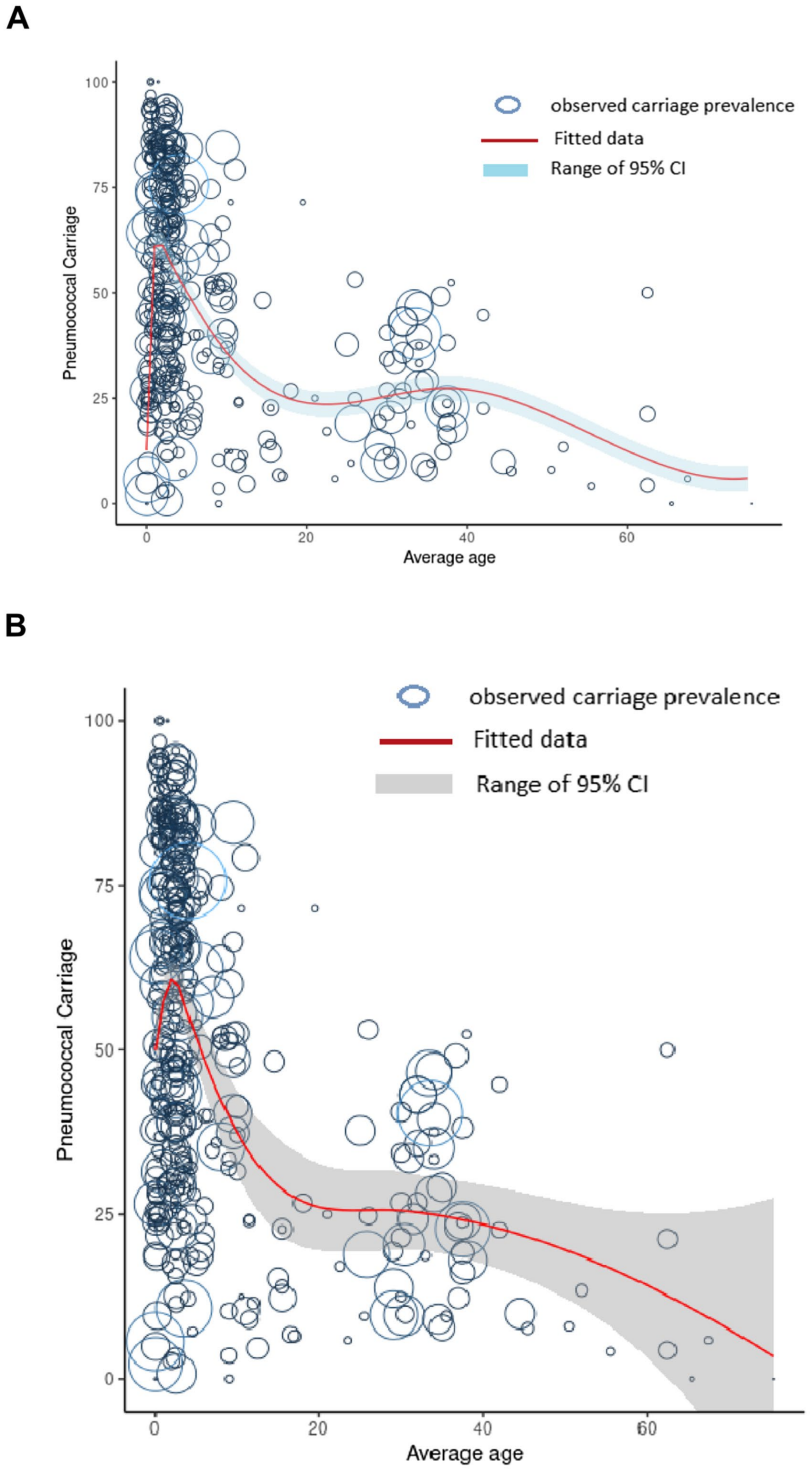
Prediction of pneumococcal carriage by age. **(A)** Prediction of pneumococcal carriage by age based on a natural spline only. **(B)** Prediction of pneumococcal carriage by age based on a mixed-effect logistic regression model with a natural spline. The size of the circle corresponds with the group size.

This first model to predict pneumococcal carriage by age, based on a simple natural spline, showed a peak of pneumococcal carriage in children <5, and a small second bump between 30 and 40 years (Figure 2A). In the final model, which included several additional parameters, the peak of the carriage was still observed in children under five (Figure 2B). After this age, carriage decreased dramatically to around 25% in adults aged 20–40 years and decreased further in older adults (Figure 2B). Of note, the 95% confidence interval was most narrow at young age groups and widest above the average age of 60 years.

### Determinants of pneumococcal carriage

The variance for the estimate for the country random-effect parameter was lower than the variance for the reference random-effect parameter (Table 1). This indicated that there was greater variation between studies than between countries.

**Table 1:** Random and fixed effects estimates.

Random effects		
Reference	Variance 410.7	95% CI 17.17–23.69
Country	Variance 195.4	95% CI 9.71–19.70
Fixed effects		
Era of sampling		
2000–2005	OR 1.0	
2006–2010	OR 12.19	95% CI 0.002–9.54e+04
2011–2015	OR 3.00	95% CI 0.005–1.70e+03
2016–2019	OR 2.76	95% CI 0.001–1.28e+04
Pneumococcal identification method		
Culture	OR 1.0	
PCR	OR 1.31	95% CI 0.20–8.77
FTD	OR 1.24	95% CI 0.01–278.93
Nd	OR 0.85	95% CI 0.07–10.88
PCV vaccination status		
No (<25% vaccinated)	OR 1.0	
Mix (≥25–74% vaccinated)	OR 0.98	95% CI 0.88–1.09
Yes (≥75% vaccinated)	OR 1.15	95% CI 0.98–1.20
Nd	OR 1.48	95% CI 1.10–1.53
Underlying disease		
No underlying disease	OR 1.0	
HIV	OR 2.33	95% CI 2.57–3.98
Mix (HIV and no HIV)	OR 0.27	95% CI 0.003–21.13
Sickle cell	OR 0.23	95% CI 0.00–2751.13
Nd	OR 0.29	95% CI 0.01–6.02
Respiratory tract infection		
No	OR 1.0	
Yes	OR 1.19	95% CI 1.03–1.37
Mix	OR 1.21	95% CI 1.06–1.39
Nd	OR 1.33	95% CI 1.18–1.49

According to our analysis, HIV as an underlying disease emerged as an important determinant of pneumococcal carriage (OR 2.33) (Table 1). Furthermore, slightly higher pneumococcal carriage was associated with groups reporting respiratory tract infection (Table 1).

There was no statistically significant impact of the era of sampling or pneumococcal identification method on pneumococcal carriage (Table 1). It should be noted that the confidence interval for these variables was very wide, and therefore be interpreted with caution. In our analysis, we did not observe an effect of the PCV vaccination status on pneumococcal carriage, except for a higher carriage in studies that did not report the vaccination status (Table 1).

### Serotype analyses based on vaccination status

**Figure 3:**
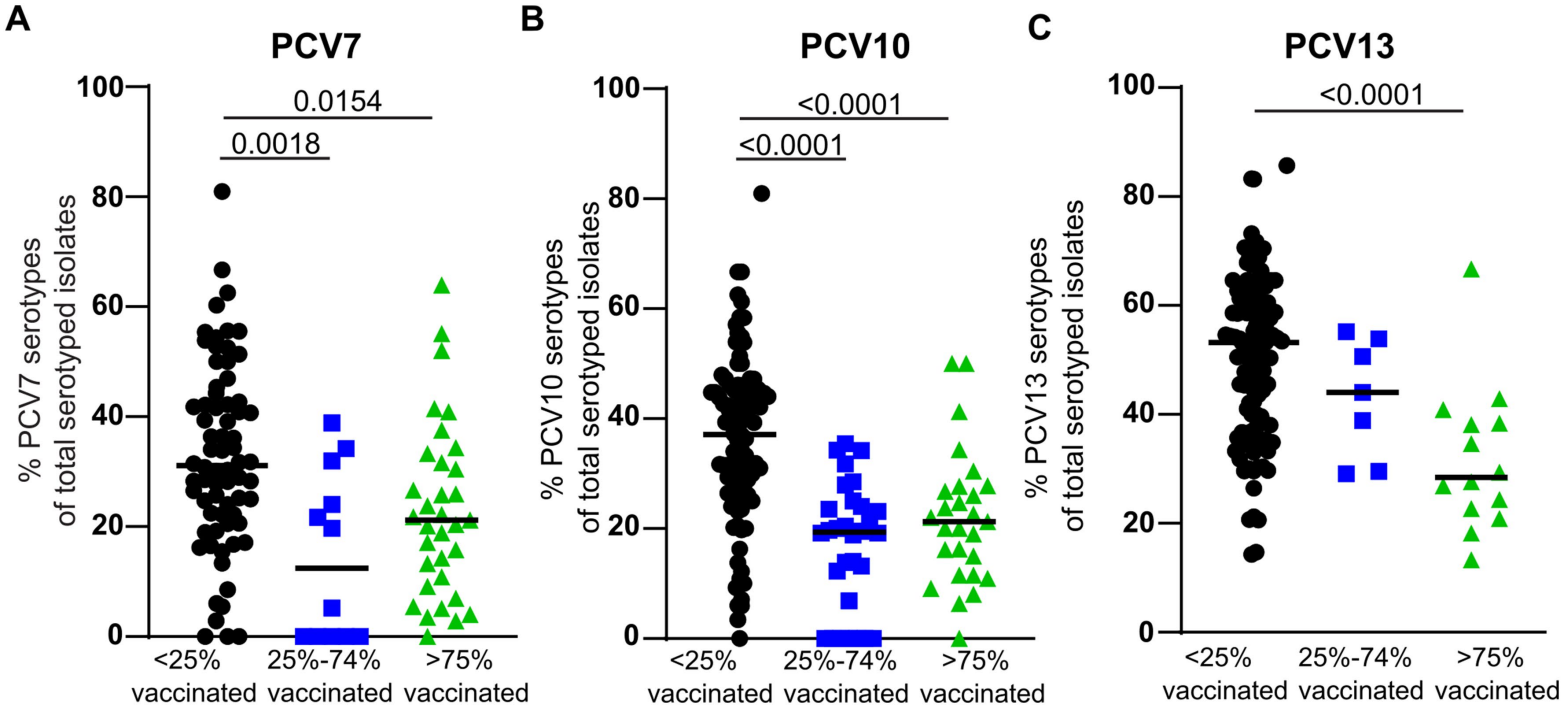
Serotype analyses. **(A)** The percentage of PCV7 serotypes of total serotyped isolates according to vaccination status. The categories ‘25–74% vaccinated’ and ‘>75% vaccinated’ contained those vaccinated with PCV7, PCV10 or PCV13. Number of datapoints for <25% vaccinated (*n* = 70), for 25–74% vaccinated (*n* = 12), and for >75% vaccinated (*n* = 33). **(B)** The percentage of PCV10 serotypes of total serotyped isolates according to vaccination status. The categories ‘25–74% vaccinated’ and ‘>75% vaccinated’ contained those vaccinated with PCV10 or PCV13. Number of datapoints for <25% vaccinated (*n* = 98), for 25–74% vaccinated (*n* = 30), and for >75% vaccinated (*n* = 27). **(C)** The percentage of PCV13 serotypes of total serotyped isolates according to vaccination status. The categories ‘25–74% vaccinated’ and ‘>75% vaccinated’ contained those vaccinated with PCV13. Number of datapoints for <25% vaccinated (*n* = 111), for 25–74% vaccinated (*n* = 7), and for >75% vaccinated (*n* = 14).

Serotyping data was available for 66 of the papers included in this study (Supplementary Table S3). Vaccination status was categorized based on the PCV vaccination grade as ‘no’ (<25% vaccinated), ‘mix’ (25–74% vaccinated), or ‘yes’ (≥75% vaccinated). For PCV7 and PCV10 the percentage of PCV-serotypes was lower in ‘mix’ and ‘yes’ compared to the ‘no’ vaccination status (Figures 3A,B). For PCV13 the percentage of PCV13 serotypes was lower in ‘yes’ compared to the ‘no’ vaccination status (Figure 3C). This indicates that although total pneumococcal carriage was not reduced upon pneumococcal vaccination, the PCV serotypes were replaced by non-vaccine types.

### Antimicrobial sensitivity

**Figure 4:**
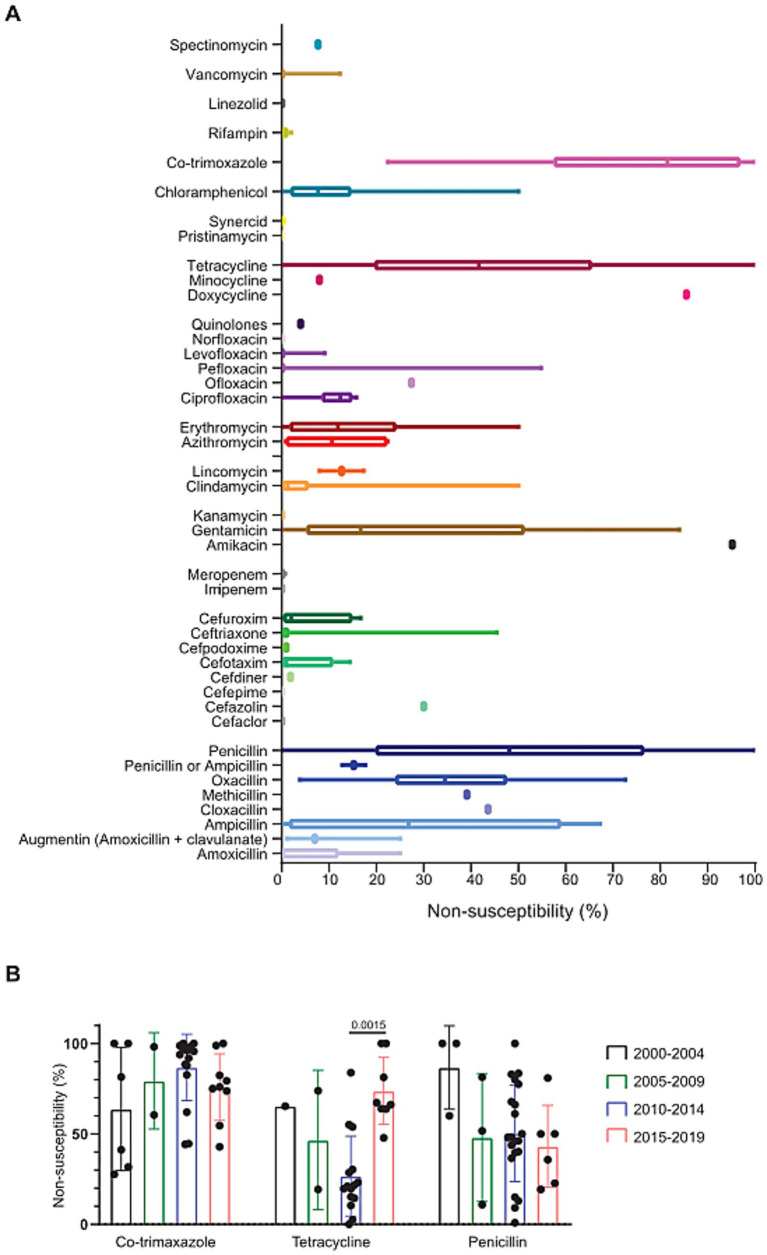
**(A)** The percentage of isolates that were non-susceptible to the indicated antimicrobial reagents. Whiskers indicate minimum to maximum value. Amoxicillin *n* = 9, Augmentin *n* = 3, Ampicillin *n* = 9, Cloxacillin *n* = 1, Methicillin *n* = 1, Oxacillin *n* = 9, Penicillin or Ampicillin *n* = 2, Penicillin *n* = 48, Cefaclor *n* = 1, Cefazolin *n* = 1, Cefepime *n* = 1, Cefdiner *n* = 1, Cefotaxime *n* = 5, Cefpodoxime *n* = 1, Ceftriaxone *n* = 23, Cefuroxim *n* = 9, Imipenem *n* = 3, Meropenem *n* = 4, Amikacin *n* = 1, Gentamicin *n* = 5, Kanamycin *n* = 1, Clindamycin *n* = 28, Lincomycin *n* = 2, Azithromycin *n* = 6, Erythromycin *n* = 46, Ciprofloxacin *n* = 6, Ofloxacin *n* = 1, Pefloxacin *n* = 3, Levofloxacin *n* = 16, Norfloxacin *n* = 3, Quinolones *n* = 1, Doxycycline *n* = 1, Minocycline *n* = 1, Tetracycline *n* = 38, Pristinamycin *n* = 1, Synercid *n* = 3, Chloramphenicol *n* = 38, Co-trimaxazole *n* = 49, Rifampin *n* = 9, Linezolid *n* = 4, Vancomycin *n* = 14, Spectinomycin *n* = 1. **(B)** Non-susceptibility to co-trimoxazole (2000–2004 *n* = 6, 2005–2009 *n* = 2, 2010–214 *n* = 17, 2025–2019 *n* = 9), tetracycline (2000–2004 *n* = 1, 2005–2009 *n* = 2, 2010–2014 *n* = 15,2015–2019 *n* = 8) and penicillin (2000–2004 *n* = 3, 2005–2009 *n* = 3, 2010–2014 *n* = 21, 2015–2019 *n* = 6) in four different time points.

Out of the 131 papers included in this study, 48 contained antimicrobial sensitivity data (Supplementary Table S4). Although non-susceptibility levels showed great variability between studies, there were clear trends in sensitivity between different antimicrobials. Notably, high levels of non-susceptibility were observed for co-trimoxazole (75.5%, SD = 24.1), the penicillin class of antibiotics (38.4%, SD = 29.6), and tetracyclines (43.6%, SD = 28.3) (Figure 4A). When assessing this over time, we did not observe a clear trend in antimicrobial sensitivity changes over time, although there was an increase in non-susceptibility for tetracycline between the periods of 2010–2014 and 2015–2019 (Figure 4B). Taken together, although there is variation between the studies, there is a general trend of high non-susceptibility for commonly used antibiotics in *S. pneumoniae*.

## Discussion

In this systematic review, we performed a meta-analysis to assess determinants of pneumococcal carriage on the African continent. Most studies assessing pneumococcal carriage have been limited to studying small age groups, mainly children under the age of 5 years old. Here, we combined all studies reporting pneumococcal carriage data from 2000 to 2021 and assessed pneumococcal carriage by age. Furthermore, we investigated how other factors, including underlying disease, respiratory tract infection, PCV vaccination status and year of sampling, affect pneumococcal carriage.

Earlier systematic reviews have been published reporting pneumococcal carriage in children <5 years (11, 30) or in three age brackets (<5 years, 5–15 years, > 15 years) (31). To our knowledge, this is the first systematic review to predict carriage for all ages, rather than categorizing them into big age brackets. Furthermore, we investigated multiple factors that may also affect pneumococcal carriage.

In our model, the peak of pneumococcal carriage is observed in children <5 years old. This is in accordance with the literature on pneumococcal carriage globally (3, 4). As a result, most data are available for the <5 years age group. Data about pneumococcal carriage in the elderly is particularly scarce. We identified only six groups from four studies with an average age above 60 years (32–35), from which only one study reported on the very old (71–80 years) (32).

The global prevalence of *S. pneumoniae* carriage in healthy aged adults has been estimated to be around 20 and 50% in those with influenza (36–40). Given the growing elderly population in Africa and their specific health issues, including an increased burden of pneumonia, more research into pneumococcal carriage in the elderly is required (8, 41). This would also aid in making informed decisions for the introduction of pneumococcal vaccination in the elderly in Africa, as is already the case in many high-income countries (14, 15).

In the first model, before applying the random and fixed effect parameters, a smaller second knot (bump) in pneumococcal carriage is observed around the 30–40 years age group. Since we used the average or median age as reported in the papers, most studies describing pneumococcal carriage in adults had an average or medium age between 30 and 40 years. Many of these studies contained data from HIV-positive individuals. Based on our analysis, the small bump around the 30–40 years age group reflects the HIV status of the participants. A similar trend of increased nasal carriage in HIV patients compared to the non-HIV participants has also been reported for *Staphylococcus aureus* (42–44).

Interestingly, based on the studies reviewed, we observed that pneumococcal vaccination status did not affect total pneumococcal carriage. This might initially sound surprising given the well-documented effect of PCV on pneumococcal infection (12). However, this might be accounted for by serotype replacement (16). The PCVs used in childhood vaccination programs only target up to 13 pneumococcal serotypes out of the over 100 serotypes present (Supplementary Table S1) (12). Upon pneumococcal vaccination, these PCV serotypes are diminished and other serotypes take over their place in the nasopharynx (16). In our analyses, we also observe this replacement of PCV serotypes by non-vaccine serotypes resulting in the total pneumococcal carriage being not affected. It should further be noted that most African countries only started PCV vaccination after 2010 and that this is often only given to young infants.

Antimicrobial sensitivity of *S. pneumoniae* was determined in around 37% of the studies that reported pneumococcal carriage. The level of non-susceptibility varied between studies, but certain antibiotics showed a high average level of non-susceptibility. Antibiotics that were often reported as having high AMR levels were co-trimoxazole, penicillin and tetracycline. Co-trimoxazole is often given as a prophylactic drug to prevent opportunistic infections in HIV/AIDS patients (45). Importantly, continuous use of co-trimoxazole has been linked to increased antimicrobial resistance in *S. pneumoniae* and other pathogenic bacteria (46, 47). Furthermore, prophylactic use of co-trimoxazole has also been shown to induce increased antibiotic resistance gene diversity and prevalence in stool samples from HIV-exposed uninfected infants (48). Penicillin and tetracycline are both commonly used in the treatment of community-acquired pneumonia (21, 49). Penicillin resistance in *S. pneumoniae* is a worldwide problem (50). The main risk factor for infection with penicillin-resistant *S. pneumoniae* is the use of *β*-lactam antibiotics by the patient in the previous 3–6 months (50). Tetracycline resistance in *S. pneumoniae* is often caused by a mutation in the *tetM* gene, resulting in the removal of Tetracycline from its binding site (50). High prevalence of antibiotic use in hospitals and high rates of dispensing antibiotics without prescriptions have been reported in Africa and are linked to increasing AMR rate (51, 52). A limiting factor in the interpretation of the AMR data is that different laboratory methods were used in AST. AMR readiness varies between laboratories on the continent and has been linked to differences in reported AMR (43, 44). Taken together, our analysis supports the link between antibiotic use and resistance rates and indicates the urgency for improved guidelines for antibiotic treatment and stewardship and the development of new antibiotics.

A limitation of this study is that data was not equally available from all countries. Over 70% of all samples came from studies performed in 7 African countries, whereas 28 out of the 54 African countries did not have any study available that met our inclusion criteria. Additionally, there was also heterogeneity between studies resulting in very wide confidence intervals for the Odds ratios associated with some variables. This makes it difficult to draw conclusions for the whole continent. It also reflects the disparity in the research capacity on the continent (53, 54). Nonetheless, having a good monitoring system for pneumococcal carriage and antimicrobial resistance is essential to improve treatment guidelines and lift the burden of pneumococcal infection on society (55).

## Recommendations

Based on the data currently available we would recommend strengthening surveillance *of S. pneumoniae* carriage on the African continent. Special attention should be paid to those parts of the continent that are underrepresented in the current surveillance data. More data is also needed on specific subgroups, like the elderly. Given the growing aging population in Africa there is an urgent need for studies investigating pneumococcal carriage in African elderly. This is especially relevant considering the unique physical and medical needs of the elderly, and their higher susceptibility to infectious diseases in general. Furthermore, it is recommended to specifically monitor carriage in people with underlying diseases, especially immunocompromised patients who often receive prophylactic antibiotic treatment. Finally, to support vaccine development and diagnostics we need to better understand the biology of *S. pneumoniae* and the pathogenesis of pneumonia. To this end, a thorough pneumococcal surveillance program is required consisting of phenotypic and genomic tools that will help to better study the organisms themselves.

Future research could also further investigate the effect of PCV on pneumococcal carriage in more detail, including potential differences in vaccine effectiveness across countries or in different subgroups. Another interesting question raised by our analysis is to investigate which factors contribute to the observed variation in antimicrobial resistance.

## Data Availability

The original contributions presented in the study are included in the article/Supplementary material, further inquiries can be directed to the corresponding author.
