# Supplementary material for: Determinants of pneumococcal carriage by age in Africa (2000–2021): a systematic analysis
Source: Front Med (Lausanne). 2025 Dec 5;12:1683313. doi: 10.3389/fmed.2025.1683313 (PMC12715941; doi:10.3389/fmed.2025.1683313)
Supplement: Supplementary file 1 [file Data_Sheet_1.docx]

Supplementary data for

**Determinants of pneumococcal carriage by age in Africa**

**A systematic review (2000-2021)**

Eliza Mari Kwesi-Maliepaard^1^, Nicholas Kwasi-Do Ohene Opoku^2^, Beverly Egyir^3^, Nicholas KTD Dayie^4^, Kwadwo Asamoah Kusi^1^, Augustina Frimpong^1*^

*1 Department of Immunology, Noguchi Memorial Institute for Medical Research, College of Health Sciences, University of Ghana, Legon, Ghana*

*2 Department of Biochemistry and Biotechnology, Kwame Nkrumah University of Science and Technology*

*3 Department of Bacteriology, Noguchi Memorial Institute for Medical Research, College of Health Sciences, University of Ghana, Legon, Ghana*

*4 Department of Medical Microbiology, University of Ghana Medical School, Korle Bu, Accra, Ghana.*

Sup figure S1: Article selection

Sup table S1: PCV in childhood vaccination programmes in Africa…………………………..3

Sup table S2: Selected papers for data extraction……………………………………………………4

Sup table S3: Serotyping data……………………………………………………………………………….23

Sup table S4: Antimicrobial sensitivity data…………………………………………………………..33


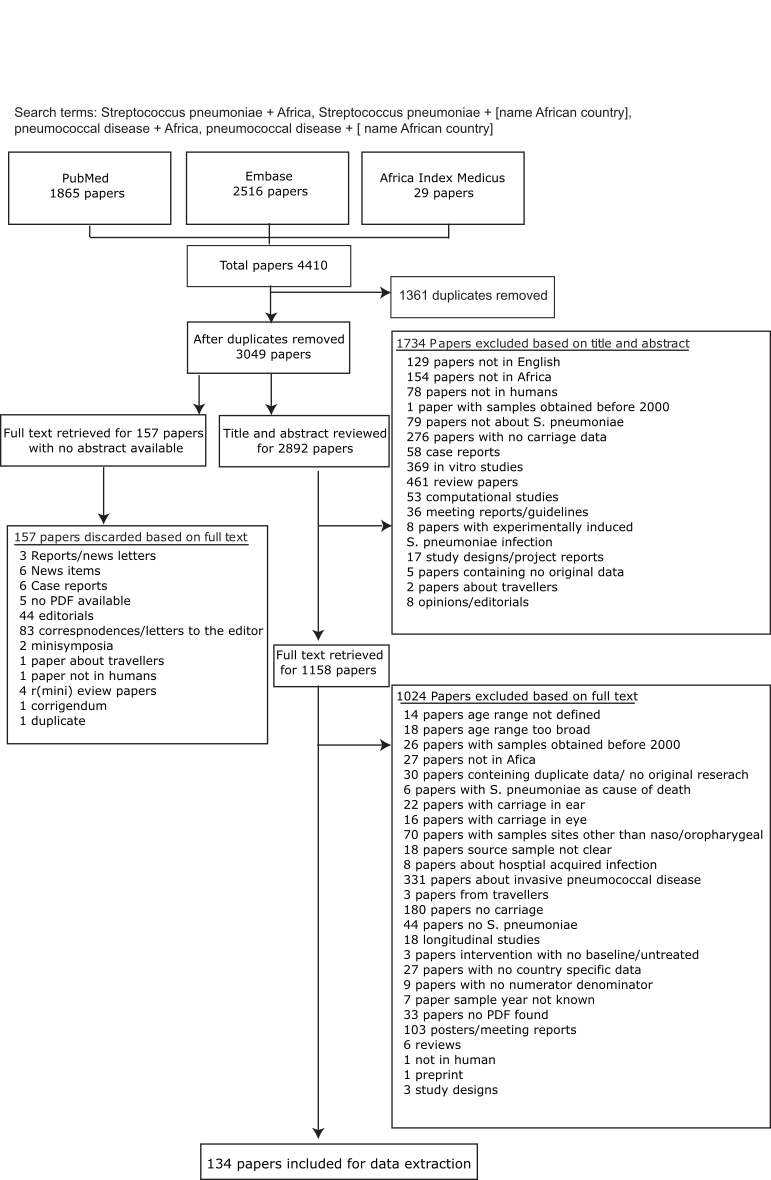
Sup fig S1: Article selection

Sup table S1: PCV in childhood vaccination programmes in Africa

| **Country** | **Region** | **PCV vaccination strategy** | **Start year** |
| --- | --- | --- | --- |
| [Djibouti](https://www.worldometers.info/world-population/djibouti-population/) | Eastern Africa | PCV13 | 2012 |
| [Seychelles](https://www.worldometers.info/world-population/seychelles-population/) | Eastern Africa | PCV13 | 2018 |
| [Comoros](https://www.worldometers.info/world-population/comoros-population/) | Eastern Africa | - | - |
| [Tanzania](https://www.worldometers.info/world-population/tanzania-population/) | Eastern Africa | PCV13 | 2013 |
| [Kenya](https://www.worldometers.info/world-population/kenya-population/) | Eastern Africa | PCV10 | 2011 |
| [Uganda](https://www.worldometers.info/world-population/uganda-population/) | Eastern Africa | PCV10 | 2014 (Partially started in 2013) |
| [Eritrea](https://www.worldometers.info/world-population/eritrea-population/) | Eastern Africa | PCV13 | 2015 |
| [Mozambique](https://www.worldometers.info/world-population/mozambique-population/) | Eastern Africa | PCV13 | 2013 |
| [Madagascar](https://www.worldometers.info/world-population/madagascar-population/) | Eastern Africa | PCV10 | 2012 |
| [Malawi](https://www.worldometers.info/world-population/malawi-population/) | Eastern Africa | PCV13 | 2011 |
| [Zambia](https://www.worldometers.info/world-population/zambia-population/) | Eastern Africa | PCV10 | 2013 |
| [Somalia](https://www.worldometers.info/world-population/somalia-population/) | Eastern Africa |  | - |
| [Zimbabwe](https://www.worldometers.info/world-population/zimbabwe-population/) | Eastern Africa | PCV13 | 2012 |
| [Rwanda](https://www.worldometers.info/world-population/rwanda-population/) | Eastern Africa | PCV13 | 2009 |
| [Mauritius](https://www.worldometers.info/world-population/mauritius-population/) | Eastern Africa | PCV13 | 2016 |
| [Burundi](https://www.worldometers.info/world-population/burundi-population/) | Eastern Africa | PCV13 | 2011 |
| [Ethiopia](https://www.worldometers.info/world-population/ethiopia-population/) | Eastern Africa | PCV10 | 2011 |
| [South Sudan](https://www.worldometers.info/world-population/south-sudan-population/) | Eastern Africa |  | - |
| [DR Congo](https://www.worldometers.info/world-population/democratic-republic-of-the-congo-population/) | Middle Africa | PCV13 | 2013 (Partially started in 2011) |
| [Congo](https://www.worldometers.info/world-population/congo-population/) | Middle Africa | PCV13 | 2012 |
| [Central African Republic](https://www.worldometers.info/world-population/central-african-republic-population/) | Middle Africa | PCV13 | 2011 |
| [Angola](https://www.worldometers.info/world-population/angola-population/) | Middle Africa | PCV13 | 2013 |
| [Cameroon](https://www.worldometers.info/world-population/cameroon-population/) | Middle Africa | PCV13 | 2011 |
| [Gabon](https://www.worldometers.info/world-population/gabon-population/) | Middle Africa |  | - |
| [Sao Tome & Principe](https://www.worldometers.info/world-population/sao-tome-and-principe-population/) | Middle Africa | PCV13 | 2012 |
| [Chad](https://www.worldometers.info/world-population/chad-population/) | Middle Africa | PCV13 | 2024 |
| [Equatorial Guinea](https://www.worldometers.info/world-population/equatorial-guinea-population/) | Middle Africa | - | - |
| [Libya](https://www.worldometers.info/world-population/libya-population/) | Northern Africa | PCV13 | 2013 |
| [Algeria](https://www.worldometers.info/world-population/algeria-population/) | Northern Africa | PCV13 | 2016 |
| [Sudan](https://www.worldometers.info/world-population/sudan-population/) | Northern Africa | PCV13 | 2013 |
| [Morocco](https://www.worldometers.info/world-population/morocco-population/) | Northern Africa | PCV10 | 2010 |
| [Tunisia](https://www.worldometers.info/world-population/tunisia-population/) | Northern Africa | PCV10 | 2019 |
| [Egypt](https://www.worldometers.info/world-population/egypt-population/) | Northern Africa |  | - |
| [South Africa](https://www.worldometers.info/world-population/south-africa-population/) | Southern Africa | PCV13 since 2011  PCV7 since 2009 | 2009 (Partially started in 2008) |
| [Namibia](https://www.worldometers.info/world-population/namibia-population/) | Southern Africa | PCV10 | 2014 |
| [Botswana](https://www.worldometers.info/world-population/botswana-population/) | Southern Africa | PCV13 | 2012 |
| [Lesotho](https://www.worldometers.info/world-population/lesotho-population/) | Southern Africa | PCV13 | 2015 |
| [Eswatini](https://www.worldometers.info/world-population/swaziland-population/) | Southern Africa | PCV13 | 2014 |
| [Togo](https://www.worldometers.info/world-population/togo-population/) | Western Africa | PCV13 | 2014 |
| [Sierra Leone](https://www.worldometers.info/world-population/sierra-leone-population/) | Western Africa | PCV13 | 2011 |
| [Cabo Verde](https://www.worldometers.info/world-population/cabo-verde-population/) | Western Africa |  | - |
| [Liberia](https://www.worldometers.info/world-population/liberia-population/) | Western Africa | PCV13 | 2014 |
| [Mauritania](https://www.worldometers.info/world-population/mauritania-population/) | Western Africa | PCV13 | 2013 |
| [Ghana](https://www.worldometers.info/world-population/ghana-population/) | Western Africa | PCV13 | 2012 |
| [Côte d'Ivoire](https://www.worldometers.info/world-population/cote-d-ivoire-population/) | Western Africa | PCV13 | 2014 |
| [Niger](https://www.worldometers.info/world-population/niger-population/) | Western Africa | PCV13 | 2014 |
| [Gambia](https://www.worldometers.info/world-population/gambia-population/) | Western Africa | PCV13 | 2009 |
| [Burkina Faso](https://www.worldometers.info/world-population/burkina-faso-population/) | Western Africa | PCV13 | 2013 |
| [Nigeria](https://www.worldometers.info/world-population/nigeria-population/) | Western Africa | PCV10 | 2017 (Partially started in 2015) |
| [Mali](https://www.worldometers.info/world-population/mali-population/) | Western Africa | PCV13 | 2011 |
| [Guinea-Bissau](https://www.worldometers.info/world-population/guinea-bissau-population/) | Western Africa | PCV13 | 2015 |
| [Senegal](https://www.worldometers.info/world-population/senegal-population/) | Western Africa | PCV13 | 2013 |
| [Guinea](https://www.worldometers.info/world-population/guinea-population/) | Western Africa | - | - |
| [Benin](https://www.worldometers.info/world-population/benin-population/) | Western Africa | PCV13 | 2011 |

Data obtained from the WHO website ([www.who.int](http://www.who.int)) visited on 26^th^ September 2025.

| Sup Table S2: Selected papers for data extraction | | | | | | | | | | | | | | | | | | |  |
| --- | --- | --- | --- | --- | --- | --- | --- | --- | --- | --- | --- | --- | --- | --- | --- | --- | --- | --- | --- |
| Reference | Country | WHO  region | sample_type | year_sampling | era_sampling | | identification | Age_group | Age_low | Age_high | Age_midpoint | numerator | denominator | percentage | Lung_infection | Underlying_disease | Vaccination_status | Vaccine | |
| Warda 2013(1) | Morocco | North | Nasopharyngeal | 2007-2008 | 2 | Culture | | 1-24 m | 0 | 2 | 1 | 302 | 660 | 45.76 | nd | nd | no |  | |
| Jroundi 2017(2) | Morocco | North | Nasopharyngeal | 2010-2011 | 3 | Culture | | 2-59 m | 0.17 | 4.92 | 2.545 | 159 | 697 | 22.81 | yes | nd | no |  | |
|  |  |  |  |  |  |  | | 2-59 m | 0.17 | 4.92 | 2.545 | 79 | 195 | 40.51 | no | nd | mix | PCV13 | |
| Dilagui 2019(3) | Morocco | North | Nasopharyngeal | 2017 | 4 | Culture | | 2-18 m | 0.17 | 1.5 | 0.835 | 125 | 183 | 68.31 | yes | nd | mix | PCV10 | |
| Brini 2017 (4) | Tunisia | North | Nasopharyngeal aspirate | 2013-2014 | 3 | PCR | | 0-5 y | 0 | 5 | 2.5 | 143 | 372 | 38.44 | yes | nd | no |  | |
| Brini Khalifa 2018 (5) | Tunisia | North | Nasopharyngeal | 2013-2014 | 3 | PCR | | 0-28 d | 0 | 0.08 | 0.04 | 40 | 211 | 18.96 | yes | nd | no |  | |
|  |  |  |  |  |  |  | | 28d-6m | 0.08 | 0.5 | 0.29 | 78 | 206 | 37.86 | yes | nd | no |  | |
|  |  |  |  |  |  |  | | 6m-12m | 0.5 | 1 | 0.75 | 41 | 98 | 41.84 | yes | nd | no |  | |
| Saafan 2013 (6) | Egypt | North | Nasal mucosal | 2010-2011 | 3 | Culture | | 4-14 y | 4 | 14 | 9 | 3 | 84 | 3.57 | nd | nd | no |  | |
|  |  |  |  |  |  |  | | 4-14 y | 4 | 14 | 9 | 0 | 20 | 0 | no | nd | no |  | |
| Badawy 2017 (7) | Egypt | North | Nasopharyngeal | 2012-2014 | 3 | Culture | | 6 m - 5 y | 0.5 | 5 | 2.75 | 62 | 200 | 31 | no | nd | no |  | |
| El-Nawawy 2015 (8) | Egypt | North | Nasopharyngeal | 2013-2014 | 3 | Culture | | 2-60m | 0.17 | 5 | 2.585 | 175 | 600 | 29.17 | no | nd | no |  | |
| El-Kholy 2020 (9) | Egypt | North | Nasopharyngeal | 2015-2017 | 4 | PCR | | 6m-5y | 0.5 | 5 | 2.75 | 217 | 334 | 64.97 | no | nd | no |  | |
| Haug 2010 (10) | Ethiopia | East | Nasopharyngeal | 2005 | 2 | Culture | | 1-5 y | 1 | 5 | 3 | 112 | 120 | 93.33 | nd | nd | no |  | |
| Keenan 2014 and Skalet 2010 (11) | Ethiopia | East | Nasopharyngeal | 2007 | 2 | Culture | | 0-9 y | 0 | 9 | 4.5 | 98 | 120 | 81.67 | nd | nd | no |  | |
|  |  |  |  |  |  |  | | 0-9 y | 0 | 9 | 4.5 | 79 | 110 | 71.82 | nd | nd | no |  | |
| Assefa 2013 (12) | Ethiopia | East | Nasopharyngeal | 2012 | 3 | Culture | | 8-10 y | 8 | 10 | 9 | 21 | 63 | 33.33 | mix | nd | no |  | |
|  |  |  |  |  |  |  | | 5-<8 y | 5 | 8 | 6.5 | 24 | 60 | 40 | mix | nd | no |  | |
|  |  |  |  |  |  |  | | <3 y | 0 | 3 | 1.5 | 29 | 57 | 50.88 | mix | nd | nd |  | |
|  |  |  |  |  |  |  | | 3-<5 y | 3 | 5 | 4 | 22 | 54 | 40.74 | mix | nd | no |  | |
| Sime 2019 (13) | Ethiopia | East | Nasopharyngeal | 2013-2016 | 3 | Culture | | 6 w | 0.12 | 0.12 | 0.12 | 210 | 789 | 26.62 | nd | nd | no |  | |
| Gebre 2017 (14) | Ethiopia | East | Nasopharyngeal | 2014 | 3 | Culture | | 2-23 m | 0.17 | 1.92 | 1.045 | 80 | 192 | 41.67 | mix | nd | yes | PCV10 | |
|  |  |  |  |  |  |  | | 24-41 m | 2 | 3.42 | 2.71 | 41 | 93 | 44.09 | mix | nd | yes | PCV10 | |
|  |  |  |  |  |  |  | | 42-59 m | 3.5 | 4.92 | 4.21 | 37 | 76 | 48.68 | mix | nd | yes | PCV10 | |
| Wada 2019 (15) | Ethiopia | East | Nasopharyngeal | 2016 | 4 | Culture | | 6-13 y | 6 | 13 | 9.5 | 238 | 590 | 40.34 | nd | nd | nd |  | |
|  |  |  |  |  |  |  | | 3-5 y | 3 | 5 | 4 | 73 | 120 | 60.83 | nd | nd | nd |  | |
| Mulu 2018 (16) | Ethiopia | East | Pharyngeal | 2016-2017 | 4 | Culture | | 6-16 y | 6 | 16 | 11 | 31 | 300 | 10.33 | nd | HIV | nd |  | |
| Negash 2019 (17) | Ethiopia | East | Nasopharyngeal | 2016-2017 | 4 | Culture | | 28d-1y | 0.08 | 1 | 0.54 | 63 | 290 | 21.72 | yes | nd | yes | PCV10 | |
|  |  |  |  |  |  |  | | 2-5 y | 2 | 5 | 3.5 | 14 | 61 | 22.95 | yes | nd | yes | PCV10 | |
|  |  |  |  |  |  |  | | 6-15 y | 6 | 15 | 10.5 | 1 | 8 | 12.5 | yes | nd | yes | PCV10 | |
|  |  |  |  |  |  |  | | <28 d | 0 | 0.08 | 0.04 | 0 | 3 | 0 | yes | nd | no |  | |
| Abaye 2019 (18) | Ethiopia | East | Nasopharyngeal | 2017 | 4 | Culture | | 5 y | 5 | 5 | 5 | 41 | 233 | 17.6 | no | no | nd |  | |
|  |  |  |  |  |  |  | | 6 y | 6 | 6 | 6 | 36 | 163 | 22.09 | no | no | nd |  | |
|  |  |  |  |  |  |  | | 4 y | 4 | 4 | 4 | 9 | 74 | 12.16 | no | no | nd |  | |
|  |  |  |  |  |  |  | | <3 y | 0 | 3 | 1.5 | 2 | 7 | 28.57 | no | no | nd |  | |
| Abateneh 2020 (19) | Ethiopia | East | Nasopharyngeal | 2018 | 4 | Culture | | <3 y | 0 | 3 | 1.5 | 37 | 123 | 30.08 | mix | no | yes | PCV10 | |
|  |  |  |  |  |  |  | | 3-9 y | 3 | 9 | 6 | 31 | 118 | 26.27 | mix | no | yes | PCV10 | |
|  |  |  |  |  |  |  | | 9-15 y | 9 | 15 | 12 | 6 | 52 | 11.54 | mix | no | yes | PCV10 | |
| Haile 2019 (20) | Ethiopia | East | Nasopharyngeal | 2018 | 4 | Culture | | 5-6 y | 5 | 6 | 5.5 | 49 | 262 | 18.7 | no | nd | yes | PCV10 | |
|  |  |  |  |  |  |  | | 3-4y | 3 | 4 | 3.5 | 16 | 55 | 29.09 | no | nd | yes | PCV10 | |
| Seid 2020 (21) | Ethiopia | East | Nasopharyngeal | 2018 | 4 | Culture | | 30-44 y | 30 | 44 | 37 | 20 | 163 | 12.27 | nd | HIV | nd |  | |
|  |  |  |  |  |  |  | | 45-59 y | 45 | 59 | 52 | 7 | 52 | 13.46 | nd | HIV | nd |  | |
|  |  |  |  |  |  |  | | 16-29 y | 16 | 29 | 22.5 | 6 | 35 | 17.14 | nd | HIV | nd |  | |
| Hussen 2020 (22) | Ethiopia | East | Nasopharyngeal | 2018-2019 | 4 | Culture | | 3-23 m | 0.25 | 1.92 | 1.085 | 104 | 201 | 51.74 | nd | nd | yes | PCV10 | |
|  |  |  |  |  |  |  | | 42-59 m | 3.5 | 4.92 | 4.21 | 20 | 107 | 18.69 | nd | nd | yes | PCV10 | |
|  |  |  |  |  |  |  | | 24-41 m | 2 | 3.42 | 2.71 | 37 | 105 | 35.24 | nd | nd | yes | PCV10 | |
| Lemma 2020 (23) | Ethiopia | East | Nasopharyngeal | 2018-2019 | 4 | Culture | | 24-89 m | 2 | 7.42 | 4.71 | 38 | 97 | 39.18 | nd | mix | yes | PCV10 | |
| Scott 2005 (24) | Kenya | East | Nasopharyngeal | 2000-2013 | 2 | Culture | | <7 y | 0 | 7 | 3.5 | 40 | 97 | 41.24 | no | nd | no |  | |
|  |  |  |  |  |  |  | | <7 y | 0 | 7 | 3.5 | 31 | 95 | 32.63 | nd | nd | no |  | |
| Hamel 2008 (25) | Kenya | East | Nasopharyngeal | 2002-2003 | 1 | Culture | | 18-71 |  |  | 34 | 241 | 687 | 35.08 | nd | HIV | no |  | |
|  |  |  |  |  |  |  | | 16-74 |  |  | 31 | 112 | 334 | 33.53 | nd | HIV | no |  | |
|  |  |  |  |  |  |  | | 17-70 |  |  | 29 | 25 | 129 | 19.38 | nd | no | no |  | |
| Nyandiko 2007 (26) | Kenya | East | Nasopharyngeal | 2003 | 1 | Culture | | <5 y | 0 | 5 | 2.5 | 28 | 78 | 35.9 | mix | nd | no |  | |
| Abdullahi 2012 (27) | Kenya | East | Nasopharyngeal | 2006-2008 | 2 | Culture | | 3-23 m | 0.25 | 1.92 | 1.085 | 753 | 1015 | 74.19 | nd | no | no |  | |
|  |  |  |  |  |  |  | | 42-59 m | 3.5 | 4.92 | 4.21 | 534 | 937 | 56.99 | nd | no | no |  | |
|  |  |  |  |  |  |  | | 24-41 m | 2 | 3.42 | 2.71 | 581 | 888 | 65.43 | nd | no | no |  | |
|  |  |  |  |  |  |  | | 42-59 m | 3.5 | 4.92 | 4.21 | 29 | 35 | 82.86 | nd | HIV | no |  | |
|  |  |  |  |  |  |  | | 3-23 m | 0.25 | 1.92 | 1.085 | 22 | 33 | 66.67 | nd | HIV | no |  | |
|  |  |  |  |  |  |  | | 24-41 m | 2 | 3.42 | 2.71 | 24 | 31 | 77.42 | nd | HIV | no |  | |
| Tigoi 2012 (28) | Kenya | East | Nasopharyngeal | 2006-2008 | 2 | Culture | | 0 w | 0 | 0 | 0 | 80 | 1404 | 5.7 | nd | nd | no |  | |
| Conklin 2016 (29) | Kenya | East | Nasopharyngeal | 2009 | 2 | Culture | | 17-74 y |  |  | 32 | 237 | 549 | 43.17 | nd | HIV | no |  | |
|  |  |  |  |  |  |  | |  |  |  | 32 | 97 | 271 | 35.79 | nd | nd | no |  | |
|  |  |  |  |  |  |  | |  |  |  | 32 | 41 | 153 | 26.8 | nd | no | no |  | |
| da Gloria Carvalho 2013 (30) | Kenya | East | Nasopharyngeal | 2009 | 2 | Culture | | <5 y | 0 | 5 | 2.5 | 202 | 237 | 85.23 | nd | nd | no |  | |
|  |  |  |  |  |  |  | |  |  |  |  |  |  |  |  |  |  |  | |
| Kobayashi 2017 (31) | Kenya | East | Nasopharyngeal | 2009-2010 | 2 | Culture | | 1-4 y | 1 | 4 | 2.5 | 530 | 582 | 91.07 | mix | nd | no |  | |
|  |  |  |  |  |  |  | | 1-4 y | 1 | 4 | 2.5 | 280 | 321 | 87.23 | mix | nd | no |  | |
|  |  |  |  |  |  |  | | <1 y | 0 | 1 | 0.5 | 147 | 158 | 93.04 | mix | nd | no |  | |
|  |  |  |  |  |  |  | | <1 y | 0 | 1 | 0.5 | 26 | 26 | 100 | mix | nd | no |  | |
| Kobayashi 2020 (32) | Kenya | East | Nasopharyngeal | 2009-2010 | 2 | Culture | | 1-4 y | 1 | 4 | 2.5 | 535 | 588 | 90.99 | mix | nd | no |  | |
|  |  |  |  | 2009-2010 | 2 | Culture | | Adults |  |  | 32 | 237 | 549 | 43.17 | mix | HIV | no |  | |
|  |  |  |  | 2013 | 3 | Culture | | Adults |  |  | 34 | 149 | 530 | 28.11 | mix | HIV | no |  | |
|  |  |  |  | 2012 | 3 | Culture | | Adults |  |  | 34 | 246 | 526 | 46.77 | mix | HIV | no |  | |
|  |  |  |  | 2011 | 3 | Culture | | Adults |  |  | 34 | 167 | 423 | 39.48 | mix | HIV | no |  | |
|  |  |  |  | 2009-2010 | 2 | Culture | | 1-4 y | 1 | 4 | 2.5 | 280 | 321 | 87.23 | mix | nd | no |  | |
|  |  |  |  | 2013 | 3 | Culture | | 1-4 y | 1 | 4 | 2.5 | 294 | 311 | 94.53 | mix | nd | mix | PCV10 | |
|  |  |  |  | 2012 | 3 | Culture | | 1-4 y | 1 | 4 | 2.5 | 266 | 286 | 93.01 | mix | nd | mix | PCV10 | |
|  |  |  |  | 2011 | 3 | Culture | | 1-4 y | 1 | 4 | 2.5 | 244 | 265 | 92.08 | mix | nd | no |  | |
|  |  |  |  | 2009-2010 | 2 | Culture | | <1 y | 0 | 1 | 0.5 | 193 | 207 | 93.24 | mix | nd | no |  | |
|  |  |  |  | 2013 | 3 | Culture | | 1-4 y | 1 | 4 | 2.5 | 148 | 174 | 85.06 | mix | nd | yes | PCV10 | |
|  |  |  |  | 2009-2010 | 2 | Culture | | Adults |  |  | 30 | 41 | 153 | 26.8 | mix | no | no |  | |
|  |  |  |  | 2012 | 3 | Culture | | 1-4 y | 1 | 4 | 2.5 | 138 | 152 | 90.79 | mix | nd | mix | PCV10 | |
|  |  |  |  | 2013 | 3 | Culture | | <1 y | 0 | 1 | 0.5 | 135 | 151 | 89.4 | mix | nd | yes | PCV10 | |
|  |  |  |  | 2011 | 3 | Culture | | Adults |  |  | 30 | 58 | 143 | 40.56 | mix | no | no |  | |
|  |  |  |  | 2011 | 3 | Culture | | <1 y | 0 | 1 | 0.5 | 119 | 137 | 86.86 | mix | nd | yes | PCV10 | |
|  |  |  |  | 2013 | 3 | Culture | | Adults |  |  | 30 | 29 | 135 | 21.48 | mix | no | no |  | |
|  |  |  |  | 2011 | 3 | Culture | | 1-4 y | 1 | 4 | 2.5 | 105 | 131 | 80.15 | mix | nd | mix | PCV10 | |
|  |  |  |  | 2012 | 3 | Culture | | Adults |  |  | 30 | 45 | 131 | 34.35 | mix | no | no |  | |
|  |  |  |  | 2012 | 3 | Culture | | <1 y | 0 | 1 | 0.5 | 121 | 128 | 94.53 | mix | nd | yes | PCV10 | |
|  |  |  |  | 2009-2010 | 2 | Culture | | <1 y | 0 | 1 | 0.5 | 38 | 40 | 95 | mix | nd | no |  | |
|  |  |  |  | 2013 | 3 | Culture | | <1 y | 0 | 1 | 0.5 | 26 | 29 | 89.66 | mix | nd | yes | PCV10 | |
|  |  |  |  | 2012 | 3 | Culture | | <1 y | 0 | 1 | 0.5 | 20 | 25 | 80 | mix | nd | mix | PCV10 | |
|  |  |  |  | 2011 | 3 | Culture | | <1 y | 0 | 1 | 0.5 | 15 | 17 | 88.24 | mix | nd | yes | PCV10 | |
| Hammit 2014a (33) | Kenya | East | Nasopharyngeal | 2010 | 3 | Culture | | 12-59 m | 1 | 4.92 | 2.96 | 392 | 600 | 65.33 | nd | nd | no |  | |
| Hammit 2014b (34) | Kenya | East | Nasopharyngeal | 2011-2012 | 3 | Culture | | <5 y | 0 | 5 | 2.5 | 213 | 315 | 67.62 | nd | nd | mix | PCV10 | |
|  |  |  |  | 2009-2010 | 2 | Culture | | <5 y | 0 | 5 | 2.5 | 229 | 308 | 74.35 | nd | nd | no |  | |
| Feazel 2015 (35) | Kenya | East | Nasopharyngeal | 2010 | 3 | Culture | | 2-59m | 0.17 | 4.92 | 2.545 | 35 | 54 | 64.81 | nd | nd | no |  | |
| Gitthi 2013 (36) | Kenya | East | Nasopharyngeal | 2010 | 3 | Culture | | <5 y | 0 | 5 | 2.5 | 55 | 315 | 17.46 | nd | nd | no |  | |
| Hammit 2019 (37) | Kenya | East | Nasopharyngeal | 2012-2016 | 3 | Culture | | ≥15 | 15 | 60 | 37.5 | 287 | 1259 | 22.8 | mix | nd | no |  | |
|  |  |  |  | 2012-2016 | 3 | Culture | | <5 y | 0 | 5 | 2.5 | 606 | 796 | 76.13 | mix | nd | yes | PCV10 | |
|  |  |  |  | 2009-2010 | 2 | Culture | | ≥15 | 15 | 60 | 37.5 | 123 | 513 | 23.98 | mix | nd | no |  | |
|  |  |  |  | 2012-2016 | 3 | Culture | | 5-14 y | 5 | 14 | 9.5 | 237 | 489 | 48.47 | mix | nd | no |  | |
|  |  |  |  | 2009-2010 | 2 | Culture | | <5 y | 0 | 5 | 2.5 | 229 | 308 | 74.35 | mix | nd | no |  | |
|  |  |  |  | 2009-2010 | 2 | Culture | | 5-14 y | 5 | 14 | 9.5 | 103 | 196 | 52.55 | mix | nd | no |  | |
| Farrar 2020 (38) | Kenya | East | Nasopharyngeal | 2012 | 3 | Culture | | Adults |  |  | 33.4 | 393 | 846 | 46.45 | nd | mix | no |  | |
| Heath 2018 (39) | Kenya | East | Nasopharyngeal | 2014 | 3 | Culture | | 4-7 y | 4 | 7 | 5.5 | 65 | 323 | 20.12 | nd | nd | no |  | |
| Walekhwa 2019 (40) | Kenya | East | Nasopharyngeal | 2017-2018 | 4 | Culture | | 6-12 m | 0.5 | 1 | 0.75 | 16 | 68 | 23.53 | nd | no | mix | PCV10 | |
|  |  |  |  |  |  |  | | 13-24 m | 1.08 | 2 | 1.54 | 8 | 47 | 17.02 | nd | no | mix | PCV10 | |
|  |  |  |  |  |  |  | | 25-36 m | 2.08 | 3 | 2.54 | 12 | 46 | 26.09 | nd | no | mix | PCV10 | |
|  |  |  |  |  |  |  | | 49-60 m | 4.08 | 5 | 4.54 | 2 | 28 | 7.14 | nd | no | mix | PCV10 | |
|  |  |  |  |  |  |  | | 37-48 m | 3.08 | 4 | 3.54 | 4 | 17 | 23.53 | nd | no | mix | PCV10 | |
| Dananché 2020 (41) | Madagascar | East | Nasopharyngeal | 2010-2014 | 3 | PCR | | 2-60 m | 0.17 | 5 | 2.585 | 68 | 80 | 85 | yes | no | no |  | |
|  |  |  |  |  |  |  | | 2-60 m | 0.17 | 5 | 2.585 | 40 | 62 | 64.52 | no | mix | no |  | |
| Bénet 2017 (42) | Madagascar | East | Nasopharyngeal | 2010-2014 | 3 | FTD | | 2-60 m | 0.17 | 5 | 2.585 | 68 | 80 | 85 | yes | no | no |  | |
|  |  |  |  |  |  |  | | 2-60 m | 0.17 | 5 | 2.585 | 40 | 60 | 66.67 | no | mix | no |  | |
| Vallès 2003 (43) | Mozambique | East | Nasopharyngeal | 2003 | 2 | Culture | | 1-2 y | 1 | 2 | 1.5 | 115 | 128 | 89.84 | mix | nd | no |  | |
|  |  |  |  |  |  |  | | 1m-1 y | 0.08 | 1 | 0.54 | 88 | 96 | 91.67 | mix | nd | no |  | |
|  |  |  |  |  |  |  | | 3-4 y | 3 | 4 | 3.5 | 45 | 61 | 73.77 | mix | nd | no |  | |
| Brotons 2017 (44) | Mozambique | East | Nasopharyngeal | 2006-2014 | 3 | PCR | | <5 y | 0 | 5 | 2.5 | 53 | 60 | 88.33 | no | nd | no |  | |
| Heinsbroek 2016 (45) | Malawi | East | Nasopharyngeal | 2009-2010 | 2 | Culture | | 6w | 0.12 | 0.12 | 0.12 | 56 | 183 | 30.6 | nd | mix | no |  | |
| Glennie 2013 (46) | Malawi | East | Nasopharyngeal | 2010-2011 | 3 | Culture | | 21-51y |  |  | 30 | 4 | 32 | 12.5 | nd | no | no |  | |
|  |  |  |  |  |  |  | | 22-51y |  |  | 33 | 6 | 32 | 18.75 | nd | HIV | no |  | |
|  |  |  |  |  |  |  | | 21-52y |  |  | 34 | 9 | 27 | 33.33 | nd | HIV | no |  | |
|  |  |  |  |  |  |  | | 21-53y |  |  | 34 | 9 | 24 | 37.5 | nd | HIV | no |  | |
|  |  |  |  |  |  |  | | 26-51y |  |  | 38 | 11 | 21 | 52.38 | nd | HIV | no |  | |
| Massora 2019 | Mozambique | East | Nasopharyngeal | 2014-2015 | 3 | Culture | | 0-59 m | 0 | 4.92 | 2.46 | 507 | 592 | 85.64 | nd | mix | yes | PCV10 | |
| (47) |  |  |  | 2012-2013 | 3 | Culture | | 0-59 m | 0 | 4.92 | 2.46 | 411 | 506 | 81.23 | nd | mix | no |  | |
| Sigaúque 2018 (48) | Mozambique | East | Nasopharyngeal | 2014-2015 | 3 | Culture | | 24-59 m | 2 | 4.92 | 3.46 | 246 | 286 | 86.01 | nd | HIV | no |  | |
|  |  |  |  | 2012-2013 | 3 | Culture | | 24-59 m | 2 | 4.92 | 3.46 | 178 | 215 | 82.79 | nd | HIV | no |  | |
|  |  |  |  | 2014-2015 | 3 | Culture | | 24-59 m | 2 | 4.92 | 3.46 | 127 | 167 | 76.05 | nd | no | no |  | |
|  |  |  |  | 2012-2013 | 3 | Culture | | 24-59 m | 2 | 4.92 | 3.46 | 126 | 161 | 78.26 | nd | no | no |  | |
|  |  |  |  | 2012-2013 | 3 | Culture | | 12-23 m | 1 | 1.92 | 1.46 | 94 | 113 | 83.19 | nd | HIV | no |  | |
|  |  |  |  | 2014-2015 | 3 | Culture | | 12-23 m | 1 | 1.92 | 1.46 | 89 | 105 | 84.76 | nd | no | yes | PCV10 | |
|  |  |  |  | 2014-2015 | 3 | Culture | | 12-23 m | 1 | 1.92 | 1.46 | 72 | 87 | 82.76 | nd | HIV | yes | PCV10 | |
|  |  |  |  | 2012-2013 | 3 | Culture | | 6w-11 m | 0.12 | 0.92 | 0.52 | 65 | 86 | 75.58 | nd | HIV | no |  | |
|  |  |  |  | 2012-2013 | 3 | Culture | | 12-23 m | 1 | 1.92 | 1.46 | 58 | 74 | 78.38 | nd | no | no |  | |
|  |  |  |  | 2012-2013 | 3 | Culture | | 6w-11 m | 0.12 | 0.92 | 0.52 | 58 | 71 | 81.69 | nd | no | no |  | |
|  |  |  |  | 2014-2015 | 3 | Culture | | 6w-11 m | 0.12 | 0.92 | 0.52 | 61 | 63 | 96.83 | nd | no | yes | PCV10 | |
|  |  |  |  | 2014-2015 | 3 | Culture | | 6w-11 m | 0.12 | 0.92 | 0.52 | 40 | 47 | 85.11 | nd | HIV | yes | PCV10 | |
|  |  |  |  | 2014-2015 | 3 | Culture | | 12-23 m | 1 | 1.92 | 1.46 | 27 | 31 | 87.1 | nd | HIV | no |  | |
|  |  |  |  | 2014-2015 | 3 | Culture | | 6w-11 m | 0.12 | 0.92 | 0.52 | 11 | 13 | 84.62 | nd | HIV | no |  | |
|  |  |  |  | 2014-2015 | 3 | Culture | | 6w-11 m | 0.12 | 0.92 | 0.52 | 8 | 8 | 100 | nd | no | no |  | |
|  |  |  |  | 2014-2015 | 3 | Culture | | 24-59 m | 2 | 4.92 | 3.46 | 5 | 6 | 83.33 | nd | no | yes | PCV10 | |
|  |  |  |  | 2014-2015 | 3 | Culture | | 24-59 m | 2 | 4.92 | 3.46 | 2 | 3 | 66.67 | nd | HIV | yes | PCV10 | |
|  |  |  |  | 2014-2015 | 3 | Culture | | 12-23 m | 1 | 1.92 | 1.46 | 3 | 3 | 100 | nd | no | no |  | |
| Valenciano 2021 (49) | Mozambique | East | Nasopharyngeal | 2015-2016 | 4 | nd | | 24-59 m | 2 | 4.92 | 3.46 | 336 | 394 | 85.28 | nd | HIV | mix | PCV10 | |
|  |  |  |  | 2015-2016 | 4 | nd | | 24-59 m | 2 | 4.92 | 3.46 | 249 | 302 | 82.45 | nd | no | mix | PCV10 | |
|  |  |  |  | 2014-2015 | 3 | nd | | 24-59 m | 2 | 4.92 | 3.46 | 255 | 299 | 85.28 | nd | HIV | mix | PCV10 | |
|  |  |  |  | 2012-2013 | 3 | nd | | 24-59 m | 2 | 4.92 | 3.46 | 178 | 215 | 82.79 | nd | HIV | no |  | |
|  |  |  |  | 2015-2016 | 4 | nd | | 12-23 m | 1 | 1.92 | 1.46 | 157 | 184 | 85.33 | nd | no | mix | PCV10 | |
|  |  |  |  | 2014-2015 | 3 | nd | | 24-59 m | 2 | 4.92 | 3.46 | 141 | 183 | 77.05 | nd | no | mix | PCV10 | |
|  |  |  |  | 2012-2013 | 3 | nd | | 24-59 m | 2 | 4.92 | 3.46 | 126 | 161 | 78.26 | nd | no | no |  | |
|  |  |  |  | 2014-2015 | 3 | nd | | 12-23 m | 1 | 1.92 | 1.46 | 112 | 134 | 83.58 | nd | HIV | mix | PCV10 | |
|  |  |  |  | 2015-2016 | 4 | nd | | 12-23 m | 1 | 1.92 | 1.46 | 108 | 127 | 85.04 | nd | HIV | mix | PCV10 | |
|  |  |  |  | 2014-2015 | 3 | nd | | 12-23 m | 1 | 1.92 | 1.46 | 102 | 119 | 85.71 | nd | no | mix | PCV10 | |
|  |  |  |  | 2012-2013 | 3 | nd | | 12-23 m | 1 | 1.92 | 1.46 | 94 | 113 | 83.19 | nd | HIV | no |  | |
|  |  |  |  | 2014-2015 | 3 | nd | | 6w-11 m | 0.12 | 0.92 | 0.52 | 92 | 98 | 93.88 | nd | no | mix | PCV10 | |
|  |  |  |  | 2015-2016 | 4 | nd | | 6w-11 m | 0.12 | 0.92 | 0.52 | 65 | 88 | 73.86 | nd | HIV | mix | PCV10 | |
|  |  |  |  | 2012-2013 | 3 | nd | | 6w-11 m | 0.12 | 0.92 | 0.52 | 65 | 86 | 75.58 | nd | HIV | no |  | |
|  |  |  |  | 2014-2015 | 3 | nd | | 6w-11 m | 0.12 | 0.92 | 0.52 | 64 | 78 | 82.05 | nd | HIV | mix | PCV10 | |
|  |  |  |  | 2015-2016 | 4 | nd | | 6w-11 m | 0.12 | 0.92 | 0.52 | 68 | 78 | 87.18 | nd | no | mix | PCV10 | |
|  |  |  |  | 2012-2013 | 3 | nd | | 12-23 m | 1 | 1.92 | 1.46 | 58 | 74 | 78.38 | nd | no | no |  | |
|  |  |  |  | 2012-2013 | 3 | nd | | 6w-11 m | 0.12 | 0.92 | 0.52 | 58 | 71 | 81.69 | nd | no | no |  | |
| Verani 2018 (50) | Mozambique | East | Nasopharyngeal | 2012-2013 | 3 | Culture | | <5 y | 0 | 5 | 2.5 | 339 | 416 | 81.49 | nd | HIV | no |  | |
|  |  |  |  |  |  |  | | <5 y | 0 | 5 | 2.5 | 246 | 311 | 79.1 | nd | no | no |  | |
| Heinsbroek 2018 (51) | Malawi | East | Nasopharyngeal | 2014 | 3 | Culture | | 6w | 0.12 | 0.12 | 0.12 | 64 | 146 | 43.84 | nd | nd | no |  | |
|  |  |  |  |  |  |  | | 5-15y | 5 | 15 | 10 | 33 | 89 | 37.08 | nd | nd | no |  | |
| Adebanjo 2018 (52) | Mozambique | East | Nasopharyngeal | 2014-2016 | 4 | Culture | | <5 y | 0 | 5 | 2.5 | 783 | 927 | 84.47 | no | mix | yes | PCV10 | |
|  |  |  |  |  |  |  | | <5 y | 0 | 5 | 2.5 | 351 | 778 | 45.12 | yes | mix | yes | PCV10 | |
| Pimenta 2020 (53) | Mozambique | East | Nasopharyngeal | 2014-2015 | 3 | Culture | | <5 y | 0 | 5 | 2.5 | 145 | 205 | 70.73 | yes | nd | nd |  | |
|  |  |  |  |  |  |  | | <5 y | 0 | 5 | 2.5 | 90 | 120 | 75 | no | nd | nd |  | |
| Swarthout 2020 (54) | Malawi | East | Nasopharyngeal | 2015-2018 | 4 | Culture | | 3-5 y | 3 | 5 | 4 | 1820 | 2405 | 75.68 | nd | no | yes | PCV13 | |
|  |  |  |  |  |  |  | | Adults | 18 | 40 | 33.55 | 714 | 1770 | 40.34 | nd | HIV | no |  | |
|  |  |  |  |  |  |  | | 6-8 y | 6 | 8 | 7 | 403 | 695 | 57.99 | nd | no | no |  | |
| Abotsi 2021 (55) | Malawi_Zimbabwe | East | Nasopharyngeal | 2016-2018 | 4 | Culture | | 13-16 y | 13 | 16 | 14.5 | 81 | 168 | 48.21 | mix | HIV | no |  | |
|  |  |  |  |  |  |  | | 17-19 y | 17 | 19 | 18 | 35 | 131 | 26.72 | mix | HIV | no |  | |
|  |  |  |  |  |  |  | | 6-12 y | 6 | 12 | 9 | 57 | 111 | 51.35 | mix | HIV | no |  | |
| Gingsburg 2020 (56) | Mozambique | East | Nasopharyngeal swab/aspirate | 2017-2018 | 4 | PCR | | 2-23m | 0.17 | 1.92 | 1.045 | 84 | 97 | 86.6 | yes | nd | nd |  | |
| Saeed 2011 (57) | South Sudan | East | Nasopharyngeal | 2010 | 3 | Culture | | 2w-12m | 0.04 | 1 | 0.52 | 14 | 38 | 36.84 | no | no | no |  | |
| Batt 2003 (58) | Tanzania | East | Throat | 2000-2001 | 1 | Culture | | ≤7 y | 0 | 7 | 3.5 | 141 | 1315 | 10.72 | nd | nd | no |  | |
| Leung 2011 (59) | Tanzania | East | Nasopharyngeal_oropharyngeal | 2003 | 1 | Culture | | 1-5 y | 1 | 5 | 3 | 21 | 83 | 25.3 | no | nd | no |  | |
| Chochua 2016 (60) | Tanzania | East | Nasopharyngeal | 2008 | 2 | PCR | | 2m-10 y | 0.17 | 10 | 5.085 | 503 | 597 | 84.25 | yes | nd | no |  | |
|  |  |  |  |  |  |  | | 2m-10 y | 0.17 | 10 | 5.085 | 273 | 363 | 75.21 | no | nd | no |  | |
| Moyo 2012 (61) | Tanzania | East | Nasopharyngeal | 2010 | 3 | Culture | | <5 y | 0 | 5 | 2.5 | 105 | 300 | 35 | no | nd | no |  | |
| Ndossa 2015 (62) | Tanzania | East | Nasopharyngeal | 2013-2014 | 3 | Culture | | 2m -5y | 0.17 | 5 | 2.585 | 32 | 253 | 12.65 | mix | mix | yes | PCV13 | |
|  |  |  |  |  |  |  | | 2m -5y | 0.17 | 5 | 2.585 | 11 | 97 | 11.34 | mix | mix | no |  | |
| Emgård 2019 (63) | Tanzania | East | Nasopharyngeal | 2013-2015 | 3 | Culture | | <2y | 0 | 2 | 1 | 244 | 775 | 31.48 | mix | nd | yes | PCV13 | |
| Bloch 2017 (64) | Tanzania | East | Nasopharyngeal | 2014-2015 | 3 | Culture | | 1-59 m | 0.08 | 4.92 | 2.5 | 455 | 1047 | 43.46 | nd | nd | nd |  | |
| Hercik 2017 (65) | Tanzania | East | Nasopharyngeal_oropharyngeal | 2014-2015 | 3 | PCR | | ≥15y | 15 | 60 | 37.5 | 61 | 160 | 38.13 | yes | mix | no |  | |
|  |  |  |  |  |  |  | | 5-14y | 5 | 14 | 9.5 | 93 | 140 | 66.43 | yes | mix | nd |  | |
|  |  |  |  |  |  |  | | 1-<5y | 1 | 5 | 3 | 59 | 85 | 69.41 | yes | mix | nd |  | |
| Bloch 2020 (66) | Tanzania | East | Nasopharyngeal | 2015-2017 | 4 | Culture | | 1-59 m | 0.08 | 4.92 | 2.5 | 226 | 527 | 42.88 | mix | nd | nd |  | |
| Ngocho 2020 (67) | Tanzania | East | Nasopharyngeal | 2017 | 4 | PCR | | 2-59m | 0.17 | 4.92 | 2.545 | 151 | 324 | 46.6 | no | nd | yes | PCV13 | |
|  |  |  |  |  |  |  | | 2-59m | 0.17 | 4.92 | 2.545 | 44 | 109 | 40.37 | yes | nd | yes | PCV13 | |
| Manyahi 2020 (68) | Tanzania | East | Nasopharyngeal | 2017-2018 | 4 | Culture | | Adults |  |  | 35 | 20 | 257 | 7.78 | nd | HIV | nd |  | |
| Kateete 2012 (69) | Uganda | East | Nasopharyngeal | 2001-2002 | 1 | Culture | | 8m - 6y | 0.67 | 6 | 3.335 | 27 | 81 | 33.33 | nd | Sickle_cell | no |  | |
|  |  |  |  |  |  |  | | 1-6 y | 1 | 6 | 3.5 | 38 | 71 | 53.52 | mix | no | no |  | |
| Blossom 2006 (70) | Uganda | East | Throat | 2004-2005 | 1 | Culture | | 22-55 |  |  | 38.15 | 108 | 600 | 18 | nd | HIV | no |  | |
| Lindstrand 2016 (71) | Uganda | East | Nasopharyngeal | 2011 | 3 | Culture | | 6-59 m | 0.5 | 4.92 | 2.71 | 554 | 1006 | 55.07 | mix | nd | no |  | |
|  |  |  |  | 2009 | 2 | Culture | | 6-59 m | 0.5 | 4.92 | 2.71 | 315 | 567 | 55.56 | mix | nd | no |  | |
|  |  |  |  | 2008 | 2 | Culture | | 2-59 m | 0.17 | 4.92 | 2.545 | 88 | 150 | 58.67 | mix | nd | no |  | |
| Nackers 2017 (72) | Uganda | East | Nasopharyngeal | 2014 | 3 | Culture | | 5-14 y | 5 | 14 | 9.5 | 156 | 417 | 37.41 | nd | nd | no |  | |
|  |  |  |  |  |  |  | | <2 y | 0 | 2 | 1 | 291 | 387 | 75.19 | nd | nd | no |  | |
|  |  |  |  |  |  |  | | 2-4 y | 2 | 4 | 3 | 154 | 217 | 70.97 | nd | nd | no |  | |
| Rutebemberwa 2015 (73) | Uganda | East | Nasopharyngeal | 2018 | 4 | Culture | | 2-59 m | 0.17 | 4.92 | 2.545 | 89 | 182 | 48.9 | nd | nd | nd |  | |
| Camelo 2021 (74) | Zambia | East | Nasopharyngeal | 2011-2013 | 3 | PCR | | 1-59 m | 0.08 | 4.92 | 2.5 | 304 | 370 | 82.16 | no | no | no |  | |
|  |  |  |  |  |  |  | | 1-59 m | 0.08 | 4.92 | 2.5 | 260 | 333 | 78.08 | yes | no | no |  | |
|  |  |  |  |  |  |  | | 1-59 m | 0.08 | 4.92 | 2.5 | 114 | 148 | 77.03 | no | no | no |  | |
|  |  |  |  |  |  |  | | 1-59 m | 0.08 | 4.92 | 2.5 | 90 | 123 | 73.17 | yes | no | no |  | |
|  |  |  |  |  |  |  | | 1-59 m | 0.08 | 4.92 | 2.5 | 72 | 91 | 79.12 | yes | HIV | no |  | |
|  |  |  |  |  |  |  | | 1-59 m | 0.08 | 4.92 | 2.5 | 53 | 74 | 71.62 | no | HIV | no |  | |
| Ndip 2008 (75) | Cameroon | Middle | Throat | 2004-2005 | 1 | Culture | | 10-13 y | 10 | 13 | 11.5 | 11 | 122 | 9.02 | yes | nd | no |  | |
|  |  |  |  |  |  |  | | 14-17 y | 14 | 17 | 15.5 | 9 | 64 | 14.06 | yes | nd | no |  | |
|  |  |  |  |  |  |  | | 18-21 y | 18 | 21 | 19.5 | 10 | 14 | 71.43 | yes | nd | no |  | |
| Njuma 2020 (76) | Cameroon | Middle | Nasopharyngeal | 2015 | 4 | Culture | | 24-26 m | 2 | 2.17 | 2.085 | 426 | 689 | 61.83 | no | nd | yes | PCV13 | |
|  |  |  |  | 2013 | 3 | Culture | | 24-26 m | 2 | 2.17 | 2.085 | 114 | 198 | 57.58 | no | nd | no |  | |
| Birindwa 2018 (77) | DR Congo | Middle | Nasopharyngeal | 2014-2015 | 3 | Culture | | < 6 m | 0 | 0.5 | 0.25 | 29 | 302 | 9.6 | mix | nd | mix | PCV13 | |
|  |  |  |  |  |  | Culture | | 6-12 m | 0.5 | 1 | 0.75 | 46 | 184 | 25 | mix | nd | mix | PCV13 | |
|  |  |  |  |  |  | Culture | | 24-60 m | 2 | 5 | 3.5 | 56 | 183 | 30.6 | mix | nd | mix | PCV13 | |
|  |  |  |  |  |  | Culture | | 12-24 m | 1 | 2 | 1.5 | 32 | 125 | 25.6 | mix | nd | mix | PCV13 | |
| Birindwa 2020 (78) | DR Congo | Middle | Nasopharyngeal | 2014-2015 | 3 | PCR | | 2-60 m | 0.17 | 5 | 2.585 | 148 | 199 | 74.37 | mix | nd | yes | PCV13 | |
|  |  |  |  |  |  | PCR | | 2-60 m | 0.17 | 5 | 2.585 | 142 | 176 | 80.11 | mix | nd | no |  | |
| Uddén 2020 (79) | Angola | Middle | Nasopharyngeal | 2017 | 4 | Culture | | 4-12 y | 4 | 12 | 8 | 332 | 940 | 35.32 | nd | nd | nd |  | |
| Bere 2009 (80) | Burkina Faso | West | Anterior_nares | 2000-2001 | 1 | Culture | | 2-60 m | 0.17 | 5 | 2.585 | 436 | 860 | 50.7 | no | nd | no |  | |
| Hema-Ouangraoua 2019 (81) | Burkina Faso | West | Nasopharyngeal | 2014 | 3 | Culture | | 3-59 m | 0.25 | 4.92 | 2.585 | 190 | 430 | 44.19 | nd | nd | mix | PCV13 | |
| Bonko 2021 (82) | Burkina Faso | West | Nasopharyngeal | 2014-2018 | 4 | Culture | | 1-60 m | 0.08 | 5 | 2.54 | 5 | 629 | 0.79 | yes | nd | nd |  | |
| Kiemde 2018 (83) | Burkina Faso | West | Nasopharyngeal | 2015 | 4 | Culture | | <5 y | 0 | 5 | 2.5 | 5 | 153 | 3.27 | yes | nd | no |  | |
| Bountogo 2021 (84) | Burkina Faso | West | Nasopharyngeal | 2015-2017 | 4 | Culture | | < 5y | 0 | 5 | 2.5 | 145 | 493 | 29.41 | yes | nd | nd |  | |
|  |  |  |  |  |  |  | | < 5y | 0 | 5 | 2.5 | 126 | 379 | 33.25 | no | nd | nd |  | |
| Coulibaly 2020 (85) | Burkina Faso | West | Nasopharyngeal | 2017 | 4 | Culture | | 0-59 m | 0 | 4.92 | 2.46 | 69 | 172 | 40.12 | nd | nd | nd |  | |
|  |  |  |  |  |  |  | | 0-59 m | 0 | 4.92 | 2.46 | 32 | 111 | 28.83 | nd | nd | nd |  | |
| Adiku 2015 (86) | Ghana | West | Nasopharyngeal aspirate | 2001 | 1 | Culture | | 0-47m | 0 | 3.92 | 1.96 | 3 | 108 | 2.78 | yes | nd | no |  | |
| Donkor 2010 (87) | Ghana | West | Nasopharyngeal | 2006-2007 | 2 | Culture | | ≥5 - <13 y | 5 | 13 | 9 | 10 | 97 | 10.31 | no | no | no |  | |
|  |  |  |  |  |  | Culture | | <5 y | 0 | 5 | 2.5 | 9 | 33 | 27.27 | no | no | no |  | |
| Mills 2015 (88) | Ghana | West | Nasopharyngeal | 2011 | 3 | Culture | | 0-5 y | 0 | 5 | 2.5 | 207 | 422 | 49.05 | mix | nd | no |  | |
| Dayie 2013 (89) | Ghana | West | Nasopharyngeal | 2011 | 3 | Culture | | 48-59 m | 4 | 4.92 | 4.46 | 83 | 342 | 24.27 | no | nd | no |  | |
|  |  |  |  |  |  |  | | 36-47 m | 3 | 3.92 | 3.46 | 89 | 267 | 33.33 | no | nd | no |  | |
|  |  |  |  |  |  |  | | 24-35 m | 2 | 2.92 | 2.46 | 51 | 137 | 37.23 | no | nd | no |  | |
|  |  |  |  |  |  |  | | 12-23 m | 1 | 1.92 | 1.46 | 36 | 73 | 49.32 | no | nd | no |  | |
|  |  |  |  |  |  |  | | 0-11 m | 0 | 0.92 | 0.46 | 6 | 7 | 85.71 | no | nd | no |  | |
| Dayie 2018 (90) | Ghana | West | Nasopharyngeal | 2016-2017 | 4 | Culture | | 21-40 y | 21 | 40 | 30.5 | 11 | 112 | 9.82 | mix | Sickle_cell | no |  | |
|  |  |  |  |  |  |  | | ≤5y | 0 | 5 | 2.5 | 45 | 94 | 47.87 | mix | Sickle_cell | yes | PCV13 | |
|  |  |  |  |  |  |  | | >5-9 y | 5 | 9 | 7 | 26 | 75 | 34.67 | mix | Sickle_cell | no |  | |
|  |  |  |  |  |  |  | | 14-20 y | 14 | 20 | 17 | 3 | 46 | 6.52 | mix | Sickle_cell | no |  | |
|  |  |  |  |  |  |  | | ≥10-13 y | 10 | 13 | 11.5 | 8 | 33 | 24.24 | mix | Sickle_cell | no |  | |
|  |  |  |  |  |  |  | | 41-60 y | 41 | 60 | 50.5 | 2 | 25 | 8 | mix | Sickle_cell | no |  | |
|  |  |  |  |  |  |  | | ≥60y | 60 | 75 | 67.5 | 1 | 17 | 5.88 | mix | Sickle_cell | No |  | |
| Dayie 2019a (91) | Ghana | West | Nasopharyngeal | 2016 | 4 | Culture | | 36-47 m | 3 | 3.92 | 3.46 | 105 | 178 | 58.99 | no | nd | yes | PCV13 | |
|  |  |  |  |  |  |  | | 48-60 m | 4 | 4.92 | 4.46 | 64 | 135 | 47.41 | no | nd | yes | PCV13 | |
|  |  |  |  |  |  |  | | 24-35 m | 2 | 2.92 | 2.46 | 41 | 73 | 56.16 | no | nd | yes | PCV13 | |
|  |  |  |  |  |  |  | | 12-23 m | 1 | 1.92 | 1.46 | 9 | 22 | 40.91 | no | nd | yes | PCV13 | |
|  |  |  |  |  |  |  | | 0-11 m | 0 | 0.92 | 0.46 | 1 | 2 | 50 | no | nd | yes | PCV13 | |
| Dayie 2019b (92) | Ghana | West | Nasopharyngeal | 2016-2017 | 4 | Culture | | 41-50 y | 41 | 50 | 45.5 | 4 | 52 | 7.69 | nd | HIV | no |  | |
|  |  |  |  |  |  |  | | 31-40 y | 31 | 40 | 35.5 | 4 | 42 | 9.52 | nd | HIV | no |  | |
|  |  |  |  |  |  |  | | 5-10 y | 5 | 10 | 7.5 | 14 | 39 | 35.9 | nd | HIV | no |  | |
|  |  |  |  |  |  |  | | <5 y | 0 | 5 | 2.5 | 7 | 37 | 18.92 | nd | HIV | yes | PCV13 | |
|  |  |  |  |  |  |  | | 51-60 y | 51 | 60 | 55.5 | 1 | 24 | 4.17 | nd | HIV | no |  | |
|  |  |  |  |  |  |  | | 11-20 y | 11 | 20 | 15.5 | 5 | 22 | 22.73 | nd | HIV | no |  | |
|  |  |  |  |  |  |  | | 21-30 y | 21 | 30 | 25.5 | 2 | 21 | 9.52 | nd | HIV | no |  | |
|  |  |  |  |  |  |  | | 61-70 y | 61 | 70 | 65.5 | 0 | 6 | 0 | nd | HIV | no |  | |
|  |  |  |  |  |  |  | | 71-80 y | 71 | 80 | 75.5 | 0 | 2 | 0 | nd | HIV | no |  | |
| Mills 2020 (93) | Ghana | West | Nasopharyngeal | 2018 | 4 | Culture | | 25-30 m | 2.08 | 2.5 | 2.29 | 22 | 98 | 22.45 | no | nd | yes | PCV13 | |
|  |  |  |  |  |  |  | | 31-36 m | 2.58 | 3 | 2.79 | 23 | 79 | 29.11 | no | nd | yes | PCV13 | |
|  |  |  |  |  |  |  | | 19-24 m | 1.58 | 2 | 1.79 | 16 | 55 | 29.09 | no | nd | yes | PCV13 | |
|  |  |  |  |  |  |  | | 13-18 m | 1.08 | 1.5 | 1.29 | 18 | 54 | 33.33 | no | nd | yes | PCV13 | |
|  |  |  |  |  |  |  | | 49-54 m | 4.08 | 4.5 | 4.29 | 17 | 53 | 32.08 | no | nd | yes | PCV13 | |
|  |  |  |  |  |  |  | | 55-59 m | 4.58 | 4.92 | 4.75 | 14 | 50 | 28 | no | nd | yes | PCV13 | |
|  |  |  |  |  |  |  | | 7-12 m | 0.58 | 1 | 0.79 | 14 | 47 | 29.79 | no | nd | yes | PCV13 | |
|  |  |  |  |  |  |  | | 43-48 m | 3.58 | 4 | 3.79 | 17 | 45 | 37.78 | no | nd | yes | PCV13 | |
|  |  |  |  |  |  |  | | 37-42 m | 3.08 | 3.5 | 3.29 | 8 | 26 | 30.77 | no | nd | yes | PCV13 | |
|  |  |  |  |  |  |  | | 1-6 m | 0.08 | 0.5 | 0.29 | 2 | 6 | 33.33 | no | nd | yes | PCV13 | |
| Narwortey 2021 (94) | Ghana | West | Nasopharyngeal | 2018 | 4 | Culture | | 8-10 y | 8 | 10 | 9 | 25 | 78 | 32.05 | mix | nd | no |  | |
|  |  |  |  |  |  |  | | 5-7 y | 5 | 7 | 6 | 26 | 65 | 40 | mix | nd | no |  | |
|  |  |  |  |  |  |  | | 11-12 y | 11 | 12 | 11.5 | 12 | 50 | 24 | mix | nd | no |  | |
| Hansen 2017 (95) | Guinee Bissau | West | Nasopharyngeal | 2013-2014 | 3 | PCR | | <7 m | 0 | 0.58 | 0.29 | 411 | 512 | 80.27 | mix | nd | no |  | |
| Bénet 2015 (42) | Mali | West | Nasal | 2011-2012 | 3 | FTD | | 2-60 m | 0.17 | 5 | 2.585 | 85 | 118 | 72.03 | yes | mix | no |  | |
|  |  |  |  |  |  |  | | 2-60 m | 0.17 | 5 | 2.585 | 47 | 98 | 47.96 | no | no | no |  | |
| Bénet 2017 (96) | Mali | West | Nasopharyngeal | 2010-2014 | 3 | FTD | | 2-60 m | 0.17 | 5 | 2.585 | 85 | 118 | 72.03 | yes | mix | no |  | |
|  |  |  |  |  |  |  | | 2-60 m | 0.17 | 5 | 2.585 | 43 | 93 | 46.24 | no | no | no |  | |
| Dananché 2020 (41) | Mali | West | Nasopharyngeal | 2010-2014 | 3 | PCR | | 2-60 m | 0.17 | 5 | 2.585 | 85 | 118 | 72.03 | yes | mix | no |  | |
|  |  |  |  |  |  |  | | 2-60 m | 0.17 | 5 | 2.585 | 43 | 93 | 46.24 | no | no | no |  | |
| Hema-Ouangraoua 2019 (81) | Mali | West | Nasopharyngeal | 2014 | 3 | Culture | | 3-59 m | 0.25 | 4.92 | 2.585 | 227 | 342 | 66.37 | nd | nd | yes | PCV13 | |
| Ousmane 2017 ousman(97) | Niger | West | Nasopharyngeal | 2007-2008 | 2 | Culture | | 3-12 m | 0.25 | 1 | 0.625 | 384 | 642 | 59.81 | no | nd | no |  | |
|  |  |  |  |  |  |  | | ≤3 m | 0 | 0.25 | 0.125 | 150 | 335 | 44.78 | no | nd | no |  | |
|  |  |  |  |  |  |  | | ≥12-26 m | 1 | 2.17 | 1.585 | 117 | 216 | 54.17 | no | nd | no |  | |
| Lagare 2019 (98) | Niger | West | Nasopharyngeal | 2015 | 4 | PCR | | <5 y | 0 | 5 | 2.5 | 249 | 638 | 39.03 | yes | nd | nd |  | |
| Dano 2019 (99) | Niger | West | Nasopharyngeal | 2015-2016 | 4 | PCR | | 6-12 m | 0.5 | 1 | 0.75 | 66 | 161 | 40.99 | yes | nd | no |  | |
|  |  |  |  |  |  |  | | 12-24 m | 1 | 2 | 1.5 | 56 | 136 | 41.18 | yes | nd | no |  | |
|  |  |  |  |  |  |  | | 1-3 m | 0.08 | 0.25 | 0.165 | 36 | 128 | 28.13 | yes | nd | no |  | |
|  |  |  |  |  |  |  | | 4-6 m | 0.33 | 0.5 | 0.415 | 60 | 121 | 49.59 | yes | nd | no |  | |
|  |  |  |  |  |  |  | | 24-36 m | 2 | 3 | 2.5 | 16 | 53 | 30.19 | yes | nd | no |  | |
|  |  |  |  |  |  |  | | 36-59 m | 3 | 5 | 4 | 17 | 37 | 45.95 | yes | nd | no |  | |
| Ibrahim 2017 (100) | Niger | West | Nasopharyngeal | 2015-2017 | 4 | PCR | | 1-59 m | 0.08 | 4.92 | 2.5 | 239 | 612 | 39.05 | yes | nd | nd |  | |
| Nwachukwu 2008 (101) | Nigeria | West | Nasopharyngeal | 2007 | 2 | Culture | | 2-59 m | 0.17 | 4.92 | 2.545 | 49 | 71 | 69.01 | nd | nd | no |  | |
| Nweze 2012 (102) | Nigeria | West | Throat | 2007-2008 | 2 | Culture | | 11-14 y | 11 | 14 | 12.5 | 8 | 171 | 4.68 | nd | nd | no |  | |
|  |  |  |  |  |  |  | | 15-18 y | 15 | 18 | 16.5 | 6 | 89 | 6.74 | nd | nd | no |  | |
|  |  |  |  |  |  |  | | 19-23 y | 19 | 23 | 21 | 5 | 20 | 25 | nd | nd | no |  | |
| Adetifa 2018 (103) | Nigeria | West | Nasopharyngeal | 2016-2017 | 4 | Culture | | 0-4 y | 0 | 4 | 2 | 249 | 335 | 74.33 | mix | nd | no |  | |
|  |  |  |  |  |  |  | | 0-4 y | 0 | 4 | 2 | 270 | 295 | 91.53 | mix | nd | no |  | |
|  |  |  |  |  |  |  | | 5-17 y | 5 | 17 | 11 | 228 | 288 | 79.17 | Mix | nd | no |  | |
|  |  |  |  |  |  |  | | 18-34 y | 18 | 34 | 26 | 77 | 145 | 53.1 | mix | nd | no |  | |
|  |  |  |  |  |  |  | | >50 y | 50 | 75 | 62.5 | 30 | 141 | 21.28 | mix | nd | no |  | |
|  |  |  |  |  |  |  | | 18-34 y | 18 | 34 | 26 | 27 | 109 | 24.77 | mix | nd | no |  | |
|  |  |  |  |  |  |  | | 35-49 y | 35 | 49 | 42 | 19 | 84 | 22.62 | mix | nd | no |  | |
|  |  |  |  |  |  |  | | 35-49 y | 35 | 49 | 42 | 34 | 76 | 44.74 | mix | nd | no |  | |
|  |  |  |  |  |  |  | | >50 y | 50 | 75 | 62.5 | 33 | 66 | 50 | mix | nd | no |  | |
| Echave 2003 (104) | Senegal | West | Nasopharyngeal | 2000 | 1 | Culture | | 2-59 m | 0.17 | 4.92 | 2.545 | 47 | 87 | 54.02 | no | nd | no |  | |
|  |  |  |  |  |  |  | | 2-59 m | 0.17 | 4.92 | 2.545 | 17 | 27 | 62.96 | yes | nd | no |  | |
| Ba 2014 (105) | Senegal | West | Nasopharyngeal | 2007-2008 | 2 | Culture | | 0-2 y | 0 | 2 | 1 | 132 | 264 | 50 | nd | nd | no |  | |
| Tine 2018 (106) | Senegal | West | Nasopharyngeal | 2015 | 4 | PCR | | 1-5 y | 1 | 5 | 3 | 104 | 123 | 84.55 | yes | nd | nd |  | |
|  |  |  |  |  |  |  | | <1 y | 0 | 1 | 0.5 | 61 | 71 | 85.92 | yes | nd | nd |  | |
|  |  |  |  |  |  |  | | >15 y | 15 | 60 | 37.5 | 10 | 42 | 23.81 | yes | nd | no |  | |
|  |  |  |  |  |  |  | | 6-15 y | 6 | 15 | 10.5 | 10 | 14 | 71.43 | yes | nd | no |  | |
| Knobbe 2019 (107) | Senegal | West | Oropharyngeal | 2018 | 4 | Culture | | <5 y | 0 | 5 | 2.5 | 49 | 102 | 48.04 | yes | nd | yes | PCV13 | |
|  |  |  |  |  |  |  | | <5 y | 0 | 5 | 2.5 | 15 | 96 | 15.63 | no | nd | yes | PCV13 | |
| Hill 2006 (108) | The Gambia | West | Nasopharyngeal | 2003-2004 | 1 | Culture | | 5-14 y | 5 | 14 | 9.5 | 621 | 735 | 84.49 | nd | nd | no |  | |
|  |  |  |  |  |  |  | | 0-5 y | 0 | 5 | 2.5 | 621 | 666 | 93.24 | nd | nd | no |  | |
| Roca 2011 (109) | The Gambia | West | Nasopharyngeal | 2007 | 2 | Culture | | ≥15 y |  |  | 35 | 97 | 333 | 29.13 | nd | nd | no |  | |
|  |  |  |  |  |  |  | | ≥15 y |  |  | 37 | 73 | 316 | 23.1 | nd | nd | yes | PCV7 | |
|  |  |  |  |  |  |  | | 5-<15 y | 5 | 15 | 10 | 115 | 219 | 52.51 | nd | nd | no |  | |
|  |  |  |  |  |  |  | | 5-<15 y | 5 | 15 | 10 | 84 | 203 | 41.38 | nd | nd | yes | PCV7 | |
|  |  |  |  |  |  |  | | 2-<5 y | 2 | 5 | 3.5 | 45 | 70 | 64.29 | nd | nd | no |  | |
|  |  |  |  |  |  |  | | 2-<5 y | 2 | 5 | 3.5 | 40 | 69 | 57.97 | nd | nd | yes | PCV7 | |
| Usuf 2015 (110) | The Gambia | West | Nasopharyngeal | 2009 | 2 | Culture | | <5 y | 0 | 5 | 2.5 | 371 | 515 | 72.04 | nd | nd | no |  | |
| Burr 2014 (111) | The Gambia | West | Nasopharyngeal | 2010 | 3 | Culture | | 0-10 | 0 | 10 | 5 | 136 | 182 | 74.73 | nd | nd | mix | PCV7 | |
| Roca 2013 (112) | The Gambia | West | Nasopharyngeal | 2010 | 3 | Culture | | ≥15 y | 15 | 60 | 37.5 | 35 | 216 | 16.2 | nd | no | yes | PCV7 | |
|  |  |  |  |  |  |  | | ≥15 y | 15 | 60 | 37.5 | 35 | 182 | 19.23 | nd | nd | no |  | |
|  |  |  |  |  |  |  | | 2-<5 y | 2 | 5 | 3.5 | 75 | 112 | 66.96 | nd | nd | yes | PCV7 | |
|  |  |  |  |  |  |  | | 5-<15 y | 5 | 15 | 10 | 35 | 111 | 31.53 | nd | nd | yes | PCV7 | |
|  |  |  |  |  |  |  | | 5-<15 y | 5 | 15 | 10 | 49 | 103 | 47.57 | nd | nd | no |  | |
|  |  |  |  |  |  |  | | 2-<5 y | 2 | 5 | 3.5 | 59 | 88 | 67.05 | nd | nd | yes | PCV7 | |
| Foster-Nyarko 2016 (113) | The Gambia | West | Nasopharyngeal | 2011-2012 | 3 | Culture | | 2m-10 y | 0.17 | 10 | 5.085 | 729 | 1170 | 62.31 | nd | nd | nd |  | |
| Odutola 2017 (114) | The Gambia | West | Nasopharyngeal | 2011-2012 | 3 | Culture | | 8-10 w | 0.15 | 0.19 | 0.17 | 770 | 1200 | 64.17 | nd | nd | no |  | |
| Usuf 2015 (115) | The Gambia | West | Nasopharyngeal | 2013-2014 | 3 | Culture | | mothers | 18 | 45 | 31.5 | 91 | 374 | 24.33 | nd | nd | no |  | |
|  |  |  |  |  |  |  | | 28d | 0.08 | 0.08 | 0.08 | 139 | 367 | 37.87 | nd | nd | no |  | |
| Bojang 2018 (116) | The Gambia | West | Nasopharyngeal | 2014-2015 | 3 | Culture | | 11-13 m | 0.92 | 1.08 | 1 | 193 | 235 | 82.13 | nd | nd | nd |  | |
| Dunne 2020 (117) | The Gambia | West | Nasopharyngeal | 2015-2016 | 3 | PCR | | <5 y | 0 | 5 | 2.5 | 21 | 22 | 95.45 | no | nd | yes | PCV13 | |
|  |  |  |  |  |  |  | | <5 y | 0 | 5 | 2.5 | 18 | 20 | 90 | yes | nd | yes | PCV13 | |
| Usuf 2019 (118) | The Gambia | West | Nasopharyngeal | 2016 | 4 | Culture | | 6-12 m | 0.5 | 1 | 0.75 | 301 | 351 | 85.75 | nd | nd | yes | PCV13 | |
|  |  |  |  |  |  |  | | mothers |  |  | 25 | 129 | 342 | 37.72 | nd | nd | no |  | |
| Kelly 2018 (119) | Botswana | South | Nasopharyngeal | 2012-2016 | 3 | PCR | | 1-23 m | 0.08 | 1.92 | 1 | 96 | 170 | 56.47 | mix | mix | mix | PCV13 | |
| McNally 2006 (120) | South Africa | South | Nasopharyngeal | 2001-2002 | 1 | Culture | | 1-59 m | 0.08 | 4.92 | 2.5 | 169 | 355 | 47.61 | yes | mix | no |  | |
| Pemba 2008 (121) | South Africa | South | Nasopharyngeal_oropharyngeal | 2002-2003 | 1 | Culture | | 40-49 | 40 | 49 | 44.5 | 37 | 373 | 9.92 | nd | HIV | no |  | |
|  |  |  |  |  |  |  | | 30-39 | 30 | 39 | 34.5 | 32 | 351 | 9.12 | nd | HIV | no |  | |
|  |  |  |  |  |  |  | | >49 | 50 | 75 | 62.5 | 5 | 115 | 4.35 | nd | HIV | no |  | |
|  |  |  |  |  |  |  | | 18-29 | 18 | 29 | 23.5 | 1 | 17 | 5.88 | nd | HIV | no |  | |
| Ditse 2013 (122) | South Africa | South | Nasopharyngeal | 2004-2007 | 2 | Culture | | 4-7 y | 4 | 7 | 5.5 | 107 | 212 | 50.47 | nd | no | mix | PCV9 | |
|  |  |  |  |  |  |  | | 4-7 y | 4 | 7 | 5.5 | 54 | 74 | 72.97 | nd | HIV | mix | PCV9 | |
| Madhi 2015 (123) | South Africa | South | Nasopharyngeal | 2005-2006 | 2 | Culture | | 6-12 w | 0.12 | 0.23 | 0.175 | 49 | 193 | 25.39 | no | HIV | yes | PCV7 | |
|  |  |  |  |  |  |  | | 6-12 w | 0.12 | 0.23 | 0.175 | 31 | 122 | 25.41 | no | no | yes | PCV7 | |
|  |  |  |  |  |  |  | | 6-12 w | 0.12 | 0.23 | 0.175 | 33 | 114 | 28.95 | no | no | yes | PCV7 | |
|  |  |  |  |  |  |  | | 6-12 w | 0.12 | 0.23 | 0.175 | 22 | 88 | 25 | no | HIV | yes | PCV7 | |
| Olwagen 2020 (124) | South Africa | South | Nasopharyngeal | 2005-2006 | 2 | PCR | | 9 m | 0.75 | 0.75 | 0.75 | 122 | 208 | 58.65 | nd | HIV | yes | PCV7 | |
|  |  |  |  |  |  |  | | 9 m | 0.75 | 0.75 | 0.75 | 121 | 173 | 69.94 | nd | no | yes | PCV7 | |
| Albrich 2014 (125) | South Africa | South | Nasopharyngeal | 2005-2007 | 2 | PCR | | ≥18 y |  |  | 36.7 | 109 | 222 | 49.1 | yes | HIV | no |  | |
| Nunes 2015 (126) | South Africa | South | Nasopharyngeal | 2009-2010 | 2 | Culture | | 6-12 w | 0.12 | 0.23 | 0.175 | 46 | 249 | 18.47 | nd | no | no |  | |
| Madhi 2020a (127) | South Africa | South | Nasopharyngeal | 2009-2012 | 3 | Culture | | 6-10 w | 0.12 | 0.19 | 0.155 | 72 | 300 | 24 | no | no | nd |  | |
|  |  |  |  |  |  |  | | 6-10 w | 0.12 | 0.19 | 0.155 | 24 | 101 | 23.76 | no | no | nd |  | |
|  |  |  |  |  |  |  | | 6-10 w | 0.12 | 0.19 | 0.155 | 23 | 83 | 27.71 | no | HIV | nd |  | |
| Nzenze 2013 (128) | South Africa | South | Nasopharyngeal | 2011 | 3 | Culture | | <2 y | 0 | 2 | 1 | 666 | 908 | 73.35 | nd | mix | mix | PCV7 | |
|  |  |  |  | 2011 | 3 | Culture | | 6-12 y | 6 | 12 | 9 | 242 | 484 | 50 | nd | mix | no |  | |
|  |  |  |  | 2011 | 3 | Culture | | 2-5 y | 2 | 5 | 3.5 | 307 | 422 | 72.75 | nd | mix | no |  | |
|  |  |  |  | 2009 | 2 | Culture | | <2 y | 0 | 2 | 1 | 331 | 399 | 82.96 | nd | mix | no |  | |
|  |  |  |  | 2009 | 2 | Culture | | 6-12 y | 6 | 12 | 9 | 186 | 310 | 60 | nd | mix | no |  | |
|  |  |  |  | 2011 | 3 | Culture | | 13-18 y | 13 | 18 | 15.5 | 35 | 285 | 12.28 | nd | mix | no |  | |
|  |  |  |  | 2009 | 2 | Culture | | 2-5 y | 2 | 5 | 3.5 | 219 | 273 | 80.22 | nd | mix | no |  | |
|  |  |  |  | 2009 | 2 | Culture | | 13-18 y | 13 | 18 | 15.5 | 36 | 158 | 22.78 | nd | mix | no |  | |
| Nzenze 2015 (129) | South Africa | South | Nasopharyngeal | 2012 | 3 | Culture | | mothers |  |  | 29.1 | 92 | 948 | 9.7 | nd | no | no |  | |
|  |  |  |  | 2010-2011 | 3 | Culture | | mothers |  |  | 30.4 | 144 | 704 | 20.45 | nd | HIV | no |  | |
|  |  |  |  | 2010-2011 | 3 | Culture | | mothers |  |  | 30.4 | 65 | 672 | 9.67 | nd | no | no |  | |
|  |  |  |  | 2012 | 3 | Culture | | mothers |  |  | 29.1 | 84 | 608 | 13.82 | nd | HIV | no |  | |
|  |  |  |  | 2012 | 3 | Culture | | 9-24 m | 0.75 | 2 | 1.375 | 273 | 447 | 61.07 | nd | no | yes | PCV13 | |
|  |  |  |  | 2012 | 3 | Culture | | <9 m | 0 | 0.75 | 0.375 | 175 | 344 | 50.87 | nd | no | yes | PCV13 | |
|  |  |  |  | 2012 | 3 | Culture | | 48-144 m | 4 | 12 | 8 | 185 | 290 | 63.79 | nd | HIV | no |  | |
|  |  |  |  | 2010-2011 | 3 | Culture | | >24-48 m | 2 | 4 | 3 | 185 | 262 | 70.61 | nd | HIV | no |  | |
|  |  |  |  | 2010-2011 | 3 | Culture | | 9-24 m | 0.75 | 2 | 1.375 | 169 | 253 | 66.8 | nd | no | yes | PCV7 | |
|  |  |  |  | 2010-2011 | 3 | Culture | | 48-144 m | 4 | 12 | 8 | 176 | 236 | 74.58 | nd | HIV | no |  | |
|  |  |  |  | 2010-2011 | 3 | Culture | | >24-48 m | 2 | 4 | 3 | 131 | 187 | 70.05 | nd | no | no |  | |
|  |  |  |  | 2012 | 3 | Culture | | >24-48 m | 2 | 4 | 3 | 117 | 182 | 64.29 | nd | HIV | no |  | |
|  |  |  |  | 2010-2011 | 3 | Culture | | <9 m | 0 | 0.75 | 0.375 | 98 | 170 | 57.65 | nd | no | yes | PCV7 | |
|  |  |  |  | 2012 | 3 | Culture | | >24-48 m | 2 | 4 | 3 | 110 | 166 | 66.27 | nd | no | yes | PCV7_PCV13 | |
|  |  |  |  | 2010-2011 | 3 | Culture | | 9-24 m | 0.75 | 2 | 1.375 | 98 | 155 | 63.23 | nd | HIV | no |  | |
|  |  |  |  | 2012 | 3 | Culture | | 9-24 m | 0.75 | 2 | 1.375 | 49 | 92 | 53.26 | nd | HIV | no |  | |
|  |  |  |  | 2010-2011 | 3 | Culture | | 48-144 m | 4 | 12 | 8 | 46 | 88 | 52.27 | nd | no | no |  | |
|  |  |  |  | 2012 | 3 | Culture | | 48-144 m | 4 | 12 | 8 | 39 | 76 | 51.32 | nd | no | no |  | |
|  |  |  |  | 2010-2011 | 3 | Culture | | <9 m | 0 | 0.75 | 0.375 | 27 | 60 | 45 | nd | HIV | mix | PCV7 | |
|  |  |  |  | 2012 | 3 | Culture | | <9 m | 0 | 0.75 | 0.375 | 18 | 52 | 34.62 | nd | HIV | mix | PCV13 | |
| Wright 2017 (130) | South Africa | South | Nasopharyngeal | 2012-2013 | 3 | PCR | | 0 d | 0 | 0 | 0 | 14 | 58 | 24.14 | nd | nd | no |  | |
| Vanker 2019 (131) | South Africa | South | Nasopharyngeal | 2012-2015 | 3 | Culture | | 6 m | 0.5 | 0.5 | 0.5 | 584 | 887 | 65.84 | nd | nd | yes | PCV13 | |
|  |  |  |  |  |  |  | | mothers |  |  | 25.8 | 167 | 881 | 18.96 | nd | nd | no |  | |
| Madhi 2020b (132) | South Africa | South | Nasopharyngeal | 2013 | 3 | Culture | | 9-23 m | 0.75 | 1.92 | 1.335 | 268 | 404 | 66.34 | mix | mix | yes | PCV7_PCV13 | |
|  |  |  |  |  |  |  | | 24-59 m | 2 | 4.92 | 3.46 | 175 | 259 | 67.57 | mix | mix | mix | PCV7_PCV13 | |
|  |  |  |  |  |  |  | | 60-144 m | 5 | 12 | 8.5 | 130 | 251 | 51.79 | mix | mix | no |  | |
|  |  |  |  |  |  |  | | 12-18 y | 12 | 18 | 15 | 27 | 177 | 15.25 | mix | mix | no |  | |
|  |  |  |  |  |  |  | | ≤ 9m | 0 | 0.75 | 0.375 | 14 | 30 | 46.67 | mix | mix | yes | PCV7_PCV13 | |
| Velaphi 2019 (133) | South Africa | South | Nasopharyngeal_oropharyngeal | 2013-2014 | 3 | PCR | | 0-27 d | 0 | 0.08 | 0.04 | 27 | 1204 | 2.24 | yes | nd | no |  | |
|  |  |  |  |  |  |  | | 0-27 d | 0 | 0.08 | 0.04 | 15 | 303 | 4.95 | no | nd | no |  | |
| Skosana 2021 (134) | South Africa | South | Nasopharyngeal | 2014 and 2016 | 3 | Culture | | 6-14 w | 0.12 | 0.27 | 0.195 | 49 | 153 | 32.03 | no | nd | yes | PCV13 | |
|  |  |  |  |  |  |  | | 18 m | 1.5 | 1.5 | 1.5 | 30 | 63 | 47.62 | no | nd | yes | PCV13 | |
|  |  |  |  |  |  |  | | 9 m | 0.75 | 0.75 | 0.75 | 38 | 61 | 62.3 | no | nd | yes | PCV13 | |

Sup table S3: Serotyping data

| Reference | Country | Year | Serotyping method | Age group | Lung_infection | Underlying_  disease | Vaccination status | Vaccine type | Total isola | PCV7 serotypes | PCV10 serotypes | PCV13 serotypes | non_PCV7 | non_PCV10 | non_PCV13 | Serotype | | | | | | | | | | | | |
| --- | --- | --- | --- | --- | --- | --- | --- | --- | --- | --- | --- | --- | --- | --- | --- | --- | --- | --- | --- | --- | --- | --- | --- | --- | --- | --- | --- | --- |
|  |  |  |  |  |  |  |  |  |  |  |  |  |  |  |  | 4 | 6B | 9V | 14 | 18C | 19F | 23F | 1 | 5 | 7F | 3 | 6A | 19A |
| Warda 2013 (1) | Morocco | 2007-2008 | Latex agglutination | 1-24m | nd | nd | no |  | 163 | 86 | 88 | 99 | 52.8 | 54.0 | 60.7 | 1 | 25 (no subgroup) | 5 (no subgroup) | 13 | 7 (no subgroup) | 19 | 16 (no subgroup) | 2 | ND | ND | ND |  | 11 |
| Jroundi 2017 (2) | Moroco | 2010-2011 | RT-mPCR | 2-59m | no | nd | mix | PCV13 | 79 | 27 | 27 | 40 | 34.2 | 34.2 | 50.6 | 1 | 10 | 1 (9V/A/N/L) | 0 | 1 (18C/B) | 11 (19F/B/C) | 3 | 0 | 0 | 0 | 0 | 13 | 0 |
|  |  |  |  | 2-59m | yes | nd | no |  | 159 | 65 | 67 | 112 | 40.9 | 42.1 | 70.4 | 0 | 21 | 6 (9V/A/N/L) | 0 | 5 (18C/B) | 22 (19F/B/C) | 11 | 0 | 0 | 2 (7FA) | 1 | 34 | 10 |
| Dilagui 2019 (3) | Moroco | 2017 | PCR and Quellung for serogroup 6 and serogroup 9 | 2-18m | yes | nd | mix | PCV10 | 116 | 6 | 8 |  | 5.2 | 6.9 |  | 0 | 1 | 1 | 1 | 0 | 2 | 1 | 2 | 0 |  |  |  |  |
| Badawy 2017 (7) | Egypt | 2012-2014 | RT-mPCR | 6m-5y | no | nd | no |  | 113 |  | 66 | 73 | 0.0 | 58.4 | 64.6 | 0 | 21 (6A,B,C,D) | 4 | 1 | 4 | 19 | 3 | 11 | 3 | 0 | 1 |  | 6 |
| El-Nawawy 2015 (8) | Egypt | 2013-2014 | Quellung | 3-60m | no | nd | no |  | 175 | 92 | 96 | 118 | 52.6 | 54.9 | 67.4 | 0 | 25 | 6 | 8 | 8 | 43 | 2 | 0 | 0 | 4 (7A) | 0 | 19 | 3 |
| El-Kholy 2020 (9) | Egypt | 2015-2016 | mPCR | <5y | no | nd | no |  | 217 | 79 | 133 | 144 | 36.4 | 61.3 | 66.4 | 4 | 24 (6ABC) | 5 (9V/F) | 10 | 12 (18ABC) | 20 | 4 | 33 | 18 | 3 (7A/F) | 3 |  | 8 |
| Keenan 2014 (11) | Ethiopia | 2007 | sequential mPCR and Quellung | 0-9y | nd | nd | no |  | 82 |  | 19 | 29 |  | 23.2 | 35.4 |  |  |  |  |  |  |  |  |  |  |  |  |  |
| Sime 2019 (13) | Ethiopia | 2013-2016 | gel diffusion and/or Quellung | 6w | nd | nd | no |  | 208 | 35 | 42 | 63 | 16.8 | 20.2 | 30.3 | 4 | 4 | 3 | 7 | 0 | 7 | 10 | 0 | 7 | 0 | 9 | 7 | 5 |
| Negash 2019 (17) | Ethiopia | 2016-2017 | Quellung + PCR seqtyping | <5y | yes | nd | yes | PCV10 | 78 | 4 | 5 | 31 | 5.1 | 6.4 | 39.7 | 0 | 0 | 0 | 1 | 0 | 3 | 0 | 1 | 0 | 0 | 0 | 4 | 22 |
| Lemma 2020 (23) | Ethiopia | 2018-2019 | WGS | 24-89m | nd | HIV | yes | PCV10 | 25 | 1 | 2 | 5 | 4.0 | 8.0 | 20.0 | 0 | 0 | 0 | 0 | 0 | 0 | 1 | 0 | 0 | 1 | 0 | 1 | 2 |
|  |  |  |  | 24-89m | nd | no | yes | PCV10 | 13 | 0 | 0 | 1 | 0.0 | 0.0 | 7.7 | 0 | 0 | 0 | 0 | 0 | 0 | 0 | 0 | 0 | 0 | 0 | 1 | 0 |
| Abdullahi 2012 (27) | Kenya | 2006-2008 | Quellung | 3-59m | nd | HIV | no |  | 75 | 32 | 32 | 46 | 42.7 | 42.7 | 61.3 | 0 | 5 | 3 | 1 | 0 | 14 | 9 | 0 | 0 | 0 | 0 | 13 | 1 |
|  |  |  |  | 3-59m | nd | no | no |  | 1868 | 761 | 776 | 1100 | 40.7 | 41.5 | 58.9 | 12 | 184 | 51 | 85 | 29 | 283 | 117 | 13 | 1 | 1 | 34 | 237 | 53 |
| Conklin 2016 (29) | Kenya | 2009 | Latex agglutination + Quellung | 15-74y | nd | no | no |  | 41 |  | 18 | 24 |  | 43.9 | 58.5 |  |  |  |  |  |  |  |  |  |  |  |  |  |
|  |  |  |  |  | nd | HIV | no |  | 237 |  | 71 | 108 |  | 30.0 | 45.6 |  |  |  |  |  |  |  |  |  |  |  |  |  |
|  |  |  |  |  | nd | nd | no |  | 97 |  | 26 | 44 |  | 26.8 | 45.4 |  |  |  |  |  |  |  |  |  |  |  |  |  |
| da Gloria Carvalho 2013 (30) | Kenya | 2009 | Quellung | <5y | nd | nd | no |  | 202 | 79 | 89 | 108 | 39.1 | 44.1 | 53.5 | 3 | 13 | 2 | 11 | 1 | 30 | 19 | 6 | 2 | 2 | 3 | 15 | 1 |
| Kobayashi 2017 (31) | Kenya | 2009-2010 | Latex agglutination + Quellung or mPCR | 0-4y | mix | nd | no |  | 677 |  | 287 | 365 |  | 42.4 | 53.9 |  |  |  |  |  |  |  |  |  |  |  |  |  |
|  |  |  |  | 0-4y | mix | nd | no |  | 306 |  | 121 | 167 |  | 39.5 | 54.6 |  |  |  |  |  |  |  |  |  |  |  |  |  |
| Kobayashi 2020 (32) | Kenya | 2011 | mPCR | 1-4y | mix | nd | mix | PCV10 | 105 |  | 36 | 51 |  | 34.3 | 48.6 |  |  |  |  |  |  |  |  |  |  |  |  |  |
|  |  | 2012 | mPCR | <1y | mix | nd | mix | PCV10 | 20 |  | 4 | 7 |  | 20.0 | 35.0 |  |  |  |  |  |  |  |  |  |  |  |  |  |
|  |  | 2013 | mPCR | 1-4y | mix | nd | mix | PCV10 | 294 |  | 58 | 110 |  | 19.7 | 37.4 |  |  |  |  |  |  |  |  |  |  |  |  |  |
|  |  | 2012 | mPCR | 1-4y | mix | nd | mix | PCV10 | 266 |  | 51 | 104 |  | 19.2 | 39.1 |  |  |  |  |  |  |  |  |  |  |  |  |  |
|  |  | 2012 | mPCR | 1-4y | mix | nd | mix | PCV10 | 138 |  | 26 | 41 |  | 18.8 | 29.7 |  |  |  |  |  |  |  |  |  |  |  |  |  |
|  |  | 2009-2010 | Quellung or mPCR | adults | mix | no | no |  | 41 |  | 18 | 24 |  | 43.9 | 58.5 |  |  |  |  |  |  |  |  |  |  |  |  |  |
|  |  | 2009-2010 | Quellung or mPCR | 1-4y | mix | nd | no |  | 535 |  | 227 | 286 |  | 42.4 | 53.5 |  |  |  |  |  |  |  |  |  |  |  |  |  |
|  |  | 2009-2010 | Quellung or mPCR | <1y | mix | nd | no |  | 193 |  | 79 | 105 |  | 40.9 | 54.4 |  |  |  |  |  |  |  |  |  |  |  |  |  |
|  |  | 2009-2010 | Quellung or mPCR | 1-4y | mix | nd | no |  | 280 |  | 110 | 153 |  | 39.3 | 54.6 |  |  |  |  |  |  |  |  |  |  |  |  |  |
|  |  | 2011 | mPCR | 1-4y | mix | nd | no |  | 244 |  | 91 | 123 |  | 37.3 | 50.4 |  |  |  |  |  |  |  |  |  |  |  |  |  |
|  |  | 2009-2010 | Quellung or mPCR | <1y | mix | nd | no |  | 38 |  | 12 | 19 |  | 31.6 | 50.0 |  |  |  |  |  |  |  |  |  |  |  |  |  |
|  |  | 2011 | mPCR | adults | mix | no | no |  | 58 |  | 18 | 23 |  | 31.0 | 39.7 |  |  |  |  |  |  |  |  |  |  |  |  |  |
|  |  | 2009-2010 | Quellung or mPCR | adults | mix | HIV | no |  | 237 |  | 71 | 108 |  | 30.0 | 45.6 |  |  |  |  |  |  |  |  |  |  |  |  |  |
|  |  | 2012 | mPCR | Adults | mix | no | no |  | 45 |  | 9 | 18 |  | 20.0 | 40.0 |  |  |  |  |  |  |  |  |  |  |  |  |  |
|  |  | 2011 | mPCR | adults | mix | HIV | no |  | 167 |  | 33 | 61 |  | 19.8 | 36.5 |  |  |  |  |  |  |  |  |  |  |  |  |  |
|  |  | 2012 | mPCR | adults | mix | HIV | no |  | 246 |  | 34 | 86 |  | 13.8 | 35.0 |  |  |  |  |  |  |  |  |  |  |  |  |  |
|  |  | 2013 | mPCR | adults | mix | HIV | no |  | 149 |  | 15 | 51 |  | 10.1 | 34.2 |  |  |  |  |  |  |  |  |  |  |  |  |  |
|  |  | 2013 | mPCR | adults | mix | no | no |  | 29 |  | 1 | 6 |  | 3.4 | 20.7 |  |  |  |  |  |  |  |  |  |  |  |  |  |
|  |  | 2011 | mPCR | <1y | mix | nd | yes | PCV10 | 119 |  | 31 | 46 |  | 26.1 | 38.7 |  |  |  |  |  |  |  |  |  |  |  |  |  |
|  |  | 2011 | mPCR | <1y | mix | nd | yes | PCV10 | 15 |  | 3 | 4 |  | 20.0 | 26.7 |  |  |  |  |  |  |  |  |  |  |  |  |  |
|  |  | 2012 | mPCR | <1y | mix | nd | yes | PCV10 | 121 |  | 23 | 41 |  | 19.0 | 33.9 |  |  |  |  |  |  |  |  |  |  |  |  |  |
|  |  | 2013 | mPCR | <1y | mix | nd | yes | PCV10 | 135 |  | 22 | 50 |  | 16.3 | 37.0 |  |  |  |  |  |  |  |  |  |  |  |  |  |
|  |  | 2013 | mPCR | 1-4y | mix | nd | yes | PCV10 | 148 |  | 24 | 48 |  | 16.2 | 32.4 |  |  |  |  |  |  |  |  |  |  |  |  |  |
|  |  | 2013 | mPCR | <1y | mix | nd | yes | PCV10 | 26 |  | 3 | 6 |  | 11.5 | 23.1 |  |  |  |  |  |  |  |  |  |  |  |  |  |
| Hammit 2014a (33) | Kenya | 2010 | Latex agglutination | 12-59m | nd | nd | no |  | 392 |  | 175 |  |  | 44.6 |  |  |  |  |  |  |  |  |  |  |  |  |  |  |
| Hammit 2014b (34) | Kenya | 2011-2012 | Latex agglutination + Quellung | <5y | nd | nd | mix | PCV10 | 213 |  | 41 |  |  | 19.2 |  |  |  |  |  |  |  |  |  |  |  |  |  |  |
|  |  | 2011-2012 |  | >= 5y | nd | nd | mix | PCV10 | 204 |  | 25 |  |  | 12.3 |  |  |  |  |  |  |  |  |  |  |  |  |  |  |
|  |  | 2009-2010 |  | <5y | nd | nd | no |  | 229 |  | 104 |  |  | 45.4 |  |  |  |  |  |  |  |  |  |  |  |  |  |  |
|  |  | 2009-2010 |  | >= 5y | nd | nd | no |  | 226 |  | 59 |  |  | 26.1 |  |  |  |  |  |  |  |  |  |  |  |  |  |  |
| Githi 2013 (36) | Kenya | 2010 | Latex agglutination | ‘<5y | nd | nd | no |  | 55 | 23 | 26 | 38 | 41.8 | 47.3 | 69.1 | 0 | 4 |  | 4 (14A) | 0 | 7 | 8 | 2 | 1 | 0 | 1 | 9 | 2 |
| Hammit 2019 (37) | Kenya | 2009-2010 | Latex agglutination + Quellung | <5y | mix | nd | no |  | 229 |  | 104 |  |  | 45.4 |  |  |  |  |  |  |  |  |  |  |  |  |  |  |
|  |  | 2009-2010 |  | 5-14y | mix | nd | no |  | 103 |  | 30 |  |  | 29.1 |  |  |  |  |  |  |  |  |  |  |  |  |  |  |
|  |  | 2009-2010 |  | >=15y | mix | nd | no |  | 123 |  | 29 |  |  | 23.6 |  |  |  |  |  |  |  |  |  |  |  |  |  |  |
|  |  | 2012-2016 |  | 5-14y | mix | nd | no |  | 237 |  | 29 |  |  | 12.2 |  |  |  |  |  |  |  |  |  |  |  |  |  |  |
|  |  | 2012-2016 |  | >=15y | mix | nd | no |  | 287 |  | 17 |  |  | 5.9 |  |  |  |  |  |  |  |  |  |  |  |  |  |  |
|  |  | 2012-2016 |  | <5y | mix | nd | yes | PCV10 | 606 |  | 70 |  |  | 11.6 |  |  |  |  |  |  |  |  |  |  |  |  |  |  |
| Farrar 2020 (38) | Kenya | 2012 | Quellung | Adults | nd | mix | no |  | 393 |  | 64 | 134 |  | 16.3 | 34.1 |  |  |  |  |  |  |  |  |  |  |  |  |  |
| Heath 2018 (39) | Kenya | 2014 | PCR | 4-7y | nd | nd | no |  | 65 | 22 | 28 | 30 | 33.8 | 43.1 | 46.2 | 0 | 0 | 0 | 4 | 0 | 12 | 6 | 4 | 0 | 2 | 2 | 0 | 0 |
| Walekwha 2019 (40) | Kenya | 2017-2018 | Quellung | 49-60m | nd | no | mix | PCV10 | 2 | 0 | 0 | 1 | 0.0 | 0.0 | 50.0 | 0 | 0 | 0 | 0 | 0 | 0 | 0 | 0 | 0 | 0 | 0 | 1 | 0 |
|  |  |  |  | 37-48m | nd | no | mix | PCV10 | 4 | 0 | 0 | 2 | 0.0 | 0.0 | 50.0 | 0 | 0 | 0 | 0 | 0 | 0 | 0 | 0 | 0 | 0 | 0 | 2 | 0 |
|  |  |  |  | 6-12m | nd | no | mix | PCV10 | 16 | 0 | 0 | 4 | 0.0 | 0.0 | 25.0 | 0 | 0 | 0 | 0 | 0 | 0 | 0 | 0 | 0 | 0 | 2 | 2 | 0 |
|  |  |  |  | 25-36m | nd | no | mix | PCV10 | 12 | 0 | 0 | 2 | 0.0 | 0.0 | 16.7 | 0 | 0 | 0 | 0 | 0 | 0 | 0 | 0 | 0 | 0 | 2 | 0 | 0 |
|  |  |  |  | 13-24m | nd | no | mix | PCV10 | 8 | 0 | 0 | 0 | 0.0 | 0.0 | 0.0 | 0 | 0 | 0 | 0 | 0 | 0 | 0 | 0 | 0 | 0 | 0 | 0 | 0 |
| Dananche 2020 (41) | Madagascar | 2010-2014 | RT-mPCR | 2-60m | yes | no | no |  | 68 |  |  | 48 |  |  | 70.6 | 0 | 8 (6AB) | 7 | 4 |  | 8 | 1 | 3 | 21 | 2 | 0 |  | 4 |
|  |  |  |  | 2-60m | no | mix | no |  | 40 |  |  | 21 |  |  | 52.5 | 0 | 9 (6AB) | 1 | 3 |  | 3 | 4 | 1 | 5 | 0 | 2 |  | 3 |
|  | Mali | 2010-2014 |  | 2-60m | yes | mix | no |  | 85 |  |  | 54 |  |  | 63.5 | 0 | 22 (6AB) | 9 | 7 |  | 13 | 6 | 8 | 5 | 1 | 2 |  | 8 |
|  |  |  |  | 2-60m | no | no | no |  | 43 |  |  | 23 |  |  | 53.5 | 0 | 10 (6AB) | 3 | 3 |  | 2 | 3 | 0 | 0 | 0 | 3 |  | 4 |
| Brotons 2017 (44) | Mozambique | 2006-2014 | mPCR | <5y | no | nd | no |  | 53 |  | 31 | 38 |  | 58.5 | 71.7 |  |  |  |  |  |  |  |  |  |  |  |  |  |
| Massora 2019 (47) | Mozambique | 2012-2013 | Quellung + mPCR for non typeable | 0-59m | nd | mix | no |  | 411 | 171 | 172 | 239 | 41.6 | 41.8 | 58.2 | 6 | 23 | 9 | 17 | 5 | 59 | 52 | 1 | 0 | 0 | 7 | 43 | 17 |
|  |  | 2014-2015 |  | 0-59m | nd | mix | yes | PCV10 | 507 | 135 | 136 | 234 | 26.6 | 26.8 | 46.2 | 4 | 25 | 11 | 14 | 1 | 37 | 43 | 0 | 1 | 0 | 13 | 47 | 38 |
| Sigauque 2018 (48) | Mozambique | 2014-2015 | Quellung + mPCR for non typeable | 24-59m | nd | HIV | no |  | 178 |  | 84 | 115 |  | 47.2 | 64.6 |  |  |  |  |  |  |  |  |  |  |  |  |  |
|  |  | 2012-2013 |  | 6w-11m | nd | HIV | no |  | 65 |  | 30 | 42 |  | 46.2 | 64.6 |  |  |  |  |  |  |  |  |  |  |  |  |  |
|  |  | 2014-2015 |  | 24-59m | nd | no | no |  | 126 |  | 58 | 79 |  | 46.0 | 62.7 |  |  |  |  |  |  |  |  |  |  |  |  |  |
|  |  | 2012-2013 |  | 6w-11m | nd | no | no |  | 58 |  | 26 | 35 |  | 44.8 | 60.3 |  |  |  |  |  |  |  |  |  |  |  |  |  |
|  |  | 2014-2015 |  | 12-23m | nd | no | no |  | 58 |  | 26 | 34 |  | 44.8 | 58.6 |  |  |  |  |  |  |  |  |  |  |  |  |  |
|  |  | 2014-2015 |  | 24-59m | nd | no | no |  | 127 |  | 47 | 68 |  | 37.0 | 53.5 |  |  |  |  |  |  |  |  |  |  |  |  |  |
|  |  | 2012-2013 |  | 6w-11m | nd | HIV | no |  | 11 |  | 4 | 6 |  | 36.4 | 54.5 |  |  |  |  |  |  |  |  |  |  |  |  |  |
|  |  | 2014-2015 |  | 12-23m | nd | HIV | no |  | 27 |  | 9 | 12 |  | 33.3 | 44.4 |  |  |  |  |  |  |  |  |  |  |  |  |  |
|  |  | 2014-2015 |  | 12-23m | nd | HIV | no |  | 94 |  | 30 | 50 |  | 31.9 | 53.2 |  |  |  |  |  |  |  |  |  |  |  |  |  |
|  |  | 2014-2015 |  | 24-59m | nd | HIV | no |  | 246 |  | 78 | 128 |  | 31.7 | 52.0 |  |  |  |  |  |  |  |  |  |  |  |  |  |
|  |  | 2012-2013 |  | 6w-11m | nd | no | no |  | 8 |  | 2 | 3 |  | 25.0 | 37.5 |  |  |  |  |  |  |  |  |  |  |  |  |  |
|  |  | 2014-2015 |  | 12-23m | nd | no | no |  | 3 |  | 0 | 1 |  | 0.0 | 33.3 |  |  |  |  |  |  |  |  |  |  |  |  |  |
|  |  | 2014-2015 |  | 24-59m | nd | HIV | yes | PCV10 | 2 |  | 1 | 1 |  | 50.0 | 50.0 |  |  |  |  |  |  |  |  |  |  |  |  |  |
|  |  | 2014-2015 |  | 12-23m | nd | HIV | yes | PCV10 | 72 |  | 20 | 32 |  | 27.8 | 44.4 |  |  |  |  |  |  |  |  |  |  |  |  |  |
|  |  | 2014-2015 |  | 12-23m | nd | no | yes | PCV10 | 89 |  | 22 | 38 |  | 24.7 | 42.7 |  |  |  |  |  |  |  |  |  |  |  |  |  |
|  |  | 2012-2013 |  | 6w-11m | nd | no | yes | PCV10 | 61 |  | 13 | 32 |  | 21.3 | 52.5 |  |  |  |  |  |  |  |  |  |  |  |  |  |
|  |  | 2014-2015 |  | 24-59m | nd | no | yes | PCV10 | 5 |  | 1 | 2 |  | 20.0 | 40.0 |  |  |  |  |  |  |  |  |  |  |  |  |  |
|  |  | 2012-2013 |  | 6w-11m | nd | HIV | yes | PCV10 | 40 |  | 6 | 12 |  | 15.0 | 30.0 |  |  |  |  |  |  |  |  |  |  |  |  |  |
| Valenciano 2021 (49) | Mozambique | 2014-2015 | Quellung, mPCR for untypable | 24-59m | nd | no | mix | PCV10 | 141 |  | 50 | 74 |  | 35.5 | 52.5 |  |  |  |  |  |  |  |  |  |  |  |  |  |
|  |  | 2014-2015 |  | 24-59m | nd | HIV | mix | PCV10 | 255 |  | 81 | 134 |  | 31.8 | 52.5 |  |  |  |  |  |  |  |  |  |  |  |  |  |
|  |  | 2014-2015 |  | 12-23m | nd | HIV | mix | PCV10 | 112 |  | 32 | 49 |  | 28.6 | 43.8 |  |  |  |  |  |  |  |  |  |  |  |  |  |
|  |  | 2015-2016 |  | 24-59m | nd | HIV | mix | PCV10 | 336 |  | 94 | 166 |  | 28.0 | 49.4 |  |  |  |  |  |  |  |  |  |  |  |  |  |
|  |  | 2014-2015 |  | 6w-11m | nd | HIV | mix | PCV10 | 64 |  | 16 | 27 |  | 25.0 | 42.2 |  |  |  |  |  |  |  |  |  |  |  |  |  |
|  |  | 2014-2015 |  | 12-23m | nd | no | mix | PCV10 | 102 |  | 24 | 43 |  | 23.5 | 42.2 |  |  |  |  |  |  |  |  |  |  |  |  |  |
|  |  | 2015-2016 |  | 12-23m | nd | HIV | mix | PCV10 | 108 |  | 25 | 40 |  | 23.1 | 37.0 |  |  |  |  |  |  |  |  |  |  |  |  |  |
|  |  | 2015-2016 |  | 24-59m | nd | no | mix | PCV10 | 249 |  | 51 | 104 |  | 20.5 | 41.8 |  |  |  |  |  |  |  |  |  |  |  |  |  |
|  |  | 2014-2015 |  | 6w-11m | nd | no | mix | PCV10 | 92 |  | 18 | 42 |  | 19.6 | 45.7 |  |  |  |  |  |  |  |  |  |  |  |  |  |
|  |  | 2015-2016 |  | 12-23m | nd | no | mix | PCV10 | 157 |  | 22 | 57 |  | 14.0 | 36.3 |  |  |  |  |  |  |  |  |  |  |  |  |  |
|  |  | 2015-2016 |  | 6w-11m | nd | HIV | mix | PCV10 | 65 |  | 9 | 19 |  | 13.8 | 29.2 |  |  |  |  |  |  |  |  |  |  |  |  |  |
|  |  | 2015-2016 |  | 6w-11m | nd | no | mix | PCV10 | 68 |  | 9 | 19 |  | 13.2 | 27.9 |  |  |  |  |  |  |  |  |  |  |  |  |  |
|  |  | 2012-2013 |  | 24-59m | nd | HIV | no |  | 178 |  | 84 | 115 |  | 47.2 | 64.6 |  |  |  |  |  |  |  |  |  |  |  |  |  |
|  |  | 2012-2013 |  | 6w-11m | nd | HIV | no |  | 65 |  | 30 | 42 |  | 46.2 | 64.6 |  |  |  |  |  |  |  |  |  |  |  |  |  |
|  |  | 2012-2013 |  | 24-59m | nd | no | no |  | 126 |  | 58 | 79 |  | 46.0 | 62.7 |  |  |  |  |  |  |  |  |  |  |  |  |  |
|  |  | 2012-2013 |  | 6w-11m | nd | no | no |  | 58 |  | 26 | 35 |  | 44.8 | 60.3 |  |  |  |  |  |  |  |  |  |  |  |  |  |
|  |  | 2012-2013 |  | 12-23m | nd | no | no |  | 58 |  | 26 | 34 |  | 44.8 | 58.6 |  |  |  |  |  |  |  |  |  |  |  |  |  |
|  |  | 2012-2013 |  | 12-23m | nd | HIV | no |  | 94 |  | 30 | 50 |  | 31.9 | 53.2 |  |  |  |  |  |  |  |  |  |  |  |  |  |
| Verani 2018 (50) | Mozambique | 2012-2013 | Quellung, RT-PCR for untypable | <5y | nd | mix | no |  | 601 |  | 270 | 371 |  | 44.9 | 61.7 |  |  |  |  |  |  |  |  |  |  |  |  |  |
| Heinsbroek 2018 (51) | Malawi | 2014 | Latex agglutination | 6w | nd | nd | no |  | 64 |  |  | 19 |  |  | 29.7 |  |  |  |  |  |  |  |  |  |  |  |  |  |
|  |  | 2009-2011 | Quellung | 6w | nd | nd | no |  | 27 |  |  | 8 |  |  | 29.6 |  |  |  |  |  |  |  |  |  |  |  |  |  |
| Adebanjo 2018 (52) | Mozambique | 2014-2016 | Quellung + mPCR for non typeable | <5y | yes | mix | yes | PCV10 | 351 |  | 145 |  |  | 41.3 |  |  |  |  |  |  |  |  |  |  |  |  |  |  |
|  |  |  |  | <5y | no | mix | yes | PCV10 | 783 |  | 217 |  |  | 27.7 |  |  |  |  |  |  |  |  |  |  |  |  |  |  |
| Pimenta 2020 (53) | Mozambique | 2014-2015 | Quellung | <5y | no | nd | nd |  | 90 | 29 | 29 | 39 | 32.2 | 32.2 | 43.3 | 0 | 4 | 1 | 3 | 0 | 11 | 10 | 0 | 0 | 0 | 1 | 5 | 4 |
|  |  |  |  | <5y | yes | nd | nd |  | 145 | 28 | 28 | 61 | 19.3 | 19.3 | 42.1 | 1 | 4 | 2 | 5 | 1 | 7 | 8 | 0 | 0 | 0 | 1 | 9 | 23 |
| Swarthout 2020 (54) | Malawi | 2015-2018 | Latex agglutination | 6-8 y | nd | no | no |  | 403 |  |  | 128 |  |  | 31.8 |  |  |  |  |  |  |  |  |  |  |  |  |  |
|  |  |  |  | adults | nd | HIV | no |  | 714 |  |  | 217 |  |  | 30.4 |  |  |  |  |  |  |  |  |  |  |  |  |  |
|  |  |  |  | 3-5 y | nd | no | yes | PCV13 | 1820 |  |  | 444 |  |  | 24.4 |  |  |  |  |  |  |  |  |  |  |  |  |  |
| Saeed 2011 (57) | Sudan | 2010 | PCR | 2w-12m | no | no | no |  | 13 | 7 | 7 | 8 | 53.8 | 53.8 | 61.5 | 0 | 4 (6A/B) | 0 | 0 | 0 | 1 | 2 | 0 | 0 | 0 | 0 | 0 | 1 |
| Batt 2003 (58) | Tanzania | 2000 | Slide agglutination | <7y | nd | nd | no |  | 141 | 85 | 94 |  | 60.3 | 66.7 |  | 4 | 20 (A,B,C,D) | 15 (no subgroups) | 1 | 2 (no subgroup) | 29 (no subgroup) | 18 (no subgroup) | 2 | 1 | 6 (no subgroup) | 8 |  |  |
| Moyo 2012 (61) | Tanzania | 2010 | Quellung | <5y | no | nd | no |  | 115 | 64 | 64 | 73 | 55.7 | 55.7 | 63.5 |  |  |  |  |  |  |  |  |  |  |  |  |  |
| Emgard 2019 (63) | Tanzania | 2013-2015 | RT-mPCR + sequetyping | <2y | mix | nd | yes | PCV13 | 186 | 64 | 64 | 76 | 34.4 | 34.4 | 40.9 | 2 | 18 | 0 | 9 | 0 | 20 | 15 | 0 | 0 | 0 | 3 | 9 | 0 |
| Manyahi 2020 (66) | Tanzania | 2017-2018 | Latex agglutination | adults | nd | HIV | nd |  | 76 | 19 | 25 | 33 | 25.0 | 32.9 | 43.4 | 2 | 3 (no subgroups) | - | - | 3 (no subgroups) | 9 (no subgroups) | 2 (no subgroups) | - |  | 6 (no subgroups) | 8 (no subgroups) |  |  |
| Blossom 2006 (70) | Uganda | 2004-2005 | Quellung | adults | nd | HIV | no |  | 109 | 28 | 33 | 57 | 25.7 | 30.3 | 52.3 | 1 | 5 | 2 (no subgroups) | 3 | 3 | 7 | 7 | 0 | 1 | 4 (no subgroup) | 16 | 6 | 2 |
| Lindstrand 2016 (71) | Uganda | 2008-2011 | gel diffusion and/or Quellung | <5y | mix | nd | no |  | 957 |  |  |  |  | 42.0 | 54.0 |  |  |  |  |  |  |  |  |  |  |  |  |  |
| Nackers 2017 (72) | Uganda | 2014 | Latex agglutination + Quellung | <2y | nd | nd | no |  | 291 |  | 117 | 164 |  | 40.2 | 56.4 |  |  |  |  |  |  |  |  |  |  |  |  |  |
|  |  |  |  | 2-4y | nd | nd | no |  | 154 |  | 50 | 74 |  | 32.5 | 48.1 |  |  |  |  |  |  |  |  |  |  |  |  |  |
|  |  |  |  | 5-14y | nd | nd | no |  | 156 |  | 46 | 68 |  | 29.5 | 43.6 |  |  |  |  |  |  |  |  |  |  |  |  |  |
| Njuma Libwea 2020 (76) | Cameroon | 2013 | mPCR + Quellung when needed | 24-26m | no | nd | no |  | 114 | 33 | 33 | 42 | 28.9 | 28.9 | 36.8 | 0 | 5 | 1 | 2 | 1 | 14 | 10 | 0 | 0 | 0 | 1 | 7 | 1 |
|  |  | 2015 |  | 24-26m | no | nd | yes | PCV13 | 426 | 93 | 94 | 125 | 21.8 | 22.1 | 29.3 | 2 | 19 | 3 | 22 | 1 | 31 | 15 | 0 | 1 | 0 | 9 | 16 | 6 |
| Birindwa 2018 (77) | DR Congo | 2014-2015 | RT-mPCR or sequetyping | 1-60m | mix | nd | mix | PCV13 | 141 |  |  | 76 |  |  | 53.9 |  |  |  |  |  |  |  |  |  |  |  |  |  |
| Birindwa 2020 (78) | DR Congo | 2014-2015 | RT-mPCR | 2-60m | mix | nd | mix | PCV13 | 324 |  |  | 179 |  |  | 55.2 |  |  |  |  |  |  |  |  |  |  |  |  |  |
| Udden 2020 (79) | Angola | 2017 | mPCR combined with latex agglutination and Quellung | 4-12y | nd | nd | nd |  | 328 |  |  | 134 |  |  | 40.9 |  |  |  |  |  |  |  |  |  |  |  |  |  |
| Bere 2009 (80) | Burkina Faso | 2000-2001 | Quellung | 0-2m | no | nd | no |  | 8 | 4 |  |  | 50.0 |  |  | 4 | 0 | 0 | 0 | 0 | 0 | 0 |  |  | 0 |  |  |  |
|  |  |  |  | 2-24m | no | nd | no |  | 216 | 120 |  |  | 55.6 |  |  | 4 | 48 (no subgroup) | 8 (no subgroup) | 4 | 12 (no subgroups) | 8 | 36 (no subgroups) | ND | ND | 8 (no subgroup) | ND |  |  |
|  |  |  |  | 24-60m | no | nd | no |  | 8 | 0 |  |  | 0.0 |  |  | 0 | 0 | 0 | 0 | 0 | 0 | 0 | ND | ND | 0 | ND |  |  |
| Dayie 2013 (89) | Ghana | 2011 | Latex agglutination + Quellung | 0-11m | no | nd | no |  | 6 | 4 | 4 | 5 | 66.7 | 66.7 | 83.3 | 0 | 1 | 0 | 0 | 0 | 1 | 2 | 0 | 0 | 0 | 0 | 1 | 0 |
|  |  |  |  | 12-23m | no | nd | no |  | 21 | 17 | 17 | 18 | 81.0 | 81.0 | 85.7 | 1 | 0 | 0 | 3 | 0 | 12 | 1 | 0 | 0 | 0 | 1 | 0 | 0 |
|  |  |  |  | 12-23m | no | nd | no |  | 16 | 10 | 10 | 11 | 62.5 | 62.5 | 68.8 | 0 | 3 | 0 | 0 | 0 | 5 | 2 | 0 | 0 | 0 | 0 | 1 | 0 |
|  |  |  |  | 0-11m | no | nd | no |  | 2 | 1 | 1 | 1 | 50.0 | 50.0 | 50.0 | 0 | 0 | 0 | 0 | 0 | 1 | 0 | 0 | 0 | 0 | 0 | 0 | 0 |
|  |  |  |  | 48-59m | no | nd | no |  | 50 | 17 | 17 | 27 | 34.0 | 34.0 | 54.0 | 0 | 4 | 1 | 1 | 2 | 1 | 8 | 0 | 0 | 0 | 2 | 6 | 2 |
|  |  |  |  | 48-59m | no | nd | no |  | 35 | 11 | 12 | 14 | 31.4 | 34.3 | 40.0 | 0 | 8 | 0 | 1 | 0 | 2 | 0 | 0 | 0 | 1 | 1 | 0 | 1 |
|  |  |  |  | 24-35m | no | nd | no |  | 22 | 7 | 7 | 15 | 31.8 | 31.8 | 68.2 | 0 | 0 | 1 | 2 | 0 | 4 | 0 | 0 | 0 | 0 | 2 | 5 | 1 |
|  |  |  |  | 24-35m | no | nd | no |  | 33 | 10 | 10 | 15 | 30.3 | 30.3 | 45.5 | 0 | 3 | 1 | 2 | 0 | 4 | 0 | 0 | 0 | 0 | 1 | 3 | 1 |
|  |  |  |  | 36-47m | no | nd | no |  | 43 | 12 | 12 | 15 | 27.9 | 27.9 | 34.9 | 2 | 2 | 0 | 0 | 2 | 3 | 3 | 0 | 0 | 0 | 0 | 1 | 2 |
|  |  |  |  | 36-47m | no | nd | no |  | 50 | 12 | 12 | 19 | 24.0 | 24.0 | 38.0 | 0 | 2 | 1 | 1 | 0 | 3 | 5 | 0 | 0 | 0 | 3 | 4 | 0 |
| Dayie 2018 (90) | Ghana | 2016-2017 | Latex agglutination + Quellung | 1-13y | mix | Sickle cell | mix | PCV13 | 71 | 14 | 15 | 21 | 19.7 | 21.1 | 29.6 | 0 | 2 | 0 | 3 | 0 | 5 | 4 | 0 | 0 | 1 | 4 | 2 | 0 |
|  |  |  |  | 14-82y | mix | Sickle cell | no |  | 14 | 0 | 1 | 2 | 0.0 | 7.1 | 14.3 | 0 | 0 | 0 | 0 | 0 | 0 | 0 | 0 | 0 | 1 | 1 | 0 | 0 |
| Dayie 2019a (91) | Ghana | 2016 | Latex agglutination + Quellung | 6-60m | no | nd | yes | PCV13 | 234 | 52 | 53 | 81 | 22.2 | 22.6 | 34.6 | 0 | 7 | 0 | 9 | 3 | 15 | 18 | 1 | 0 | 0 | 7 | 8 | 13 |
| Dayie 2019b (92) | Ghana | 2016-2017 | Latex agglutination + Quellung | 0.6-7 y | no | HIV | mix | PCV13 | 25 | 6 | 6 | 11 | 24.0 | 24.0 | 44.0 | 0 | 1 | 0 | 0 | 0 | 1 | 4 | 0 | 0 | 0 | 1 | 0 | 4 |
| Mills 2020 (93) | Ghana | 2018 | mPCR and Quellung | 1-59m | no | nd | yes | PCV13 | 151 | 46 | 46 | 58 | 30.5 | 30.5 | 38.4 | 0 | 14 | 3 | 4 | 1 | 11 | 13 | 0 | 0 | 0 | 6 | 6 | 0 |
| Narwortey 2021 (94) | Ghana | 2018 | Latex agglutination and Quellung | 5-12y | mix | nd | no |  | 39 | 8 | 8 | 13 | 20.5 | 20.5 | 33.3 | 1 | 1 | 0 | 1 | 3 | 2 | 0 | 0 | 0 | 0 | 2 | 3 | 0 |
| Ousmane 2017 (97) | Niger | 2007-2008 | SM-PCR (sequential mutliplex PCR) | 0-2y | no | nd | no |  | 654 | 185 | 192 | 216 | 28.3 | 29.4 | 33.0 | 3 | 59 (no subgroups) | 3 (9V/A) | 34 | 11 (no subgroups) | 35 | 40 | 6 | - | 1 (7F/A) | 3 |  | 21 |
| Adetifa 2018 (103) | Nigeria | 2016-2017 | Latex agglutination + Quellung | 0-4y | mix | nd | no |  | 260 |  | 130 | 179 |  | 50.0 | 68.8 |  | 35 | 5 | 12 |  | 46 | 25 |  |  |  | 8 | 26 | 18 |
|  |  |  |  | 0-4y | mix | nd | no |  | 271 | 123 | 130 | 184 | 45.4 | 48.0 | 67.9 | 8 | 15 | 7 | 9 | 5 | 48 | 31 |  |  |  |  | 42 |  |
|  |  |  |  | 5-17y | mix | nd | no |  | 123 |  | 50 | 71 |  | 40.7 | 57.7 |  | 11 | 4 | 3 |  | 10 | 14 |  |  |  | 3 | 11 | 7 |
|  |  |  |  | 18-34y | mix | nd | no |  | 27 |  | 9 | 12 |  | 33.3 | 44.4 |  | 2 | 0 | 1 |  | 3 | 2 |  |  |  | 1 | 2 | 0 |
|  |  |  |  | 35-49y | mix | nd | no |  | 19 |  | 6 | 8 |  | 31.6 | 42.1 |  | 1 | 0 | 2 |  | 2 | 0 |  |  |  | 1 | 1 | 0 |
|  |  |  |  | 5-17y | mix | nd | no |  | 228 | 65 | 71 | 109 | 28.5 | 31.1 | 47.8 | 6 | 8 | 10 | 5 | 5 | 16 | 15 |  |  |  |  | 17 |  |
|  |  |  |  | 35-49y | mix | nd | no |  | 34 | 7 | 9 | 12 | 20.6 | 26.5 | 35.3 | 0 | 1 | 0 | 5 | 0 | 1 | 0 |  |  |  |  | 1 |  |
|  |  |  |  | 18-34y | mix | nd | no |  | 77 | 19 | 20 | 27 | 24.7 | 26.0 | 35.1 | 3 | 1 | 3 | 1 | 2 | 7 | 2 |  |  |  |  | 4 |  |
|  |  |  |  | >50y | mix | nd | no |  | 32 |  | 8 | 18 |  | 25.0 | 56.3 |  | 0 | 0 | 2 |  | 0 | 3 |  |  |  | 6 | 2 | 2 |
|  |  |  |  | >50y | mix | nd | no |  | 33 | 2 | 2 | 7 | 6.1 | 6.1 | 21.2 | 0 | 0 | 0 | 0 | 0 | 1 | 1 |  |  |  |  | 2 |  |
| Ba 2014 (105) | Senegal | 2007-2008 | Latex agglutination + Quellung | <5y | nd | nd | no |  | 132 | 48 | 52 | 70 | 36.4 | 39.4 | 53.0 |  |  |  |  |  |  |  |  |  |  |  |  |  |
| Roca 2011 (109) | The Gambia | 2007 | Latex agglutination, equivocal results confirmed by Quellung | 2-5y | nd | nd | no |  | 45 | 19 |  |  | 42.2 |  |  |  |  |  |  |  |  |  |  |  |  |  |  |  |
|  |  |  |  | 5-<15y | nd | nd | no |  | 115 | 19 |  |  | 16.5 |  |  |  |  |  |  |  |  |  |  |  |  |  |  |  |
|  |  |  |  | >= 15y | nd | nd | no |  | 97 | 13 |  |  | 13.4 |  |  |  |  |  |  |  |  |  |  |  |  |  |  |  |
|  |  |  |  | 2-5y | nd | nd | yes | PCV7 | 40 | 15 |  |  | 37.5 |  |  |  |  |  |  |  |  |  |  |  |  |  |  |  |
|  |  |  |  | 5-<15y | nd | nd | yes | PCV7 | 84 | 3 |  |  | 3.6 |  |  |  |  |  |  |  |  |  |  |  |  |  |  |  |
|  |  |  |  | >= 15y | nd | nd | yes | PCV7 | 73 | 4 |  |  | 5.5 |  |  |  |  |  |  |  |  |  |  |  |  |  |  |  |
| Roca 2013 (112) | The Gambia | 2010 | Latex agglutination, equivocal results confirmed by Quellung | 5-15y | nd | nd | no |  | 49 | 11 |  |  | 22.4 |  |  |  |  |  |  |  |  |  |  |  |  |  |  |  |
|  |  |  |  | >=15y | nd | nd | no |  | 35 | 1 |  |  | 2.9 |  |  |  |  |  |  |  |  |  |  |  |  |  |  |  |
|  |  |  |  | 2.5<5y | nd | nd | Yes | PCV7 | 59 | 12 |  |  | 20.3 |  |  |  |  |  |  |  |  |  |  |  |  |  |  |  |
|  |  |  |  | 2.5<5y | nd | nd | yes | PCV7 | 75 | 10 |  |  | 13.3 |  |  |  |  |  |  |  |  |  |  |  |  |  |  |  |
|  |  |  |  | 5-15y | nd | nd | yes | PCV7 | 35 | 6 |  |  | 17.1 |  |  |  |  |  |  |  |  |  |  |  |  |  |  |  |
|  |  |  |  | >=15y | nd | nd | yes | PCV7 | 35 | 1 |  |  | 2.9 |  |  |  |  |  |  |  |  |  |  |  |  |  |  |  |
| Odutola 2017 (114) | The Gambia | 2011-2012 | Latex agglutination | 8-10w | nd | nd | no |  | 770 |  | 84 |  |  | 10.9 |  |  |  |  |  |  |  |  |  |  |  |  |  |  |
| Dunne 2020 (117) | The Gambia | 2006-2007 | Latex agglutination | <5y | yes | nd | yes | PCV13 | 18 | 6 | 9 | 12 | 33.3 | 50.0 | 66.7 | 0 | 0 | 1 | 4 | 0 | 1 | 0 | 2 | 1 | 0 | 2 | 1 | 0 |
|  |  |  |  | <5y | no | nd | yes | PCV13 | 21 | 5 | 5 | 8 | 23.8 | 23.8 | 38.1 | 0 | 0 | 0 | 3 | 0 | 2 | 0 | 0 | 0 | 0 | 1 | 1 | 1 |
| Usuf 2018 (118) | The Gambia | 2016 | Quellung | mothers | nd | nd | no |  | 129 | 11 | 12 | 19 | 8.5 | 9.3 | 14.7 | 2 | 0 | 1 | 2 | 1 | 4 | 1 | 0 | 0 | 1 | 6 | 0 | 1 |
|  |  |  |  | 6-12m | nd | nd | yes | PCV13 | 301 | 21 | 33 | 40 | 7.0 | 11.0 | 13.3 | 3 | 0 | 2 | 2 | 0 | 10 | 4 | 0 | 0 | 12 | 4 | 0 | 3 |
| McNally 2006 (120) | South Africa | 2001-2002 | Quellung | 1-59m | yes | no | no |  | 56 | 31 | 32 | 41 | 55.4 | 57.1 | 73.2 | 1 | 7 | 0 | 2 | 1 | 9 | 11 | 0 | 0 | 1 (no subgroup) | 1 | 5 | 3 |
|  |  |  |  | 1-9m | yes | HIV | no |  | 107 | 55 | 55 | 89 | 51.4 | 51.4 | 83.2 | 3 | 15 | 3 | 6 | 1 | 15 | 12 | 0 | 0 | 0 | 1 | 16 | 17 |
| Pemba 2008 (121) | South Africa | 2002-2003 | Quellung | adults | nd | HIV | no |  | 72 | 26 | 26 | 39 | 36.1 | 36.1 | 54.2 | 2 | 6 | 2 | 1 | 0 | 8 | 7 | 0 | 0 | 0 | 6 | 5 | 2 |
| Madhi 2015 (123) | South Africa | 2005-2006 | Quellung | 6-12w | no | no | yes | PCV7 | 33 | 7 |  |  | 21.2 |  |  |  |  |  |  |  |  |  |  |  |  |  |  |  |
|  |  |  |  | 6-12w | no | no | yes | PCV7 | 31 | 8 |  |  | 25.8 |  |  |  |  |  |  |  |  |  |  |  |  |  |  |  |
|  |  |  |  | 6-12w | no | HIV | yes | PCV7 | 22 | 9 |  |  | 40.9 |  |  |  |  |  |  |  |  |  |  |  |  |  |  |  |
|  |  |  |  | 6-12w | no | HIV | yes | PCV7 | 49 | 27 |  |  | 55.1 |  |  |  |  |  |  |  |  |  |  |  |  |  |  |  |
| Olwagen 2020 (124) | South Africa | 2005-2006 | Nanofluidic RT-qPCR | 9m | nd | HIV | yes | PCV7 | 122 | 78 |  |  | 63.9 |  |  |  |  |  |  |  |  |  |  |  |  |  |  |  |
|  |  |  |  | 9m | nd | no | yes | PCV7 | 121 | 63 |  |  | 52.1 |  |  |  |  |  |  |  |  |  |  |  |  |  |  |  |
| Madhi 2020 (127) | South Africa | 2009-2012 | Quellung | 6-10w | no | HIV | nd |  | 23 |  | 13 |  |  | 56.5 |  |  |  |  |  |  |  |  |  |  |  |  |  |  |
|  |  |  |  | 6-10w | no | no | nd |  | 25 |  | 14 |  |  | 56.0 |  |  |  |  |  |  |  |  |  |  |  |  |  |  |
|  |  |  |  | 6-10w | no | no | nd |  | 24 |  | 13 |  |  | 54.2 |  |  |  |  |  |  |  |  |  |  |  |  |  |  |
|  |  |  |  | 6-10w | no | no | nd |  | 30 |  | 14 |  |  | 46.7 |  |  |  |  |  |  |  |  |  |  |  |  |  |  |
|  |  |  |  | 6-10w | no | no | nd |  | 17 |  | 7 |  |  | 41.2 |  |  |  |  |  |  |  |  |  |  |  |  |  |  |
| Nzenze 2013 (128) | South Africa | 2011 | Quellung | <2y | nd | mix | mix | PCV7 | 666 | 213 |  |  | 32.0 |  |  |  |  |  |  |  |  |  |  |  |  |  |  |  |
|  |  | 2009 |  | <2y | nd | mix | no |  | 331 | 180 |  |  | 54.4 |  |  |  |  |  |  |  |  |  |  |  |  |  |  |  |
|  |  | 2009 |  | 2-5y | nd | mix | no |  | 219 | 97 |  |  | 44.3 |  |  |  |  |  |  |  |  |  |  |  |  |  |  |  |
|  |  | 2009 |  | 6-12y | nd | mix | no |  | 186 | 59 |  |  | 31.7 |  |  |  |  |  |  |  |  |  |  |  |  |  |  |  |
|  |  | 2009 |  | 13-18y | nd | mix | no |  | 36 | 9 |  |  | 25.0 |  |  |  |  |  |  |  |  |  |  |  |  |  |  |  |
|  |  | 2011 |  | 2-5y | nd | mix | no |  | 307 | 121 |  |  | 39.4 |  |  |  |  |  |  |  |  |  |  |  |  |  |  |  |
|  |  | 2011 |  | 6-12y | nd | mix | no |  | 242 | 61 |  |  | 25.2 |  |  |  |  |  |  |  |  |  |  |  |  |  |  |  |
|  |  | 2011 |  | 13-18y | nd | mix | no |  | 35 | 6 |  |  | 17.1 |  |  |  |  |  |  |  |  |  |  |  |  |  |  |  |
| Nzenze 2015 (129) | South Africa | 2012 | Quellung | <9m | nd | HIV | mix | PCV13 | 18 | 7 |  | 7 | 38.9 | 0.0 | 38.9 |  |  |  |  |  |  |  |  |  |  |  |  |  |
|  |  | 2010 |  | 9-24m | nd | HIV | no |  | 98 | 46 |  | 63 | 46.9 |  | 64.3 |  |  |  |  |  |  |  |  |  |  |  |  |  |
|  |  | 2010 |  | 24-48m | nd | HIV | no |  | 185 | 78 |  | 112 | 42.2 |  | 60.5 |  |  |  |  |  |  |  |  |  |  |  |  |  |
|  |  | 2010 |  | 24-48m | nd | no | no |  | 131 | 45 |  | 77 | 34.4 |  | 58.8 |  |  |  |  |  |  |  |  |  |  |  |  |  |
|  |  | 2010 |  | Mother | nd | no | no |  | 65 | 20 |  | 36 | 30.8 |  | 55.4 |  |  |  |  |  |  |  |  |  |  |  |  |  |
|  |  | 2010 |  | 48-144m | nd | HIV | no |  | 176 | 53 |  | 89 | 30.1 |  | 50.6 |  |  |  |  |  |  |  |  |  |  |  |  |  |
|  |  | 2010 |  | 48-144m | nd | no | no |  | 46 | 13 |  | 22 | 28.3 |  | 47.8 |  |  |  |  |  |  |  |  |  |  |  |  |  |
|  |  | 2010 |  | Mother | nd | HIV | no |  | 144 | 32 |  | 61 | 22.2 |  | 42.4 |  |  |  |  |  |  |  |  |  |  |  |  |  |
|  |  | 2012 |  | 48-144m | nd | no | no |  | 39 | 11 |  | 16 | 28.2 |  | 41.0 |  |  |  |  |  |  |  |  |  |  |  |  |  |
|  |  | 2012 |  | 9-24m | nd | HIV | no |  | 49 | 13 |  | 18 | 26.5 |  | 36.7 |  |  |  |  |  |  |  |  |  |  |  |  |  |
|  |  | 2012 |  | 48-144m | nd | HIV | no |  | 185 | 35 |  | 66 | 18.9 |  | 35.7 |  |  |  |  |  |  |  |  |  |  |  |  |  |
|  |  | 2012 |  | Mother | nd | HIV | no |  | 84 | 13 |  | 29 | 15.5 |  | 34.5 |  |  |  |  |  |  |  |  |  |  |  |  |  |
|  |  | 2012 |  | 24-48m | nd | HIV | no |  | 117 | 19 |  | 31 | 16.2 |  | 26.5 |  |  |  |  |  |  |  |  |  |  |  |  |  |
|  |  | 2012 |  | Mother | nd | no | no |  | 92 | 5 |  | 19 | 5.4 |  | 20.7 |  |  |  |  |  |  |  |  |  |  |  |  |  |
|  |  | 2012 |  | <9m | nd | no | yes | PCV13 | 175 | 33 |  | 47 | 18.9 |  | 26.9 |  |  |  |  |  |  |  |  |  |  |  |  |  |
|  |  | 2012 |  | 9-24m | nd | no | yes | PCV13 | 273 | 43 |  | 62 | 15.8 |  | 22.7 |  |  |  |  |  |  |  |  |  |  |  |  |  |
|  |  | 2010 |  | 9-24m | nd | no | yes | PCV7 | 169 | 70 |  | 109 | 41.4 |  | 64.5 |  |  |  |  |  |  |  |  |  |  |  |  |  |
|  |  | 2010 |  | <9m | nd | no | yes | PCV7 | 98 | 31 |  | 49 | 31.6 |  | 50.0 |  |  |  |  |  |  |  |  |  |  |  |  |  |
|  |  | 2010 |  | <9m | nd | HIV | yes | PCV7 | 27 | 7 |  | 9 | 25.9 |  | 33.3 |  |  |  |  |  |  |  |  |  |  |  |  |  |
|  |  | 2012 |  | 24-48m | nd | no | yes | PCV7_+PCV13 | 110 | 12 |  | 23 | 10.9 |  | 20.9 |  |  |  |  |  |  |  |  |  |  |  |  |  |
| Madhi 2020b (132) | South Africa | 2013 | Quellung | 24-59m | mix | mix | mix | PCV7-PCV13 | 175 | 38 |  | 51 | 21.7 | 0.0 | 29.1 |  |  |  |  |  |  |  |  |  |  |  |  |  |
|  |  |  |  | 5-12y | mix | mix | no |  | 130 | 25 |  | 39 | 19.2 |  | 30.0 |  |  |  |  |  |  |  |  |  |  |  |  |  |
|  |  |  |  | 12-18y | mix | mix | no |  | 27 | 6 |  | 8 | 22.2 |  | 29.6 |  |  |  |  |  |  |  |  |  |  |  |  |  |
|  |  |  |  | <= 9m | mix | mix | yes | PCV7-PCV13 | 14 | 2 |  | 6 | 14.3 |  | 42.9 |  |  |  |  |  |  |  |  |  |  |  |  |  |
|  |  |  |  | 9-23m | mix | mix | yes | PCV7-PCV13 | 268 | 54 |  | 74 | 20.1 |  | 27.6 |  |  |  |  |  |  |  |  |  |  |  |  |  |
| Skosana 2021 (134) | South Africa | 2014 and 2016 | Quellung and RT-PCR | 6w-5y | no | nd | yes | PCV13 | 121 | 11 | 11 | 22 | 9.1 | 9.1 | 18.2 | 0 | 1 | 0 | 0 | 0 | 10 | 0 | 0 | 0 | 0 | 7 | 1 | 3 |

Sup table S4: Antimicrobial sensitivity data

| Paper | Age group | Country | Antibiotic | Resistant numerator | Resistant denominator | Resistant Percentage | Intermediate numerator | Intermediate denominator | Intermediate Percentage | Susceptible numerator | Susceptible denominator | Susceptible Percentage | Percentage non-susceptible |
| --- | --- | --- | --- | --- | --- | --- | --- | --- | --- | --- | --- | --- | --- |
| Badawy 2017 (7) | 6m - 5y | Egypt | Clindamycin | 22 | 62 | 35.48 | 0 | 62 | 0.00 | 40 | 62 | 64.52 | 35.48 |
|  |  |  | Doxycycline | 48 | 62 | 77.42 | 5 | 62 | 8.06 | 9 | 62 | 14.52 | 85.48 |
|  |  |  | Erythromycin | 31 | 62 | 50.00 | 0 | 62 | 0.00 | 31 | 62 | 50.00 | 50.00 |
|  |  |  | Levofloxacin | 0 | 62 | 0.00 | 0 | 62 | 0.00 | 62 | 62 | 100.00 | 0.00 |
|  |  |  | Ofloxacin | 3 | 62 | 4.84 | 14 | 62 | 22.58 | 45 | 62 | 72.58 | 27.42 |
|  |  |  | Oxacilin | 45 | 62 | 72.58 | 0 | 62 | 0.00 | 17 | 62 | 27.42 | 72.58 |
|  |  |  | Sulfamethoxazole_trimethoprim | 60 | 62 | 96.77 | 0 | 62 | 0.00 | 2 | 62 | 3.23 | 96.77 |
|  |  |  | Tetracycline | 45 | 62 | 72.58 | 7 | 62 | 11.29 | 10 | 62 | 16.13 | 83.87 |
|  |  |  | Vancomycin | 0 | 62 | 0.00 | 0 | 62 | 0.00 | 62 | 62 | 100.00 | 0.00 |
| El-Nawawy 2015 (8) | <5 y | Egypt | Amoxicilin | 0 | 100 | 0.00 | 0 | 100 | 0.00 | 100 | 100 | 100.00 | 0.00 |
|  |  |  | Cefepime | 0 | 100 | 0.00 | 0 | 100 | 0.00 | 100 | 100 | 100.00 | 0.00 |
|  |  |  | Cefotaxime | 0 | 100 | 0.00 | 1 | 100 | 1.00 | 99 | 100 | 99.00 | 1.00 |
|  |  |  | Ceftriaxone | 0 | 100 | 0.00 | 0 | 100 | 0.00 | 100 | 100 | 100.00 | 0.00 |
|  |  |  | Cefuroxime | 1 | 100 | 1.00 | 1 | 100 | 1.00 | 99 | 100 | 99.00 | 2.00 |
|  |  |  | Chloramphenicol | 0 | 100 | 0.00 | 0 | 100 | 0.00 | 100 | 100 | 100.00 | 0.00 |
|  |  |  | Clindamycin | 25 | 100 | 25.00 | 0 | 100 | 0.00 | 75 | 100 | 75.00 | 25.00 |
|  |  |  | Erythromycin | 40 | 100 | 40.00 | 3 | 100 | 3.00 | 57 | 100 | 57.00 | 43.00 |
|  |  |  | Imipenem | 0 | 100 | 0.00 | 0 | 100 | 0.00 | 100 | 100 | 100.00 | 0.00 |
|  |  |  | Linezolid | 0 | 100 | 0.00 | 0 | 100 | 0.00 | 100 | 100 | 100.00 | 0.00 |
|  |  |  | Meropenem | 0 | 100 | 0.00 | 0 | 100 | 0.00 | 100 | 100 | 100.00 | 0.00 |
|  |  |  | Penicilin | 13 | 100 | 13.00 | 2 | 100 | 2.00 | 85 | 100 | 85.00 | 15.00 |
|  |  |  | Quinolones | 1 | 100 | 1.00 | 3 | 100 | 3.00 | 96 | 100 | 96.00 | 4.00 |
|  |  |  | Rifampicin | 1 | 100 | 1.00 | 1 | 100 | 1.00 | 98 | 100 | 98.00 | 2.00 |
|  |  |  | Sulfamethoxazole_trimethoprim | 55 | 100 | 55.00 | 7 | 100 | 7.00 | 38 | 100 | 38.00 | 62.00 |
|  |  |  | Tetracycline and doxycycline | 49 | 100 | 49.00 | 6 | 100 | 6.00 | 45 | 100 | 45.00 | 55.00 |
|  |  |  | Vancomycin | 0 | 100 | 0.00 | 0 | 100 | 0.00 | 100 | 100 | 100.00 | 0.00 |
| El-Kholy 2020 (9) | <5 y | Egypt | Ampicilin | 74 | 110 | 67.27 |  |  | NA | 36 | 110 | 32.73 | 67.27 |
|  |  |  | Ceftriaxone | 50 | 110 | 45.45 |  |  | NA | 60 | 110 | 54.55 | 45.45 |
|  |  |  | Clindamycin | 30 | 110 | 27.27 |  |  | NA | 80 | 110 | 72.73 | 27.27 |
|  |  |  | Erythromycin | 49 | 110 | 44.55 |  |  | NA | 61 | 110 | 55.45 | 44.55 |
|  |  |  | Levofloxacin | 10 | 110 | 9.09 |  |  | NA | 100 | 110 | 90.91 | 9.09 |
|  |  |  | Linezolid | 0 | 110 | 0.00 |  |  | NA | 110 | 110 | 100.00 | 0.00 |
|  |  |  | Sulfamethoxazole_trimethoprim | 60 | 110 | 54.55 |  |  | NA | 50 | 110 | 45.45 | 54.55 |
|  |  |  | Tetracycline | 74 | 110 | 67.27 |  |  | NA | 36 | 110 | 32.73 | 67.27 |
|  |  |  | Vancomycin | 0 | 110 | 0.00 |  |  | NA | 110 | 110 | 100.00 | 0.00 |
| Assefa 2013 (12) | <10 | Ethiopia | Ceftriaxone | 0 | 96 | 0.00 | 0 | 96 | 0.00 | 96 | 96 | 100.00 | 0.00 |
|  |  |  | Chloramphenicol | 14 | 96 | 14.58 | 0 | 96 | 0.00 | 82 | 96 | 85.42 | 14.58 |
|  |  |  | Ciprofloxacin | 2 | 96 | 2.08 | 12 | 96 | 12.50 | 82 | 96 | 85.42 | 14.58 |
|  |  |  | Cotrimaxazole | 22 | 96 | 22.92 | 6 | 96 | 6.25 | 66 | 96 | 68.75 | 29.17 |
|  |  |  | Erythromycin | 32 | 96 | 33.33 | 0 | 96 | 0.00 | 64 | 96 | 66.67 | 33.33 |
|  |  |  | Penicilin | 10 | 96 | 10.42 | 0 | 96 | 0.00 | 86 | 96 | 89.58 | 10.42 |
|  |  |  | Tetracycline | 32 | 96 | 33.33 | 0 | 96 | 0.00 | 64 | 96 | 66.67 | 33.33 |
|  |  |  | Vancomycin | 0 | 96 | 0.00 | 0 | 96 | 0.00 | 96 | 96 | 100.00 | 0.00 |
| Gebre 2017 (14) | <5 y | Ethiopia | Chloramphenicol | 21 | 158 | 13.29 | 0 | 158 | 0.00 | 137 | 158 | 86.71 | 13.29 |
|  |  |  | Erythromycin | 9 | 158 | 5.70 | 5 | 158 | 3.16 | 144 | 158 | 91.14 | 8.86 |
|  |  |  | Pencilin | 57 | 158 | 36.08 | 0 | 158 | 0.00 | 101 | 158 | 63.92 | 36.08 |
|  |  |  | Tetracyline | 69 | 158 | 43.67 | 15 | 158 | 9.49 | 74 | 158 | 46.84 | 53.16 |
|  |  |  | Trimethoprin/sulfamethoxazole | 60 | 158 | 37.97 | 9 | 158 | 5.70 | 89 | 158 | 56.33 | 43.67 |
| Wada 2019 (15) | 3-13 y | Ethiopia | Chloramphenicol |  | 311 | 12.50 |  | 311 | 0.00 |  | 311 | 87.50 | 12.50 |
|  |  |  | Cotrimaxazole |  | 311 | 45.30 |  | 311 | 4.80 |  | 311 | 49.80 | 50.10 |
|  |  |  | Erythromycin |  | 311 | 17.00 |  | 311 | 1.90 |  | 311 | 81.00 | 18.90 |
|  |  |  | Oxacilin |  | 311 | 28.30 |  | 311 | 0.00 |  | 311 | 71.70 | 28.30 |
|  |  |  | Tetracycline |  | 311 | 48.90 |  | 311 | 2.90 |  | 311 | 48.20 | 51.80 |
| Mulu 2018 (16) | 6-16 y | Ethiopia | Chloramphenicol | 12 | 31 | 38.71 |  |  | NA |  |  | NA | 38.71 |
|  |  |  | Cotrimaxazole | 8 | 31 | 25.81 |  |  | NA |  |  | NA | 25.81 |
|  |  |  | Erythromycin | 11 | 31 | 35.48 |  |  | NA |  |  | NA | 35.48 |
|  |  |  | Penicilin | 6 | 31 | 19.35 |  |  | NA |  |  | NA | 19.35 |
|  |  |  | Tetracycline | 11 | 31 | 35.48 |  |  | NA |  |  | NA | 35.48 |
| Negash 2021 (17) | 0-15 y | Ethiopia | Chloramphenicol | 2 | 4 | 50.00 |  |  | NA |  |  | NA | 50.00 |
|  |  |  | Erythromycin | 1 | 4 | 25.00 |  |  | NA |  |  | NA | 25.00 |
|  |  |  | Penicilin | 0 | 4 | 0.00 |  |  | NA |  |  | NA | 0.00 |
|  |  |  | Tetracycline | 0 | 4 | 0.00 |  |  | NA |  |  | NA | 0.00 |
|  |  |  | Trimethoprin/sulfamethoxazole | 2 | 4 | 50.00 |  |  | NA |  |  | NA | 50.00 |
| Abaye 2013 (18) | <6 | Ethiopia | Amoxicilin | 18 | 88 | 20.45 | 2 | 88 | 2.27 | 68 | 88 | 77.27 | 22.73 |
|  |  |  | Augmentin | 1 | 88 | 1.14 | 0 | 88 | 0.00 | 87 | 88 | 98.86 | 1.14 |
|  |  |  | Chloramphenicol | 4 | 88 | 4.55 | 1 | 88 | 1.14 | 83 | 88 | 94.32 | 5.68 |
|  |  |  | Ciprofloxacin | 3 | 88 | 3.41 | 7 | 88 | 7.95 | 78 | 88 | 88.64 | 11.36 |
|  |  |  | Erythromycin | 21 | 88 | 23.86 | 0 | 88 | 0.00 | 67 | 88 | 76.14 | 23.86 |
|  |  |  | Penicilin | 13 | 88 | 14.77 | 4 | 88 | 4.55 | 70 | 88 | 79.55 | 19.32 |
|  |  |  | Vancomycin | 2 | 88 | 2.27 | 0 | 88 | 0.00 | 86 | 88 | 97.73 | 2.27 |
| Abateneh 2020 (19) | <15 | Ethiopia | Chloramphenicol | 17 | 74 | 22.97 | - | - | NA | 57 | 74 | 77.03 | 22.97 |
|  |  |  | Erythromycin | 20 | 74 | 27.03 | 16 | 74 | 21.62 | 38 | 74 | 51.35 | 48.65 |
|  |  |  | Oxacilin | - | - | NA | - | - | NA | 49 | 74 | 66.22 | NA |
|  |  |  | Tetracycline | 36 | 74 | 48.65 | 24 | 74 | 32.43 | 14 | 71 | 19.72 | 81.08 |
|  |  |  | Trimethoprin/sulfamethoxazole | 29 | 74 | 39.19 | 17 | 74 | 22.97 | 28 | 74 | 37.84 | 62.16 |
| Haile 2019 (20) | <6 y | Ethiopia | Chloramphenicol | 6 | 68 | 8.82 | - | 68 | NA | 62 | 68 | 91.18 | 8.82 |
|  |  |  | Clindamycin | 3 | 68 | 4.41 | 0 | 68 | 0.00 | 65 | 68 | 95.59 | 4.41 |
|  |  |  | Cotrimaxazole | 44 | 68 | 64.71 | 12 | 68 | 17.65 | 12 | 68 | 17.65 | 82.35 |
|  |  |  | Erythromycin | 3 | 68 | 4.41 | 7 | 68 | 10.29 | 58 | 68 | 85.29 | 14.71 |
|  |  |  | Oxacilin | 33 | 68 | 48.53 | - | 68 | NA | 35 | 68 | 51.47 | 48.53 |
|  |  |  | Rifampicin | 0 | 68 | 0.00 | 1 | 68 | 1.47 | 67 | 68 | 98.53 | 1.47 |
|  |  |  | Tetracycline | 29 | 68 | 42.65 | 16 | 68 | 23.53 | 23 | 68 | 33.82 | 66.18 |
| Seid 2020 (21) | 16-59 y | Ethiopia | Chloramphenicol | 3 | 34 | 8.82 |  |  | NA |  |  | NA | 8.82 |
|  |  |  | Cotrimaxazole | 24 | 34 | 70.59 |  |  | NA |  |  | NA | 70.59 |
|  |  |  | Erythromycin | 7 | 34 | 20.59 |  |  | NA |  |  | NA | 20.59 |
|  |  |  | peniclin | 17 | 34 | 50.00 |  |  | NA |  |  | NA | 50.00 |
|  |  |  | Tetracycline | 21 | 34 | 61.76 |  |  | NA |  |  | NA | 61.76 |
| Hussen 2020 (22) | <5 y | Ethiopia | Chloramphenicol | 17 | 161 | 10.56 | 7 | 161 | 4.35 | 137 | 161 | 85.09 | 14.91 |
|  |  |  | Erythromycin | 11 | 161 | 6.83 | 16 | 161 | 9.94 | 134 | 161 | 83.23 | 16.77 |
|  |  |  | Oxacilin | 62 | 161 | 38.51 | 13 | 161 | 8.07 | 86 | 161 | 53.42 | 46.58 |
|  |  |  | Tetracyline | 60 | 161 | 37.27 | 17 | 161 | 10.56 | 84 | 161 | 52.17 | 47.83 |
|  |  |  | Trimethoprin/sulfamethoxazole | 55 | 161 | 34.16 | 14 | 161 | 8.70 | 92 | 161 | 57.14 | 42.86 |
|  |  |  | Vancomycin | 13 | 161 | 8.07 | 0 | 161 | 0.00 | 148 | 161 | 91.93 | 8.07 |
| Nyandika 2007 (26) | < 5y | Kenya | Ampicilin | 7 | 28 | 25.00 | 7 | 28 | 25.00 | 14 | 28 | 50.00 | 50.00 |
|  |  |  | Chloramphenicol | 1 | 27 | 3.70 | 3 | 27 | 11.11 | 23 | 27 | 85.19 | 14.81 |
|  |  |  | Cotrimaxazole | 21 | 27 | 77.78 | 1 | 27 | 3.70 | 5 | 27 | 18.52 | 81.48 |
|  |  |  | Erythromycin | 0 | 27 | 0.00 | 0 | 27 | 0.00 | 27 | 27 | 100.00 | 0.00 |
|  |  |  | Lincomycin | 0 | 25 | 0.00 | 2 | 25 | 8.00 | 23 | 25 | 92.00 | 8.00 |
|  |  |  | Methicilin | 1 | 23 | 4.35 | 8 | 23 | 34.78 | 14 | 23 | 60.87 | 39.13 |
|  |  |  | Minocycline | 0 | 25 | 0.00 | 2 | 25 | 8.00 | 23 | 25 | 92.00 | 8.00 |
|  |  |  | Penicilin | 13 | 25 | 52.00 | 2 | 25 | 8.00 | 10 | 25 | 40.00 | 60.00 |
| Conklin 2016 (29) | 17-74 | Kenya | Ceftriaxone | 0 | 309 | 0.00 | 0 | 309 | 0.00 | 309 | 309 | 100.00 | 0.00 |
|  |  |  | Chloramphenicol | 4 | 309 | 1.29 | - | 309 | NA | 305 | 309 | 98.71 | 1.29 |
|  |  |  | Clindamycin | 1 | 309 | 0.32 | 0 | 309 | 0.00 | 308 | 309 | 99.68 | 0.32 |
|  |  |  | Cotrimaxazole | 291 | 309 | 94.17 | 13 | 309 | 4.21 | 3 | 309 | 0.97 | 98.38 |
|  |  |  | Erythromycin | 3 | 309 | 0.97 | 1 | 309 | 0.32 | 305 | 309 | 98.71 | 1.29 |
|  |  |  | Levofloxacin | 0 | 309 | 0.00 | 0 | 309 | 0.00 | 309 | 309 | 100.00 | 0.00 |
|  |  |  | Penicilin | 6 | 309 | 1.94 | 249 | 309 | 80.58 | 60 | 309 | 19.42 | 82.52 |
|  |  |  | Tetracycline | 64 | 309 | 20.71 | 12 | 309 | 3.88 | 232 | 309 | 75.08 | 24.60 |
| Kobayashi 2017 (31) | <5 y | Kenya | Ceftriaxone | 0 | 633 | 0.00 | 0 | 633 | 0.00 | 633 | 633 | 100.00 | 0.00 |
|  |  |  | Chloramphenicol | 12 | 627 | 1.91 | - | 627 | NA | 615 | 627 | 98.09 | 1.91 |
|  |  |  | Clindamycin | 1 | 633 | 0.16 | 0 | 633 | 0.00 | 632 | 633 | 99.84 | 0.16 |
|  |  |  | Cotrimaxazole | 561 | 631 | 88.91 | 58 | 631 | 9.19 | 12 | 631 | 1.90 | 98.10 |
|  |  |  | Erythromycin | 7 | 633 | 1.11 | 1 | 633 | 0.16 | 625 | 633 | 98.74 | 1.26 |
|  |  |  | Levofloxacin | 0 | 627 | 0.00 | 0 | 627 | 0.00 | 627 | 627 | 100.00 | 0.00 |
|  |  |  | Penicilin | 15 | 633 | 2.37 | 500 | 633 | 78.99 | 118 | 633 | 18.64 | 81.36 |
|  |  |  | Tetracycline | 107 | 633 | 16.90 | 15 | 633 | 2.37 | 511 | 633 | 80.73 | 19.27 |
| Kobayashi 2020 (32) | <5 y | Kenya | Ceftriaxone | 0 | 499 | 0.00 | 0 | 499 | 0.00 | 499 | 499 | 100.00 | 0.00 |
|  |  |  | Ceftriaxone | 0 | 445 | 0.00 | 7 | 445 | 1.57 | 438 | 445 | 98.43 | 1.57 |
|  |  |  | Ceftriaxone | 0 | 163 | 0.00 | 0 | 163 | 0.00 | 163 | 163 | 100.00 | 0.00 |
|  |  |  | Ceftriaxone | 0 | 181 | 0.00 | 0 | 181 | 0.00 | 181 | 181 | 100.00 | 0.00 |
|  |  |  | Chloramphenicol | 9 | 499 | 1.80 | - | 499 | NA | 490 | 499 | 98.20 | 1.80 |
|  |  |  | Chloramphenicol | 14 | 445 | 3.15 | - | 445 | NA | 431 | 445 | 96.85 | 3.15 |
|  |  |  | Chloramphenicol | 4 | 163 | 2.45 | - | 163 | NA | 153 | 163 | 93.87 | 2.45 |
|  |  |  | Chloramphenicol | 6 | 181 | 3.31 | - | 181 | NA | 175 | 181 | 96.69 | 3.31 |
|  |  |  | Clindamycin | 1 | 499 | 0.20 | 0 | 499 | 0.00 | 498 | 499 | 99.80 | 0.20 |
|  |  |  | Clindamycin | 12 | 445 | 2.70 | 0 | 445 | 0.00 | 433 | 445 | 97.30 | 2.70 |
|  |  |  | Clindamycin | 0 | 163 | 0.00 | 0 | 163 | 0.00 | 163 | 163 | 100.00 | 0.00 |
|  |  |  | Clindamycin | 0 | 181 | 0.00 | 0 | 181 | 0.00 | 181 | 181 | 100.00 | 0.00 |
|  |  |  | Cotrimaxazole | 430 | 499 | 86.17 | 54 | 499 | 10.82 | 15 | 499 | 3.01 | 96.99 |
|  |  |  | Cotrimaxazole | 401 | 445 | 90.11 | 27 | 445 | 6.07 | 17 | 445 | 3.82 | 96.18 |
|  |  |  | Cotrimaxazole | 152 | 163 | 93.25 | 9 | 163 | 5.52 | 0 | 163 | 0.00 | 98.77 |
|  |  |  | Cotrimaxazole | 171 | 181 | 94.48 | 10 | 181 | 5.52 | 0 | 181 | 0.00 | 100.00 |
|  |  |  | Erythromycin | 8 | 499 | 1.60 | 1 | 499 | 0.20 | 490 | 499 | 98.20 | 1.80 |
|  |  |  | Erythromycin | 17 | 445 | 3.82 | 0 | 445 | 0.00 | 428 | 445 | 96.18 | 3.82 |
|  |  |  | Erythromycin | 1 | 163 | 0.61 | 0 | 163 | 0.00 | 162 | 163 | 99.39 | 0.61 |
|  |  |  | Erythromycin | 0 | 181 | 0.00 | 0 | 181 | 0.00 | 181 | 181 | 100.00 | 0.00 |
|  |  |  | Levofloxacin | 0 | 499 | 0.00 | 0 | 499 | 0.00 | 499 | 499 | 100.00 | 0.00 |
|  |  |  | Levofloxacin | 0 | 445 | 0.00 | 0 | 445 | 0.00 | 445 | 445 | 100.00 | 0.00 |
|  |  |  | Levofloxacin | 0 | 163 | 0.00 | 0 | 163 | 0.00 | 157 | 163 | 96.32 | 0.00 |
|  |  |  | Levofloxacin | 0 | 181 | 0.00 | 0 | 181 | 0.00 | 181 | 181 | 100.00 | 0.00 |
|  |  |  | Penicilin | 12 | 499 | 2.40 | 387 | 499 | 77.56 | 100 | 499 | 20.04 | 79.96 |
|  |  |  | Penicilin | 12 | 445 | 2.70 | 333 | 445 | 74.83 | 100 | 445 | 22.47 | 77.53 |
|  |  |  | Penicilin | 3 | 163 | 1.84 | 133 | 163 | 81.60 | 27 | 163 | 16.56 | 83.44 |
|  |  |  | Penicilin | 0 | 181 | 0.00 | 150 | 181 | 82.87 | 31 | 181 | 17.13 | 82.87 |
|  |  |  | Tetracycline | 85 | 499 | 17.03 | 13 | 499 | 2.61 | 401 | 499 | 80.36 | 19.64 |
|  |  |  | Tetracycline | 57 | 445 | 12.81 | 11 | 445 | 2.47 | 377 | 445 | 84.72 | 15.28 |
|  |  |  | Tetracycline | 30 | 163 | 18.40 | 2 | 163 | 1.23 | 131 | 163 | 80.37 | 19.63 |
|  |  |  | Tetracycline | 21 | 181 | 11.60 | 17 | 181 | 9.39 | 143 | 181 | 79.01 | 20.99 |
| Githii 2013 (36) | <5 y | Kenya | Cefotaxime | 4 | 55 | 7.27 | 0 | 55 | 0.00 | 51 | 55 | 92.73 | 7.27 |
|  |  |  | Chloramphenicol | 1 | 55 | 1.82 | 0 | 55 | 0.00 | 54 | 55 | 98.18 | 1.82 |
|  |  |  | Cotrimaxazole | 54 | 55 | 98.18 | 0 | 55 | 0.00 | 1 | 55 | 1.82 | 98.18 |
|  |  |  | Erythromycin | 2 | 55 | 3.64 | 0 | 55 | 0.00 | 53 | 55 | 96.36 | 3.64 |
|  |  |  | Penicilin | 5 | 55 | 9.09 | 0 | 55 | 0.00 | 50 | 55 | 90.91 | 9.09 |
|  |  |  | Tetracycline | 0 | 55 | 0.00 | 0 | 55 | 0.00 | 55 | 55 | 100.00 | 0.00 |
| Vallès 2006 (45) | <5 y | Mozambique | Cefotaxime | 0 | 248 | 0.00 | 0 | 248 | 0.00 |  |  | NA | 0.00 |
|  |  |  | Chloramphenicol | 4 | 248 | 1.61 | 0 | 248 | 0.00 |  |  | NA | 1.61 |
|  |  |  | Cotrimaxazole | 59 | 248 | 23.79 | 91 | 248 | 36.69 |  |  | NA | 60.48 |
|  |  |  | Erythromycin | 4 | 248 | 1.61 | 0 | 248 | 0.00 |  |  | NA | 1.61 |
|  |  |  | Peniclin | 0 | 248 | 0.00 | 128 | 248 | 51.61 |  |  | NA | 51.61 |
| Valenciano 2021 (49) | <5 y | Mozambique | Amoxicilin | 0 | 246 | 0.00 |  |  | NA |  |  | NA | 0.00 |
|  |  |  | Amoxicilin | 1 | 277 | 0.36 |  |  | NA |  |  | NA | 0.36 |
|  |  |  | Amoxicilin | 0 | 221 | 0.00 |  |  | NA |  |  | NA | 0.00 |
|  |  |  | Ceftriaxone | 0 | 246 | 0.00 |  |  | NA |  |  | NA | 0.00 |
|  |  |  | Ceftriaxone | 1 | 277 | 0.36 |  |  | NA |  |  | NA | 0.36 |
|  |  |  | Ceftriaxone | 0 | 221 | 0.00 |  |  | NA |  |  | NA | 0.00 |
|  |  |  | Cefuroxime | 0 | 246 | 0.00 |  |  | NA |  |  | NA | 0.00 |
|  |  |  | Cefuroxime | 1 | 277 | 0.36 |  |  | NA |  |  | NA | 0.36 |
|  |  |  | Cefuroxime | 1 | 221 | 0.45 |  |  | NA |  |  | NA | 0.45 |
|  |  |  | Chloramphenicol | 13 | 246 | 5.28 |  |  | NA |  |  | NA | 5.28 |
|  |  |  | Chloramphenicol | 1 | 277 | 0.36 |  |  | NA |  |  | NA | 0.36 |
|  |  |  | Chloramphenicol | 9 | 221 | 4.07 |  |  | NA |  |  | NA | 4.07 |
|  |  |  | Clindamycin | 0 | 246 | 0.00 |  |  | NA |  |  | NA | 0.00 |
|  |  |  | Clindamycin | 2 | 277 | 0.72 |  |  | NA |  |  | NA | 0.72 |
|  |  |  | Clindamycin | 5 | 221 | 2.26 |  |  | NA |  |  | NA | 2.26 |
|  |  |  | Erythromycin | 34 | 246 | 13.82 |  |  | NA |  |  | NA | 13.82 |
|  |  |  | Erythromycin | 20 | 277 | 7.22 |  |  | NA |  |  | NA | 7.22 |
|  |  |  | Erythromycin | 22 | 221 | 9.95 |  |  | NA |  |  | NA | 9.95 |
|  |  |  | Levofloxacin | 0 | 246 | 0.00 |  |  | NA |  |  | NA | 0.00 |
|  |  |  | Levofloxacin | 0 | 277 | 0.00 |  |  | NA |  |  | NA | 0.00 |
|  |  |  | Levofloxacin | 0 | 221 | 0.00 |  |  | NA |  |  | NA | 0.00 |
|  |  |  | Meropenem | 1 | 246 | 0.41 |  |  | NA |  |  | NA | 0.41 |
|  |  |  | Meropenem | 2 | 277 | 0.72 |  |  | NA |  |  | NA | 0.72 |
|  |  |  | Meropenem | 0 | 221 | 0.00 |  |  | NA |  |  | NA | 0.00 |
|  |  |  | Peniclin | 123 | 246 | 50.00 |  |  | NA |  |  | NA | 50.00 |
|  |  |  | Peniclin | 133 | 277 | 48.01 |  |  | NA |  |  | NA | 48.01 |
|  |  |  | Peniclin | 102 | 221 | 46.15 |  |  | NA |  |  | NA | 46.15 |
|  |  |  | Rifampicin | 2 | 246 | 0.81 |  |  | NA |  |  | NA | 0.81 |
|  |  |  | Rifampicin | 0 | 277 | 0.00 |  |  | NA |  |  | NA | 0.00 |
|  |  |  | Rifampicin | 0 | 221 | 0.00 |  |  | NA |  |  | NA | 0.00 |
|  |  |  | Synercid | 0 | 246 | 0.00 |  |  | NA |  |  | NA | 0.00 |
|  |  |  | Synercid | 0 | 277 | 0.00 |  |  | NA |  |  | NA | 0.00 |
|  |  |  | Synercid | 1 | 221 | 0.45 |  |  | NA |  |  | NA | 0.45 |
|  |  |  | Tetracycline | 57 | 246 | 23.17 |  |  | NA |  |  | NA | 23.17 |
|  |  |  | Tetracycline | 40 | 277 | 14.44 |  |  | NA |  |  | NA | 14.44 |
|  |  |  | Tetracycline | 48 | 221 | 21.72 |  |  | NA |  |  | NA | 21.72 |
|  |  |  | Trimethoprin/sulfamethoxazole | 226 | 246 | 91.87 |  |  | NA |  |  | NA | 91.87 |
|  |  |  | Trimethoprin/sulfamethoxazole | 260 | 277 | 93.86 |  |  | NA |  |  | NA | 93.86 |
|  |  |  | Trimethoprin/sulfamethoxazole | 194 | 221 | 87.78 |  |  | NA |  |  | NA | 87.78 |
|  |  |  | Vancomycin | 0 | 246 | 0.00 |  |  | NA |  |  | NA | 0.00 |
|  |  |  | Vancomycin | 0 | 277 | 0.00 |  |  | NA |  |  | NA | 0.00 |
|  |  |  | Vancomycin | 0 | 221 | 0.00 |  |  | NA |  |  | NA | 0.00 |
| Verani 2018 (50) | <5 y | Mozambique | Amoxicilin | 1 | 343 | 0.29 | 3 | 343 | 0.87 | 339 | 343 | 98.83 | 1.17 |
|  |  |  | Ceftriaxone | 0 | 343 | 0.00 | 0 | 343 | 0.00 | 343 | 343 | 100.00 | 0.00 |
|  |  |  | Chloramphenicol | 23 | 343 | 6.71 | 0 | 343 | 0.00 | 320 | 343 | 93.29 | 6.71 |
|  |  |  | Erythromycin | 56 | 343 | 16.33 | 1 | 343 | 0.29 | 286 | 343 | 83.38 | 16.62 |
|  |  |  | Penicilin oral | 3 | 343 | 0.87 | 224 | 343 | 65.31 | 116 | 343 | 33.82 | 66.18 |
|  |  |  | Penicilin parenteral | 0 | 343 | 0.00 | 3 | 343 | 0.87 | 340 | 343 | 99.13 | 0.87 |
|  |  |  | Rifampicin | 2 | 343 | 0.58 | 0 | 343 | 0.00 | 341 | 343 | 99.42 | 0.58 |
|  |  |  | Trimethoprin/sulfamethoxazole | 304 | 343 | 88.63 | 24 | 343 | 7.00 | 15 | 343 | 4.37 | 95.63 |
| Saeed 2011 (57) | <1 y | South Sudan | Cefachlor | 0 | 14 | 0.00 |  |  | NA | 14 | 14 | 100.00 | 0.00 |
|  |  |  | Chloramphenicol | 0 | 14 | 0.00 |  |  | NA | 14 | 14 | 100.00 | 0.00 |
|  |  |  | Cotrimaxazole | 14 | 14 | 100.00 |  |  | NA | 0 | 14 | 0.00 | 100.00 |
|  |  |  | Erythromycin | 0 | 14 | 0.00 |  |  | NA | 14 | 14 | 100.00 | 0.00 |
|  |  |  | Linezolid | 0 | 14 | 0.00 |  |  | NA | 14 | 14 | 100.00 | 0.00 |
|  |  |  | Penicilin | 14 | 14 | 100.00 |  |  | NA | 0 | 14 | 0.00 | 100.00 |
|  |  |  | Tetracycline | 4 | 14 | 28.57 |  |  | NA | 10 | 14 | 71.43 | 28.57 |
| Batt 2003 (58) | ≤7 | Tanzania | Cotrimaxazole | 39 | 141 | 27.66 | 20 | 141 | 14.18 | 82 | 141 | 58.16 | 41.84 |
|  |  |  | Penicilin | 0 | 141 | 0.00 | 30 | 141 | 21.28 | 111 | 141 | 78.72 | 21.28 |
| Moyo 2012 (61) | <5 y | Tanzania | Ceftriaxone | 0 | 115 | 0.00 |  |  | NA |  |  | NA | 0.00 |
|  |  |  | Chloramphenicol | 4 | 115 | 3.48 |  |  | NA |  |  | NA | 3.48 |
|  |  |  | Clindamycin | 0 | 115 | 0.00 |  |  | NA |  |  | NA | 0.00 |
|  |  |  | Erythromycin | 7 | 115 | 6.09 |  |  | NA |  |  | NA | 6.09 |
|  |  |  | MDR | 19 | 115 | 16.52 |  |  | NA |  |  | NA | 16.52 |
|  |  |  | Peniclin | 1 | 115 | 0.87 | 77 | 115 | 66.96 | 37 | 115 | 32.17 | 67.83 |
|  |  |  | Tetracycline | 12 | 115 | 10.43 |  |  | NA |  |  | NA | 10.43 |
|  |  |  | Trimethoprin/sulfamethoxazole | 95 | 115 | 82.61 |  |  | NA |  |  | NA | 82.61 |
| Ndossa 2015 (62) | 2-5 y | Tanzania | Amoxicilin/clavulanic acid | 3 | 43 | 6.98 |  |  | NA | 40 | 43 | 93.02 | #DIV/0! |
|  |  |  | Ciprofloxacin | 0 | 43 | 0.00 |  |  | NA | 43 | 43 | 100.00 | #DIV/0! |
|  |  |  | Cotrimaxazole | 38 | 43 | 88.37 |  |  | NA | 5 | 43 | 11.63 | #DIV/0! |
|  |  |  | Erythromycin | 18 | 43 | 41.86 |  |  | NA | 25 | 43 | 58.14 | #DIV/0! |
|  |  |  | gentamycin | 8 | 43 | 18.60 |  |  | NA | 35 | 43 | 81.40 | #DIV/0! |
|  |  |  | Penicilin | 17 | 43 | 39.53 |  |  | NA | 26 | 43 | 60.47 | #DIV/0! |
| Emgård 2019 (63) | <2 y | Tanzania | Ampicilin | 0 | 244 | 0.00 | 8 | 244 | 3.28 |  |  |  | 3.28 |
|  |  |  | Ceftriaxone | 0 | 244 | 0.00 | 9 | 244 | 3.69 |  |  |  | 3.69 |
|  |  |  | Clindamycin | 14 | 244 | 5.74 | - | 244 | NA |  |  |  | 5.74 |
|  |  |  | Erythromycin | 21 | 244 | 8.61 | 15 | 244 | 6.15 |  |  |  | 14.75 |
|  |  |  | Norfloxacin | 0 | 244 | 0.00 | - | 244 | NA |  |  |  | 0.00 |
|  |  |  | Pencilin G | 0 | 244 | 0.00 | 98 | 244 | 40.16 |  |  |  | 40.16 |
|  |  |  | Penicilin V | 112 | 244 | 45.90 | 0 | 244 | 0.00 |  |  |  | 45.90 |
|  |  |  | Tetracyline | 54 | 244 | 22.13 | 20 | 244 | 8.20 |  |  |  | 30.33 |
|  |  |  | Trimethoprin/sulfamethoxazole | 225 | 244 | 92.21 | 11 | 244 | 4.51 |  |  |  | 96.72 |
| Bloch 2017 (64) | <5 y | Tanzania | Azithromycin | 65 | 454 | 14.32 | 36 | 454 | 7.93 | 353 | 454 | 77.75 | 22.25 |
| Manyahi 2020 (68) | adults | Tanzania | Azithromycin | 17 | 76 | 22.37 |  |  | NA |  |  | NA | 22.37 |
|  |  |  | Chloramphenicol | 14 | 76 | 18.42 |  |  | NA |  |  | NA | 18.42 |
|  |  |  | Clindamycin | 8 | 76 | 10.53 |  |  | NA |  |  | NA | 10.53 |
|  |  |  | Cotrimaxazole | 54 | 76 | 71.05 |  |  | NA |  |  | NA | 71.05 |
|  |  |  | Erythromycin | 16 | 76 | 21.05 |  |  | NA |  |  | NA | 21.05 |
|  |  |  | Levofloxacin | 0 | 76 | 0.00 |  |  | NA |  |  | NA | 0.00 |
|  |  |  | Penicilin | 56 | 76 | 73.68 |  |  | NA |  |  | NA | 73.68 |
|  |  |  | Tetracycline | 11 | 76 | 14.47 |  |  | NA |  |  | NA | 14.47 |
| Kateete 2012 (69) | <6 y | Uganda | Ceftriaxone | 0 | 27 | 0.00 | 0 | 27 | 0.00 | 27 | 27 | 100.00 | 0.00 |
|  |  |  | Ceftriaxone | 0 | 23 | 0.00 | 0 | 23 | 0.00 | 23 | 23 | 100.00 | 0.00 |
|  |  |  | Chloramphenicol | 1 | 27 | 3.70 | 0 | 27 | 0.00 | 26 | 27 | 96.30 | 3.70 |
|  |  |  | Chloramphenicol | 5 | 23 | 21.74 | 2 | 23 | 8.70 | 16 | 23 | 69.57 | 30.43 |
|  |  |  | Erythromycin | 1 | 27 | 3.70 | 0 | 27 | 0.00 | 26 | 27 | 96.30 | 3.70 |
|  |  |  | Erythromycin | 2 | 23 | 8.70 | 2 | 23 | 8.70 | 19 | 23 | 82.61 | 17.39 |
|  |  |  | Penicilin | 27 | 27 | 100.00 | 0 | 27 | 0.00 | 0 | 27 | 0.00 | 100.00 |
|  |  |  | Penicilin | 0 | 38 | 0.00 | 38 | 38 | 100.00 | 0 | 38 | 0.00 | 100.00 |
|  |  |  | Perfloxacin | 0 | 27 | 0.00 | 0 | 27 | 0.00 | 27 | 27 | 100.00 | 0.00 |
|  |  |  | Perfloxacin | 0 | 23 | 0.00 | 0 | 23 | 0.00 | 23 | 23 | 100.00 | 0.00 |
|  |  |  | Rifampicin | 0 | 27 | 0.00 | 0 | 27 | 0.00 | 27 | 27 | 100.00 | 0.00 |
|  |  |  | Rifampicin | 0 | 23 | 0.00 | 0 | 23 | 0.00 | 23 | 23 | 100.00 | 0.00 |
|  |  |  | Trimethoprin/sulfamethoxazole | 26 | 27 | 96.30 | 1 | 27 | 3.70 | 0 | 27 | 0.00 | 100.00 |
|  |  |  | Trimethoprin/sulfamethoxazole | 18 | 23 | 78.26 | 5 | 23 | 21.74 | 0 | 23 | 0.00 | 100.00 |
| Blossom 2006 (70) | 22-55 y | Uganda | Amoxicilin | 0 | 109 | 0.00 | 0 | 109 | 0.00 | 109 | 109 | 100.00 | 0.00 |
|  |  |  | Azithromycin | 1 | 109 | 0.92 | 0 | 109 | 0.00 | 108 | 109 | 99.08 | 0.92 |
|  |  |  | Cefidiner | 1 | 109 | 0.92 | 1 | 109 | 0.92 | 107 | 109 | 98.17 | 1.83 |
|  |  |  | Cefpodoxime | 0 | 109 | 0.00 | 1 | 109 | 0.92 | 108 | 109 | 99.08 | 0.92 |
|  |  |  | Ceftriaxone | 0 | 109 | 0.00 | 0 | 109 | 0.00 | 109 | 109 | 100.00 | 0.00 |
|  |  |  | Cefuroxime oral | 0 | 109 | 0.00 | 1 | 109 | 0.92 | 108 | 109 | 99.08 | 0.92 |
|  |  |  | Cefuroxime parenteral | 1 | 109 | 0.92 | 4 | 109 | 3.67 | 104 | 109 | 95.41 | 4.59 |
|  |  |  | Clindamycin | 0 | 109 | 0.00 | 0 | 109 | 0.00 | 109 | 109 | 100.00 | 0.00 |
|  |  |  | Levofloxacin | 0 | 109 | 0.00 | 0 | 109 | 0.00 | 109 | 109 | 100.00 | 0.00 |
|  |  |  | Penicilin | 0 | 109 | 0.00 | 78 | 109 | 71.56 | 31 | 109 | 28.44 | 71.56 |
|  |  |  | Trimethoprin/sulfamethoxazole | 108 | 109 | 99.08 | - |  | NA | 1 | 109 | 0.92 | 99.08 |
| Rutebemberwa 2015 (73) | <5 y | Uganda | Erythromycin | 0 | 89 | 0.00 | - | 89 | NA | 89 | 89 | 100.00 | 0.00 |
|  |  |  | Oxacilin | NA | 89 | NA | NA | 89 | NA | 17 | 89 | 19.10 | NA |
|  |  |  | Penicilin | 0 | 89 | 0.00 | 72 | 89 | 80.90 | 17 | 89 | 19.10 | 80.90 |
|  |  |  | Trimethoprin/sulfamethoxazole | 88 | 89 | 98.88 | 0 | 89 | 0.00 | 1 | 89 | 1.12 | 98.88 |
| Ndip 2008 (75) | 10-21 y | Cameroon | Ampicilin | 18 | 30 | 60.00 |  |  | NA | 12 | 30 | 40.00 | #DIV/0! |
|  |  |  | Cefazolin | 9 | 30 | 30.00 |  |  | NA | 21 | 30 | 70.00 | #DIV/0! |
|  |  |  | Cefuroxime | 5 | 30 | 16.67 |  |  | NA | 25 | 30 | 83.33 | #DIV/0! |
|  |  |  | Chloramphenicol | 14 | 30 | 46.67 |  |  | NA | 16 | 30 | 53.33 | #DIV/0! |
|  |  |  | Cotrimaxazole | 21 | 30 | 70.00 |  |  | NA | 9 | 30 | 30.00 | #DIV/0! |
|  |  |  | Erythromycin | 9 | 30 | 30.00 |  |  | NA | 21 | 30 | 70.00 | #DIV/0! |
|  |  |  | gentamycin | 5 | 30 | 16.67 |  |  | NA | 25 | 30 | 83.33 | #DIV/0! |
|  |  |  | Penicilin | 30 | 30 | 100.00 |  |  | NA | 0 | 30 | 0.00 | #DIV/0! |
| Uddén 2020 (79) | 4-12 y | Angola | Amoxicilin | 2 | 325 | 0.62 | 1 | 325 | 0.31 | 322 | 325 | 99.08 | 0.92 |
|  |  |  | Clindamycin | 2 | 325 | 0.62 | - | 325 | NA | 323 | 325 | 99.38 | 0.62 |
|  |  |  | Erythromycin | 2 | 325 | 0.62 | 1 | 325 | 0.31 | 322 | 325 | 99.08 | 0.92 |
|  |  |  | Norfloxacin | 1 | 325 | 0.31 |  | 325 | 0.00 | 324 | 325 | 99.69 | 0.31 |
|  |  |  | Penicilin G | 0 | 325 | 0.00 | 130 | 325 | 40.00 | 195 | 325 | 60.00 | 40.00 |
|  |  |  | Tetracyline | 56 | 325 | 17.23 | 12 | 325 | 3.69 | 257 | 325 | 79.08 | 20.92 |
|  |  |  | Trimethoprin/sulfamethoxazole | 262 | 325 | 80.62 | 6 | 325 | 1.85 | 57 | 325 | 17.54 | 82.46 |
| Bere 2009 (80) | <5 y | Burkina Faso | Amikacin |  | 232 | 95.19 |  |  | NA |  |  | NA | 95.19 |
|  |  |  | Amoxicilin |  | 232 | 25.00 |  |  | NA |  |  | NA | 25.00 |
|  |  |  | Amoxicilin/clavulanic acid |  | 232 | 25.00 |  |  | NA |  |  | NA | 25.00 |
|  |  |  | Ampicilin |  | 232 | 26.81 |  |  | NA |  |  | NA | 26.81 |
|  |  |  | Ceftriaxone |  | 232 | 6.74 |  |  | NA |  |  | NA | 6.74 |
|  |  |  | Chloramphenicol |  | 232 | 8.70 |  |  | NA |  |  | NA | 8.70 |
|  |  |  | Ciprofloxacin |  | 232 | 12.22 |  |  | NA |  |  | NA | 12.22 |
|  |  |  | Cotrimaxazole |  | 232 | 31.70 |  |  | NA |  |  | NA | 31.70 |
|  |  |  | Erythromycin |  | 232 | 6.10 |  |  | NA |  |  | NA | 6.10 |
|  |  |  | Lincomycin |  | 232 | 17.30 |  |  | NA |  |  | NA | 17.30 |
|  |  |  | Oxacilin |  | 232 | 3.85 |  |  | NA |  |  | NA | 3.85 |
|  |  |  | Pefloxacin |  | 232 | 54.80 |  |  | NA |  |  | NA | 54.80 |
|  |  |  | Rifampicin |  | 232 | 1.28 |  |  | NA |  |  | NA | 1.28 |
|  |  |  | Spectinomycin |  | 232 | 7.70 |  |  | NA |  |  | NA | 7.70 |
|  |  |  | Tetracycline |  | 232 | 65.30 |  |  | NA |  |  | NA | 65.30 |
|  |  |  | Vancomycin |  | 232 | 12.33 |  |  | NA |  |  | NA | 12.33 |
| Hema-Ouangraoua 2019 (81) | <5 y | Burkina Faso | Oxacilin | 37 | 147 | 25.17 |  |  | NA |  |  | NA | 25.17 |
|  |  |  | Oxacilin | 45 | 109 | 41.28 |  |  | NA |  |  | NA | 41.28 |
|  |  |  | Penicilin | 19 | 147 | 12.93 |  |  | NA |  |  | NA | 12.93 |
| Bonko 2021 (82) | <5 y | Burkina Faso | Ampicilin | 0 | 4 | 0.00 |  |  | NA |  |  | NA | 0.00 |
|  |  |  | Ampicilin | 0 | 2 | 0.00 |  |  | NA |  |  | NA | 0.00 |
|  |  |  | Ceftriaxone | 0 | 4 | 0.00 |  |  | NA |  |  | NA | 0.00 |
|  |  |  | Ceftriaxone | 0 | 2 | 0.00 |  |  | NA |  |  | NA | 0.00 |
|  |  |  | Clindamycin | 0 | 4 | 0.00 |  |  | NA |  |  | NA | 0.00 |
|  |  |  | Clindamycin | 0 | 2 | 0.00 |  |  | NA |  |  | NA | 0.00 |
|  |  |  | Clindamycin | 0 | 4 | 0.00 |  |  | NA |  |  | NA | 0.00 |
|  |  |  | Clindamycin | 1 | 2 | 50.00 |  |  | NA |  |  | NA | 50.00 |
|  |  |  | Erythromycin | 0 | 4 | 0.00 |  |  | NA |  |  | NA | 0.00 |
|  |  |  | Erythromycin | 0 | 2 | 0.00 |  |  | NA |  |  | NA | 0.00 |
|  |  |  | Imipenem | 0 | 4 | 0.00 |  |  | NA |  |  | NA | 0.00 |
|  |  |  | Imipenem | 0 | 2 | 0.00 |  |  | NA |  |  | NA | 0.00 |
|  |  |  | Penicilin | 2 | 4 | 50.00 |  |  | NA |  |  | NA | 50.00 |
|  |  |  | Penicilin | 1 | 2 | 50.00 |  |  | NA |  |  | NA | 50.00 |
|  |  |  | Tetracycline | 4 | 4 | 100.00 |  |  | NA |  |  | NA | 100.00 |
|  |  |  | Tetracycline | 2 | 2 | 100.00 |  |  | NA |  |  | NA | 100.00 |
|  |  |  | Trimethoprin/sulfamethoxazole | 3 | 4 | 75.00 |  |  | NA |  |  | NA | 75.00 |
|  |  |  | Trimethoprin/sulfamethoxazole | 2 | 2 | 100.00 |  |  | NA |  |  | NA | 100.00 |
|  |  |  | Vancomycin | 0 | 4 | 0.00 |  |  | NA |  |  | NA | 0.00 |
|  |  |  | Vancomycin | 0 | 2 | 0.00 |  |  | NA |  |  | NA | 0.00 |
| Coulibaly 2020 (85) | <5 y | Burkina Faso | Azithromycin | 4 | 32 | 12.50 |  |  | NA |  |  | NA | 12.50 |
|  |  |  | Azithromycin | 6 | 69 | 8.70 |  |  | NA |  |  | NA | 8.70 |
|  |  |  | Clindamycin | 1 | 32 | 3.13 |  |  | NA |  |  | NA | 3.13 |
|  |  |  | Clindamycin | 4 | 69 | 5.80 |  |  | NA |  |  | NA | 5.80 |
|  |  |  | Oxacilin | 10 | 29 | 34.48 |  |  | NA |  |  | NA | 34.48 |
|  |  |  | Oxacilin | 14 | 61 | 22.95 |  |  | NA |  |  | NA | 22.95 |
|  |  |  | Tetracycline | 26 | 32 | 81.25 |  |  | NA |  |  | NA | 81.25 |
|  |  |  | Tetracycline | 44 | 69 | 63.77 |  |  | NA |  |  | NA | 63.77 |
|  |  |  | Trimethoprin/sulfamethoxazole | 23 | 29 | 79.31 |  |  | NA |  |  | NA | 79.31 |
|  |  |  | Trimethoprin/sulfamethoxazole | 45 | 61 | 73.77 |  |  | NA |  |  | NA | 73.77 |
| Dayie 2013 (89) | <5 y | Ghana | Penicilin | 0 | 153 | 0.00 | 67 | 153 | 43.79 |  |  | NA | 43.79 |
|  |  |  | Penicilin | 2 | 135 | 1.48 | 63 | 135 | 46.67 |  |  | NA | 48.15 |
| Dayie 2018 (90) | Children <13y | Ghana | Cotrimaxazole | 60 | 79 | 75.90 |  |  | NA |  |  | NA | 75.90 |
|  | Children <13y |  | Erythromycin | 12 | 79 | 15.20 |  |  | NA |  |  | NA | 15.20 |
|  | Children <13y |  | Levofloxacin | 2 | 79 | 2.50 |  |  | NA |  |  | NA | 2.50 |
|  | Children <13y |  | Penicilin | 29 | 79 | 36.70 |  |  | NA |  |  | NA | 36.70 |
|  | Children <13y |  | Tetracycline | 56 | 79 | 70.90 |  |  | NA |  |  | NA | 70.90 |
|  | adults |  | Penicillin | 8 | 20 | 40.00 |  |  | NA |  |  | NA | 40.00 |
|  | adults |  | Levofloxacin | 2 | 20 | 10.00 |  |  | NA |  |  | NA | 10.00 |
|  | adults |  | Erythromycin | 2 | 20 | 10.00 |  |  | NA |  |  | NA | 10.00 |
|  | adults |  | tetracylcin | 16 | 20 | 80.00 |  |  | NA |  |  | NA | 80.00 |
|  | adults |  | Cotrimaxazole | 17 | 20 | 85.00 |  |  | NA |  |  | NA | 85.00 |
| Dayie 2019a (91) | <5 y | Ghana | Erythromycin | 24 | 220 | 10.91 | 11 | 220 | 5.00 |  |  | NA | 15.91 |
|  |  |  | Penicilin | 1 | 220 | 0.45 | 49 | 220 | 22.27 |  |  | NA | 22.73 |
|  |  |  | Tetracycline | 138 | 219 | 63.01 | 2 | 219 | 0.91 |  |  | NA | 63.93 |
|  |  |  | Trimethoprin/sulfamethoxazole | 135 | 220 | 61.36 | 32 | 220 | 14.55 |  |  | NA | 75.91 |
| Mills 2020 (93) | <5 y | Ghana | Ceftriaxone |  |  | NA |  |  | NA |  |  | NA | 2.60 |
|  |  |  | Chloramphenicol |  |  | NA |  |  | NA |  |  | NA | 11.30 |
|  |  |  | Clindamycin |  |  | NA |  |  | NA |  |  | NA | 5.30 |
|  |  |  | Erythromycin |  |  | NA |  |  | NA |  |  | NA | 7.30 |
|  |  |  | Levofloxacin | 0 | 151 | 0.00 |  |  | NA | 151 | 151 | 100.00 | 0.00 |
|  |  |  | Linezolid | 0 | 151 | 0.00 |  |  | NA | 151 | 151 | 100.00 | 0.00 |
|  |  |  | Penicilin | 1 | 151 | 0.66 |  | 151 | 35.00 |  | 151 | NA | 35.66 |
|  |  |  | Vancomycin | 0 | 151 | 0.00 |  |  | NA | 151 | 151 | 100.00 | 0.00 |
| Ousmane 2017 (97) | 3-26 m | Niger | Chloramphenicol | 19 | 211 | 9.00 | 5 | 211 | 2.37 | 187 | 211 | 88.63 | 11.37 |
|  |  |  | Clindamycin | 4 | 211 | 1.90 | 0 | 211 | 0.00 | 207 | 211 | 98.10 | 1.90 |
|  |  |  | Erythromycin | 9 | 211 | 4.27 | 0 | 211 | 0.00 | 202 | 211 | 95.73 | 4.27 |
|  |  |  | gentamycin | 0 | 211 | 0.00 | 0 | 211 | 0.00 | 211 | 211 | 100.00 | 0.00 |
|  |  |  | Kanamycin | 0 | 211 | 0.00 | 0 | 211 | 0.00 | 211 | 211 | 100.00 | 0.00 |
|  |  |  | Levofloxacin | 0 | 211 | 0.00 | 0 | 211 | 0.00 | 211 | 211 | 100.00 | 0.00 |
|  |  |  | Norfloxacin | 0 | 211 | 0.00 | 0 | 211 | 0.00 | 211 | 211 | 100.00 | 0.00 |
|  |  |  | Penicilin | 3 | 211 | 1.42 | 20 | 211 | 9.48 | 188 | 211 | 89.10 | 10.90 |
|  |  |  | Pristinamycin | 0 | 211 | 0.00 | 0 | 211 | 0.00 | 211 | 211 | 100.00 | 0.00 |
|  |  |  | Tetracycline | 148 | 210 | 70.48 | 7 | 210 | 3.33 | 55 | 210 | 26.19 | 73.81 |
|  |  |  | Vancomycin | 0 | 211 | 0.00 | 0 | 211 | 0.00 | 211 | 211 | 100.00 | 0.00 |
| Nweze 2012 (102) | 11-23 y | Nigeria | Ampicilin | 11 | 19 | 57.89 |  |  | NA |  |  | NA | 57.89 |
|  |  |  | Ceftriaxone | 1 | 19 | 5.26 |  |  | NA |  |  | NA | 5.26 |
|  |  |  | Cefuroxime | 3 | 19 | 15.79 |  |  | NA |  |  | NA | 15.79 |
|  |  |  | Chloramphenicol | 8 | 19 | 42.11 |  |  | NA |  |  | NA | 42.11 |
|  |  |  | Ciprofloxacin | 3 | 19 | 15.79 |  |  | NA |  |  | NA | 15.79 |
|  |  |  | Cotrimaxazole | 12 | 19 | 63.16 |  |  | NA |  |  | NA | 63.16 |
|  |  |  | Erythromycin | 6 | 19 | 31.58 |  |  | NA |  |  | NA | 31.58 |
|  |  |  | gentamycin | 2 | 19 | 10.53 |  |  | NA |  |  | NA | 10.53 |
|  |  |  | Penicilin | 17 | 19 | 89.47 |  |  | NA |  |  | NA | 89.47 |
|  |  |  | Tetracycline | 10 | 19 | 52.63 |  |  | NA |  |  | NA | 52.63 |
| Echave 2003 (104) | <5 y | Senegal | Chloramphenicol | 0 | 17 | 0.00 |  |  | NA |  |  | NA | 0.00 |
|  |  |  | Chloramphenicol | 6 | 47 | 12.77 |  |  | NA |  |  | NA | 12.77 |
|  |  |  | Cotrimaxazole | 7 | 17 | 41.18 |  |  | NA |  |  | NA | 41.18 |
|  |  |  | Cotrimaxazole | 13 | 47 | 27.66 |  |  | NA |  |  | NA | 27.66 |
|  |  |  | Pen or amp | 3 | 17 | 17.65 |  |  | NA |  |  | NA | 17.65 |
|  |  |  | Pen or amp | 6 | 47 | 12.77 |  |  | NA |  |  | NA | 12.77 |
| Bojang 2018 (116) | 11-13 m | The Gambia | Azithromycin | 2 | 235 | 0.85 | - | - | NA | 233 | 235 | 99.15 | 0.85 |
|  |  |  | Chloramphenicol | 3 | 235 | 1.28 |  |  | NA | 232 | 235 | 98.72 | 1.28 |
|  |  |  | Clindamycin | 0 | 235 | 0.00 |  |  | NA | 235 | 235 | 100.00 | 0.00 |
|  |  |  | Erythromycin | 3 | 235 | 1.28 | - | - | NA | 232 | 235 | 98.72 | 1.28 |
|  |  |  | Penicilin | 0 | 235 | 0.00 | - | - | NA | 235 | 235 | 100.00 | 0.00 |
| Pemba 2008 (121) | > 18 | South Africa | Cotrimaxazole | 11 | 49 | 22.45 |  |  | NA |  |  | NA | 22.45 |
|  |  |  | Penicilin | 4 | 49 | 8.16 |  |  | NA |  |  | NA | 8.16 |
| Skosana 2021 (134) | < 5 y | South Africa | Cefotaxime | 0 | 36 | 0.00 |  |  | NA | 36 | 36 | 100.00 | 0.00 |
|  |  |  | Clindamycin | 3 | 121 | 2.48 |  |  | NA | 118 | 121 | 97.52 | 2.48 |
|  |  |  | Cotrimaxazole | 54 | 121 | 44.63 |  |  | NA | 67 | 121 | 55.37 | 44.63 |
|  |  |  | Erythromycin | 8 | 121 | 6.61 |  |  | NA | 113 | 121 | 93.39 | 6.61 |
|  |  |  | Penicilin | 22 | 36 | 61.11 |  |  | NA | 14 | 36 | 38.89 | 61.11 |
|  |  |  | tetracycline | 3 | 121 | 2.48 |  |  | NA | 118 | 121 | 97.52 | 2.48 |

References

1. Warda K, Oufdou K, Zahlane K, Bouskraoui M. Antibiotic resistance and serotype distribution of nasopharyngeal isolates of Streptococcus pneumoniae from children in Marrakech region (Morocco). Journal of infection and public health. 2013 Dec;6(6):473–81.

2. Jroundi I, Mahraoui C, Benmessaoud R, Moraleda C, Munoz Almagro C, Seffar M, et al. Streptococcus pneumoniae carriage among healthy and sick pediatric patients before the generalized implementation of the 13-valent pneumococcal vaccine in Morocco from 2010 to 2011. Journal of infection and public health. 2017;10(2):165–70.

3. Dilagui I, Moussair FZ, Loqman S, Diawara I, Zerouali K, Belabbes H, et al. Streptococcus pneumoniae carriage among febrile children at the time of PCV-10 immunization in pediatric emergencies at Mohammed VI University Hospital Centre in Marrakesh (Morocco). Archives de pediatrie : organe officiel de la Societe francaise de pediatrie. 2019 Nov;26(8):453–8.

4. Brini I, Guerrero A, Hannachi N, Bouguila J, Orth-Höller D, Bouhlel A, et al. Epidemiology and clinical profile of pathogens responsible for the hospitalization of children in Sousse area, Tunisia. PloS one. 2017;12(11):e0188325.

5. Brini Khalifa I, Hannachi N, Guerrero A, Orth-Höller D, Bhiri S, Bougila J, et al. Demographic and seasonal characteristics of respiratory pathogens in neonates and infants aged 0 to 12 months in the Central-East region of Tunisia. Journal of medical virology. 2019 Apr;91(4):570–81.

6. Saafan ME, Ibrahim WS. Role of bacterial biofilms in idiopathic childhood epistaxis. European archives of oto-rhino-laryngology : official journal of the European Federation of Oto-Rhino-Laryngological Societies (EUFOS) : affiliated with the German Society for Oto-Rhino-Laryngology - Head and Neck Surgery. 2013 Mar;270(3):909–14.

7. Badawy M, El Kholy A, Sherif MM, Rahman EA, Ashour E, Sherif H, et al. Serotypes of Streptococcus pneumoniae in Egyptian children: are they covered by pneumococcal conjugate vaccines? European journal of clinical microbiology & infectious diseases : official publication of the European Society of Clinical Microbiology. 2017 Dec;36(12):2385–9.

8. El-Nawawy AA, Hafez SF, Meheissen MA, Shahtout NM, Mohammed EE. Nasopharyngeal carriage, capsular and molecular serotyping and antimicrobial susceptibility of streptococcus pneumoniae among asymptomatic healthy children in egypt. Journal of tropical pediatrics. 2015 Dec;61(6):455–63.

9. El-Kholy A, Badawy M, Gad M, Soliman M. Serotypes and antimicrobial susceptibility of nasopharyngeal isolates of streptococcus pneumoniae from children less than 5 years old in egypt. Infection and drug resistance. 2020;13:3669–77.

10. Haug S, Lakew T, Habtemariam G, Alemayehu W, Cevallos V, Zhou Z, et al. The decline of pneumococcal resistance after cessation of mass antibiotic distributions for trachoma. Clinical infectious diseases : an official publication of the Infectious Diseases Society of America. 2010 Sep;51(5):571–4.

11. Keenan JD, Sahlu I, McGee L, Cevallos V, Vidal JE, Chochua S, et al. Nasopharyngeal pneumococcal serotypes before and after mass azithromycin distributions for trachoma. Journal of the Pediatric Infectious Diseases Society. 2016 Jun;5(2):222–6.

12. Assefa A, Gelaw B, Shiferaw Y, Tigabu Z. Nasopharyngeal carriage and antimicrobial susceptibility pattern of streptococcus pneumoniae among pediatric outpatients at gondar university hospital, north west ethiopia. Pediatrics and neonatology. 2013 Oct;54(5):315–21.

13. Sime WT, Aseffa A, Woldeamanuel Y, Brovall S, Morfeldt E, Henriques-Normark B. Serotype and molecular diversity of nasopharyngeal Streptococcus pneumoniae isolates from children before and after vaccination with the ten-valent pneumococcal conjugate vaccine (PCV10) in Ethiopia. BMC infectious diseases. 2019 May;19(1):409.

14. Gebre T, Tadesse M, Aragaw D, Feye D, Beyene HB, Seyoum D, et al. Nasopharyngeal carriage and antimicrobial susceptibility patterns of streptococcus pneumoniae among children under five in southwest ethiopia. Children (Basel, Switzerland). 2017 Apr;4(4).

15. Wada FW, Tufa EG, Berheto TM, Solomon FB. Nasopharyngeal carriage of Streptococcus pneumoniae and antimicrobial susceptibility pattern among school children in South Ethiopia: post-vaccination era. BMC research notes. 2019 May;12(1):306.

16. Mulu W, Yizengaw E, Alemu M, Mekonnen D, Hailu D, Ketemaw K, et al. Pharyngeal colonization and drug resistance profiles of morraxella catarrrhalis, streptococcus pneumoniae, staphylococcus aureus, and haemophilus influenzae among HIV infected children attending ART clinic of felegehiwot referral hospital, ethiopia. PloS one. 2018;13(5):e0196722.

17. Negash AA, Asrat D, Abebe W, Hailemariam T, Gebre M, Verhaegen J, et al. Pneumococcal carriage, serotype distribution, and risk factors in children with community-acquired pneumonia, 5 years after introduction of the 10-valent pneumococcal conjugate vaccine in ethiopia. Open forum infectious diseases. 2019 Jun;6(6):ofz259.

18. Abaye G, Fekadu H, Haji K, Alemu D, Anjulo AA, Yadate DT. Prevalence and risk factors of pneumococcal nasopharyngeal carriage in healthy children attending kindergarten, in district of Arsi Zone, South East, Ethiopia. BMC research notes. 2019 May;12(1):253.

19. Abateneh DD, Shano AK, Dedo TW. Nasopharyngeal carriage of streptococcus pneumoniae and associated factors among children in Southwest Ethiopia. Open Microbiology Journal. 2020;14(1):171–8.

20. Haile AA, Gidebo DD, Ali MM. Colonization rate of Streptococcus pneumoniae, its associated factors and antimicrobial susceptibility pattern among children attending kindergarten school in Hawassa, southern Ethiopia. BMC research notes. 2019 Jun;12(1):344.

21. Seid M, Beyene G, Alemu Y, Workalemahu B, Delbo M, Taddesse D, et al. Does cotrimoxazole prophylaxis in HIV patients increase the drug resistance of pneumococci? A comparative cross-sectional study in southern Ethiopia. PloS one. 2020;15(12):e0243054.

22. Hussen S, Asnake S, Wachamo D, Tadesse BT. Pneumococcal nasopharyngeal carriage and antimicrobial susceptibility profile in children under five in southern Ethiopia. F1000Research. 2020;9:1466.

23. Lemma M, Bekele Y, Petkov S, Hägglund M, Petros B, Aseffa A, et al. Streptococcus pneumoniae nasopharyngeal carriage among PCV-10-vaccinated HIV-1-infected children with maintained serological memory in ethiopia. Pathogens (Basel, Switzerland). 2020 Feb;9(3).

24. Scott JAG, Mlacha Z, Nyiro J, Njenga S, Lewa P, Obiero J, et al. Diagnosis of invasive pneumococcal disease among children in Kenya with enzyme-linked immunosorbent assay for immunoglobulin G antibodies to pneumococcal surface adhesin A. Clinical and diagnostic laboratory immunology. 2005 Oct;12(10):1195–201.

25. Hamel MJ, Greene C, Chiller T, Ouma P, Polyak C, Otieno K, et al. Does cotrimoxazole prophylaxis for the prevention of HIV-associated opportunistic infections select for resistant pathogens in Kenyan adults? The American journal of tropical medicine and hygiene. 2008 Sep;79(3):320–30.

26. Nyandiko WM, Greenberg D, Shany E, Yiannoutsos CT, Musick B, Mwangi AW. Nasopharyngeal streptococcus pneumoniae among under-five year old children at the moi teaching and referral hospital, eldoret, kenya. East African medical journal. 2007 Apr;84(4):156–62.

27. Abdullahi O, Karani A, Tigoi CC, Mugo D, Kungu S, Wanjiru E, et al. The prevalence and risk factors for pneumococcal colonization of the nasopharynx among children in Kilifi District, Kenya. PloS one. 2012;7(2):e30787.

28. Tigoi CC, Gatakaa H, Karani A, Mugo D, Kungu S, Wanjiru E, et al. Rates of acquisition of pneumococcal colonization and transmission probabilities, by serotype, among newborn infants in Kilifi District, Kenya. Clinical infectious diseases : an official publication of the Infectious Diseases Society of America. 2012 Jul;55(2):180–8.

29. Conklin LM, Bigogo G, Jagero G, Hampton L, Junghae M, da Gloria Carvalho M, et al. High Streptococcus pneumoniae colonization prevalence among HIV-infected Kenyan parents in the year before pneumococcal conjugate vaccine introduction. BMC infectious diseases. 2016 Jan;16:18.

30. da Gloria Carvalho M, Pimenta FC, Moura I, Roundtree A, Gertz Jr. RE, Li Z, et al. Non-pneumococcal mitis-group streptococci confound detection of pneumococcal capsular serotype-specific loci in upper respiratory tract. PeerJ. 2013;1(1):e97.

31. Kobayashi M, Conklin LM, Bigogo G, Jagero G, Hampton L, Fleming-Dutra KE, et al. Pneumococcal carriage and antibiotic susceptibility patterns from two cross-sectional colonization surveys among children aged ¡5 years prior to the introduction of 10-valent pneumococcal conjugate vaccine - Kenya, 2009-2010. BMC infectious diseases. 2017 Jan;17(1):25.

32. Kobayashi M, Bigogo G, Kim L, Mogeni OD, Conklin LM, Odoyo A, et al. Impact of 10-valent pneumococcal conjugate vaccine introduction on pneumococcal carriage and antibiotic susceptibility patterns among children aged ¡5 years and adults with human immunodeficiency virus infection: Kenya, 2009-2013. Clinical infectious diseases : an official publication of the Infectious Diseases Society of America. 2020 Feb;70(5):814–26.

33. Hammitt LL, Ojal J, Bashraheil M, Morpeth SC, Karani A, Habib A, et al. Immunogenicity, impact on carriage and reactogenicity of 10-valent pneumococcal non-typeable Haemophilus influenzae protein D conjugate vaccine in Kenyan children aged 1-4 years: a randomized controlled trial. PloS one. 2014;9(1):e85459.

34. Hammitt LL, Akech DO, Morpeth SC, Karani A, Kihuha N, Nyongesa S, et al. Population effect of 10-valent pneumococcal conjugate vaccine on nasopharyngeal carriage of Streptococcus pneumoniae and non-typeable Haemophilus influenzae in Kilifi, Kenya: findings from cross-sectional carriage studies. The Lancet Global health. 2014 Jul;2(7):e397-405.

35. Feazel LM, Santorico SA, Robertson CE, Bashraheil M, Scott JAG, Frank DN, et al. Effects of vaccination with 10-valent pneumococcal non-typeable haemophilus influenza protein D conjugate vaccine (PHiD-CV) on the nasopharyngeal microbiome of kenyan toddlers. PloS one. 2015;10(6):e0128064.

36. Githii S, Revathi G, Muigai A, Kariuki S. Carriage rate and serotypes of Streptococcus pneumoniae amongst children in Thika Hospital, Kenya. African journal of laboratory medicine. 2013;2(1):45.

37. Hammitt LL, Etyang AO, Morpeth SC, Ojal J, Mutuku A, Mturi N, et al. Effect of ten-valent pneumococcal conjugate vaccine on invasive pneumococcal disease and nasopharyngeal carriage in Kenya: a longitudinal surveillance study. Lancet (London, England). 2019 May;393(10186):2146–54.

38. Farrar JL, Odiembo H, Odoyo A, Bigogo G, Kim L, Lessa FC, et al. Limited added value of oropharyngeal swabs for detecting pneumococcal carriage in adults. Open forum infectious diseases. 2020 Sep;7(9):ofaa368.

39. Heath CJ, Nayakwadi-Singer M, King CH, Malhotra I, Mutuku F, Mukoko D, et al. Nasopharyngeal carriage of streptococcus pneumoniae in children in coastal kenya. The American journal of tropical medicine and hygiene. 2018 Apr;98(4):1046–50.

40. Walekhwa M, Muturi M, Gunturu R, Kenya E, Kabera B, Walekhwa M. Streptococcus pneumoniae serotype epidemiology among PCV-10 vaccinated and unvaccinated children at gertrude’s children’s hospital, nairobi county: a cross-sectional study. F1000Research. 2019;7:879.

41. Dananché C, Paranhos-Baccalà G, Messaoudi M, Sylla M, Awasthi S, Bavdekar A, et al. Serotypes of streptococcus pneumoniae in children aged ¡5 years hospitalized with or without pneumonia in developing and emerging countries: a descriptive, multicenter study. Clinical infectious diseases : an official publication of the Infectious Diseases Society of America. 2020 Feb;70(5):875–83.

42. Bénet T, Sylla M, Messaoudi M, Picot VS, Telles JN, Diakite AA, et al. Etiology and factors associated with pneumonia in children under 5 years of age in Mali: A prospective case-control study. PLoS ONE [Internet]. 2015;10(12). Available from: https://www.embase.com/search/results?subaction=viewrecord&id=L608045097&from=export http://dx.doi.org/10.1371/journal.pone.0145447

43. Vallès X, Flannery B, Roca A, Mandomando I, Sigaúque B, Sanz S, et al. Serotype distribution and antibiotic susceptibility of invasive and nasopharyngeal isolates of Streptococcus pneumoniae among children in rural Mozambique. Tropical medicine & international health : TM & IH. 2006 Mar;11(3):358–66.

44. Brotons P, Bassat Q, Lanaspa M, Henares D, Perez-Arguello A, Madrid L, et al. Nasopharyngeal bacterial load as a marker for rapid and easy diagnosis of invasive pneumococcal disease in children from Mozambique. PloS one. 2017;12(9):e0184762.

45. Heinsbroek E, Tafatatha T, Chisambo C, Phiri A, Mwiba O, Ngwira B, et al. Pneumococcal acquisition among infants exposed to HIV in rural malawi: a longitudinal household study. American journal of epidemiology. 2016 Jan;183(1):70–8.

46. Glennie SJ, Banda D, Gould K, Hinds J, Kamngona A, Everett DDB, et al. Defective Pneumococcal-Specific Th1 Responses in HIV-Infected Adults Precedes a Loss of Control of Pneumococcal Colonization. Clin Infect Dis. 2013 Jan 15;56(2):291–9.

47. Massora S, Lessa FC, Moiane B, Pimenta FC, Mucavele H, Chaúque A, et al. Invasive disease potential of Streptococcus pneumoniae serotypes before and after 10-valent pneumococcal conjugate vaccine introduction in a rural area, southern Mozambique. Vaccine. 2019 Dec;37(51):7470–7.

48. Sigaúque B, Moiane B, Massora S, Pimenta F, Verani JR, Mucavele H, et al. Early declines in vaccine type pneumococcal carriage in children less than 5 years old after introduction of 10-valent pneumococcal conjugate vaccine in mozambique. The Pediatric infectious disease journal. 2018 Oct;37(10):1054–60.

49. Valenciano SJ, Moiane B, Lessa FC, Chaúque A, Massora S, Pimenta FC, et al. Effect of 10-valent pneumococcal conjugate vaccine on streptococcus pneumoniae nasopharyngeal carriage among children less than 5 years old: 3 years post-10-valent pneumococcal conjugate vaccine introduction in mozambique. Journal of the Pediatric Infectious Diseases Society. 2021 Apr;10(4):448–56.

50. Verani JR, Massora S, Acácio S, Dos Santos RT, Vubil D, Pimenta F, et al. Nasopharyngeal carriage of Streptococcus pneumoniae among HIV-infected and -uninfected children ¡5 years of age before introduction of pneumococcal conjugate vaccine in Mozambique. PloS one. 2018;13(2):e0191113.

51. Heinsbroek E, Tafatatha T, Phiri A, Swarthout TD, Alaerts M, Crampin AC, et al. Pneumococcal carriage in households in Karonga District, Malawi, before and after introduction of 13-valent pneumococcal conjugate vaccination. Vaccine. 2018 Nov;36(48):7369–76.

52. Adebanjo T, Lessa FC, Mucavele H, Moiane B, Chauque A, Pimenta F, et al. Pneumococcal carriage and serotype distribution among children with and without pneumonia in Mozambique, 2014-2016. PloS one. 2018;13(6):e0199363.

53. Pimenta FC, Moiane B, Lessa FC, Venero AKL, Moura I, Larson S, et al. Dried blood spots for Streptococcus pneumoniae and Haemophilus influenzae detection and serotyping among children ¡ 5 years old in rural Mozambique. BMC pediatrics. 2020 Jul;20(1):326.

54. Swarthout TD, Fronterre C, Lourenço J, Obolski U, Gori A, Bar-Zeev N, et al. High residual carriage of vaccine-serotype Streptococcus pneumoniae after introduction of pneumococcal conjugate vaccine in Malawi. Nature communications. 2020 May;11(1):2222.

55. Abotsi RE, Nicol MP, McHugh G, Simms V, Rehman AM, Barthus C, et al. Prevalence and antimicrobial resistance profiles of respiratory microbial flora in African children with HIV-associated chronic lung disease. BMC infectious diseases. 2021 Feb;21(1):216.

56. Ginsburg AS, Vitorino P, Qasim Z, Lenahan JL, Hwang J, Lamorte A, et al. Lung ultrasound patterns in paediatric pneumonia in mozambique and pakistan. ERJ Open Research. 2021;7(1):1–10.

57. Saeed KB, Jefferies JM, Wright SK, Lowdon SL, Clarke SC, Dryden MS. Carriage rates; circulating serotypes and antibiotic resistance among streptococcus pneumoniae in healthy infants in yei; south sudan. South Sudan med j. 2011;4(3):57–60.

58. Batt SL, Charalambous BM, Solomon AW, Knirsch C, Massae PA, Safari S, et al. Impact of azithromycin administration for trachoma control on the carriage of antibiotic-resistant Streptococcus pneumoniae. Antimicrobial agents and chemotherapy. 2003 Sep;47(9):2765–9.

59. Leung MHY, Oriyo NM, Gillespie SH, Charalambous BM. The adaptive potential during nasopharyngeal colonisation of Streptococcus pneumoniae. Infection, Genetics and Evolution. 2011;11(8):1989–95.

60. Chochua S, D’Acremont V, Hanke C, Alfa D, Shak J, Kilowoko M, et al. Increased nasopharyngeal density and concurrent carriage of streptococcus pneumoniae, haemophilus influenzae, and moraxella catarrhalis are associated with pneumonia in febrile children. PloS one. 2016;11(12):e0167725.

61. Moyo SJ, Steinbakk M, Aboud S, Mkopi N, Kasubi M, Blomberg B, et al. Penicillin resistance and serotype distribution of Streptococcus pneumoniae in nasopharyngeal carrier children under 5 years of age in Dar es Salaam, Tanzania. Journal of medical microbiology. 2012 Jul;61(Pt 7):952–9.

62. Ndossa A, Okamo B, Mushi MF, Mirambo MM, Kidenya BR, Hokororo A, et al. Factors associated with colonization of streptococcus pneumoniae among under-fives attending clinic in mwanza city, tanzania. Tanzania Journal of Health Research [Internet]. 2015;17(1). Available from: https://www.embase.com/search/results?subaction=viewrecord&id=L601473834&from=export http://dx.doi.org/10.4314/thrb.v17i1.1

63. Emgård M, Msuya SE, Nyombi BM, Mosha D, Gonzales-Siles L, Nordén R, et al. Carriage of penicillin-non-susceptible pneumococci among children in northern Tanzania in the 13-valent pneumococcal vaccine era. International journal of infectious diseases : IJID : official publication of the International Society for Infectious Diseases. 2019 Apr;81:156–66.

64. Bloch EM, West SK, Mabula K, Weaver J, Mrango Z, Munoz B, et al. Antibiotic resistance in young children in kilosa district, tanzania 4 years after mass distribution of azithromycin for trachoma control. The American journal of tropical medicine and hygiene. 2017 Sep;97(3):815–8.

65. Hercik C, Cosmas L, Mogeni OD, Wamola N, Kohi W, Omballa V, et al. A diagnostic and epidemiologic investigation of acute febrile illness (AFI) in Kilombero, Tanzania. PloS one. 2017;12(12):e0189712.

66. Bloch EM, Coles CL, Kasubi M, Weaver J, Mrango Z, Munoz B, et al. Biannual treatment of preschool children with single dose azithromycin to reduce mortality: Impact on azithromycin resistance in the MORDOR trial in tanzania. The American journal of tropical medicine and hygiene. 2020 Sep;103(3):1301–7.

67. Ngocho JS, Minja L, van der Gaast – de Jongh CE, Rahamat-Langendoen JC, Langereis JD, Mmbaga BT, et al. Viral-bacterial (co-)occurrence in the upper airways and the risk of childhood pneumonia in resource-limited settings. Journal of Infection. 2020;81(2):213–20.

68. Manyahi J, Moyo S, Aboud S, Langeland N, Blomberg B. High rate of antimicrobial resistance and multiple mutations in the dihydrofolate reductase gene among Streptococcus pneumoniae isolated from HIV-infected adults in a community setting in Tanzania. Journal of global antimicrobial resistance. 2020 Sep;22:749–53.

69. Kateete DP, Kajumbula H, Kaddu-Mulindwa DH, Ssevviri AK. Nasopharyngeal carriage rate of Streptococcus pneumoniae in Ugandan children with sickle cell disease. BMC research notes. 2012 Jan;5:28.

70. Blossom DB, Namayanja-Kaye G, Nankya-Mutyoba J, Mukasa JB, Bakka H, Rwambuya S, et al. Oropharyngeal colonization by Streptococcus pneumoniae among HIV-infected adults in Uganda: assessing prevalence and antimicrobial susceptibility. International journal of infectious diseases : IJID : official publication of the International Society for Infectious Diseases. 2006 Nov;10(6):458–64.

71. Lindstrand A, Kalyango J, Alfvén T, Darenberg J, Kadobera D, Bwanga F, et al. Pneumococcal carriage in children under five years in uganda-will present pneumococcal conjugate vaccines be appropriate? PloS one. 2016;11(11):e0166018.

72. Nackers F, Cohuet S, le Polain de Waroux O, Langendorf C, Nyehangane D, Ndazima D, et al. Carriage prevalence and serotype distribution of Streptococcus pneumoniae prior to 10-valent pneumococcal vaccine introduction: A population-based cross-sectional study in South Western Uganda, 2014. Vaccine. 2017 Sep;35(39):5271–7.

73. Rutebemberwa E, Mpeka B, Pariyo G, Peterson S, Mworozi E, Bwanga F, et al. High prevalence of antibiotic resistance in nasopharyngeal bacterial isolates from healthy children in rural Uganda: A cross-sectional study. Upsala journal of medical sciences. 2015;120(4):249–56.

74. Camelo IY, Mwananyanda LM, Thea DM, Seidenberg P, Gill CJ, Weinstein JR. A tale of 2 pneumos: The impact of human immunodeficiency virus exposure or infection status on pediatric nasopharyngeal carriage of streptococcus pneumoniae and pneumocystis jiroveci: a nested case control analysis from the pneumonia etiology research in. Clinical Infectious Diseases. 2021 Mar;72(6):1033–41.

75. Ndip RN, Ntiege EA, Ndip LM, Nkwelang G, Akoachere JFTK, Akenji T N. Antimicrobial resistance of bacterial agents of the upper respiratory tract of school children in Buea, Cameroon. Journal of health, population, and nutrition. 2008 Dec;26(4):397–404.

76. Njuma Libwea J, Gröndahl-Yli-Hannuksela K, Kobela M, Toropainen M, Nyholm O, Ndombo PK, et al. Prevalence of pneumococcal nasopharyngeal colonization and serotypes circulating in Cameroonian children after the 13-valent pneumococcal conjugate vaccine introduction. International journal of infectious diseases : IJID : official publication of the International Society for Infectious Diseases. 2020 Sep;98:113–20.

77. Birindwa AM, Emgård M, Nordén R, Samuelsson E, Geravandi S, Gonzales-Siles L, et al. High rate of antibiotic resistance among pneumococci carried by healthy children in the eastern part of the Democratic Republic of the Congo. BMC pediatrics. 2018 Nov;18(1):361.

78. Birindwa AM, Gonzales-Siles L, Nordén R, Geravandi S, Manegabe JT, Morisho L, et al. High bacterial and viral load in the upper respiratory tract of children in the Democratic Republic of the Congo. PloS one. 2020;15(10):e0240922.

79. Uddén F, Filipe M, Slotved HC, Yamba-Yamba L, Fuursted K, Pintar Kuatoko P, et al. Pneumococcal carriage among children aged 4 - 12 years in Angola 4 years after the introduction of a pneumococcal conjugate vaccine. Vaccine. 2020 Nov;38(50):7928–37.

80. Bere LC, Simpore J, Karou SD, Zeba B, Bere AP, Bannerman E, et al. Antimicrobial resistance and serotype distribution of Streptococcus pneumoniae strains causing childhood infection in Burkina Faso. Pakistan journal of biological sciences : PJBS. 2009 Sep;12(18):1282–6.

81. Hema-Ouangraoua S, Aziz Maiga A, Cairns M, Zongo I, Frédéric N, Serge Yerbanga R, et al. Impact of the addition of azithromycin to antimalarials used for seasonal malaria chemoprevention on antimicrobial resistance of Streptococcus pneumoniae. Tropical medicine & international health : TM & IH. 2019 Dec;24(12):1442–54.

82. Bonko MDA, Lompo P, Tahita MC, Kiemde F, Karama I, Somé AM, et al. Antibiotic susceptibility of staphylococcus aureus and streptococcus pneumoniae isolates from the nasopharynx of febrile children under 5 years in nanoro, burkina faso. Antibiotics (Basel, Switzerland). 2021 Apr;10(4).

83. Kiemde F, Tahita MC, Lompo P, Rouamba T, Some AM, Tinto H, et al. Treatable causes of fever among children under five years in a seasonal malaria transmission area in Burkina Faso. Infectious Diseases of Poverty [Internet]. 2018;7(1). Available from: https://www.embase.com/search/results?subaction=viewrecord&id=L622506406&from=export http://dx.doi.org/10.1186/s40249-018-0442-3

84. Bountogo M, Sanogo B, Pride MW, Jiang Q, Nikièma Z, Njanpop-Lafourcade BM, et al. Application of a pneumococcal serotype-specific urinary antigen detection test for identification of pediatric pneumonia in burkina faso. Pediatric Infectious Disease Journal. 2021;418–25.

85. Coulibaly B, Sié A, Kiemde D, Dembélé N, Compaore A, Dabo O, et al. Pneumococcal carriage and antibiotic resistance in children younger than 5 years in nouna district, burkina faso. The American journal of tropical medicine and hygiene. 2020 Aug;103(2):684–8.

86. Adiku TK, Asmah RH, Rodrigues O, Goka B, Obodai E, Adjei AA, et al. Aetiology of acute lower respiratory infections among children under five years in accra, ghana. Pathogens (Basel, Switzerland). 2015 Jan;4(1):22–33.

87. Donkor ES, Newman MJ, Oliver-Commey J, Bannerman E, Dayie NTKD, Badoe EV. Invasive disease and paediatric carriage of Streptococcus pneumoniae in Ghana. Scandinavian journal of infectious diseases. 2010 Apr;42(4):254–9.

88. Mills RO, Twum-Danso K, Owusu-Agyei S, Donkor ES. Epidemiology of pneumococcal carriage in children under five years of age in Accra, Ghana. Infectious diseases (London, England). 2015 May;47(5):326–31.

89. Dayie NTKD, Arhin RE, Newman MJ, Dalsgaard A, Bisgaard M, Frimodt-Møller N, et al. Penicillin resistance and serotype distribution of Streptococcus pneumoniae in Ghanaian children less than six years of age. BMC infectious diseases. 2013 Oct;13:490.

90. Dayie NTKD, Tetteh-Ocloo G, Labi AK, Olayemi E, Slotved HC, Lartey M, et al. Pneumococcal carriage among sickle cell disease patients in Accra, Ghana: Risk factors, serotypes and antibiotic resistance. PLoS One. 2018;13(11):e0206728.

91. Dayie NTKD, Tettey EY, Newman MJ, Bannerman E, Donkor ES, Labi AK, et al. Pneumococcal carriage among children under five in Accra, Ghana, five years after the introduction of pneumococcal conjugate vaccine. BMC pediatrics. 2019 Sep;19(1):316.

92. Dayie NT, Baffuor-Asare M, Labi AK, Obeng-Nkrumah N, Olayemi E, Lartey M, et al. Epidemiology of pneumococcal carriage among HIV-infected individuals in the conjugate vaccine era: a study in southern ghana. BioMed research international. 2019;2019:3427174.

93. Mills RO, Abdullah MR, Akwetey SA, Sappor DC, Cole I, Baffuor-Asare M, et al. Post-vaccination streptococcus pneumoniae carriage and virulence gene distribution among children less than five years of age, cape coast, ghana. Microorganisms. 2020 Dec;8(12).

94. Narwortey DK, Owusu-Ofori A, Slotved HC, Donkor ES, Ansah PO, Welaga P, et al. Nasopharyngeal carriage of Streptococcus pneumoniae among healthy children in Kassena-Nankana districts of Northern Ghana. BMC infectious diseases. 2021 Jul;21(1):661.

95. Hansen NS, Byberg S, Hervig Jacobsen L, Bjerregaard-Andersen M, Jensen AKG, Martins C, et al. Effect of early measles vaccine on pneumococcal colonization: A randomized trial from Guinea-Bissau. PloS one. 2017;12(5):e0177547.

96. Bénet T, Sánchez Picot V, Messaoudi M, Chou M, Eap T, Wang J, et al. Microorganisms associated with pneumonia in children ¡5 years of age in developing and emerging countries: The GABRIEL pneumonia multicenter, prospective, case-control study. Clinical infectious diseases : an official publication of the Infectious Diseases Society of America. 2017 Aug;65(4):604–12.

97. Ousmane S, Diallo BA, Ouedraogo R, Sanda AKA, Soussou AM, Collard JM. Serotype distribution and antimicrobial sensitivity profile of streptococcus pneumoniae carried in healthy toddlers before PCV13 introduction in niamey, niger. PloS one. 2017;12(1):e0169547.

98. Lagare A, Ousmane S, Dano ID, Issaka B, Issa I, Mainassara HB, et al. Molecular detection of respiratory pathogens among children aged younger than 5 years hospitalized with febrile acute respiratory infections: A prospective hospital-based observational study in Niamey, Niger. Health science reports. 2019 Oct;2(11):e137.

99. Dano ID, Ousmane S, Moumouni K, Lagare A, Issa I, Testa J. Risk factors associated with Streptococcus pneumonia carriage in children under five years old with acute respiratory infection in Niger. The Pan African medical journal. 2019;33:239.

100. Ibrahim DD, Ousmane S, Moumouni K, Mahamane AE. Measurement of pneumococcal IgG antibody, carriage and infection with Streptococcus pneumoniae in children under five years of age with acute respiratory infection in Niger. Journal of infection and public health. 2017;10(5):657–60.

101. Nwachukwu NC, Orji A. Streptococcus pneumoniae carriage rates among infants in Owerri, Nigeria. Afr j respir Med. 2008;4(1):16.

102. Nweze EI, Ezute S, Emeka NCC, Ogbonna CC, Eze C. Bacteria etiological agents causing respiratory tract infections in children and their resistance patterns to a panel of ten antibiotics. Asian Pacific Journal of Tropical Disease. 2012;2(1):18–23.

103. Adetifa IMO, Adamu AL, Karani A, Waithaka M, Odeyemi KA, Okoromah CAN, et al. Nasopharyngeal pneumococcal carriage in nigeria: a two-site, population-based survey. Scientific reports. 2018 Feb;8(1):3509.

104. Echave P, Bille J, Audet C, Talla I, Vaudaux B, Gehri M. Percentage, bacterial etiology and antibiotic susceptibility of acute respiratory infection and pneumonia among children in rural Senegal. Journal of tropical pediatrics. 2003 Feb;49(1):28–32.

105. Ba F, Seck A, Bâ M, Thiongane A, Cissé MF, Seck K, et al. Identifying an appropriate PCV for use in Senegal, recent insights concerning Streptococcus pneumoniae NP carriage and IPD in Dakar. BMC infectious diseases. 2014 Dec;14:627.

106. Tine RC, Ndiaye LA, Niang MN, Kiori DE, Dia N, Gaye O, et al. Upper respiratory infections in a rural area with reduced malaria transmission in Senegal: a pathogens community study. BMC infectious diseases. 2018 Sep;18(1):459.

107. Knobbe RB, Diallo A, Fall A, Gueye AD, Dieng A, van Immerzeel TD, et al. Pathogens causing respiratory tract infections in children less than 5 years of age in senegal. Microbiology insights. 2019;12:1178636119890885.

108. Hill PC, Akisanya A, Sankareh K, Cheung YB, Saaka M, Lahai G, et al. Nasopharyngeal carriage of Streptococcus pneumoniae in Gambian villagers. Clinical infectious diseases : an official publication of the Infectious Diseases Society of America. 2006 Sep;43(6):673–9.

109. Roca A, Hill PC, Townend J, Egere U, Antonio M, Bojang A, et al. Effects of community-wide vaccination with PCV-7 on pneumococcal nasopharyngeal carriage in the Gambia: a cluster-randomized trial. PLoS medicine. 2011 Oct;8(10):e1001107.

110. Usuf E, Badji H, Bojang A, Jarju S, Ikumapayi UN, Antonio M, et al. Pneumococcal carriage in rural Gambia prior to the introduction of pneumococcal conjugate vaccine: a population-based survey. Tropical medicine & international health : TM & IH. 2015 Jul;20(7):871–9.

111. Burr SE, Milne S, Jafali J, Bojang E, Rajasekhar M, Hart J, et al. Mass administration of azithromycin and Streptococcus pneumoniae carriage: cross-sectional surveys in the Gambia. Bulletin of the World Health Organization. 2014 Jul;92(7):490–8.

112. Roca A, Dione MM, Bojang A, Townend J, Egere U, Darboe O, et al. Nasopharyngeal carriage of pneumococci four years after community-wide vaccination with PCV-7 in The Gambia: long-term evaluation of a cluster randomized trial. PloS one. 2013;8(9):e72198.

113. Foster-Nyarko E, Kwambana B, Aderonke O, Ceesay F, Jarju S, Bojang A, et al. Associations between nasopharyngeal carriage of Group B Streptococcus and other respiratory pathogens during early infancy. BMC microbiology. 2016 May;16:97.

114. Odutola A, Ota MOC, Antonio M, Ogundare EO, Saidu Y, Foster-Nyarko E, et al. Efficacy of a novel, protein-based pneumococcal vaccine against nasopharyngeal carriage of Streptococcus pneumoniae in infants: A phase 2, randomized, controlled, observer-blind study. Vaccine. 2017 May;35(19):2531–42.

115. Usuf E, Bojang A, Camara B, Jagne I, Oluwalana C, Bottomley C, et al. Maternal pneumococcal nasopharyngeal carriage and risk factors for neonatal carriage after the introduction of pneumococcal conjugate vaccines in The Gambia. Clinical microbiology and infection : the official publication of the European Society of Clinical Microbiology and Infectious Diseases. 2018 Apr;24(4):389–95.

116. Bojang A, Camara B, Jagne Cox I, Oluwalana C, Lette K, Usuf E, et al. Long-term impact of oral azithromycin taken by gambian women during labor on prevalence and antibiotic susceptibility of streptococcus pneumoniae and staphylococcus aureus in their infants: Follow-up of a randomized clinical trial. Clinical infectious diseases : an official publication of the Infectious Diseases Society of America. 2018 Sep;67(8):1191–7.

117. Dunne EM, Hua Y, Salaudeen R, Hossain I, Ndiaye M, Ortika BD, et al. Insights into pneumococcal pneumonia using lung aspirates and nasopharyngeal swabs collected from pneumonia patients in The Gambia. The Journal of infectious diseases. 2020 Apr;

118. Usuf E, Bottomley C, Bojang E, Cox I, Bojang A, Gladstone R, et al. Persistence of nasopharyngeal pneumococcal vaccine serotypes and increase of nonvaccine serotypes among vaccinated infants and their mothers 5 years after introduction of pneumococcal conjugate vaccine 13 in the gambia. Clinical infectious diseases : an official publication of the Infectious Diseases Society of America. 2019 Apr;68(9):1512–21.

119. Kelly MS, Surette MG, Smieja M, Rossi L, Luinstra K, Steenhoff AP, et al. Pneumococcal colonization and the nasopharyngeal microbiota of children in botswana. The Pediatric infectious disease journal. 2018 Nov;37(11):1176–83.

120. McNally LM, Jeena PM, Gajee K, Sturm AW, Tomkins AM, Coovadia HM, et al. Lack of association between the nasopharyngeal carriage of Streptococcus pneumoniae and Staphylococcus aureus in HIV-1-infected South African children. The Journal of infectious diseases. 2006 Aug;194(3):385–90.

121. Pemba L, Charalambous S, von Gottberg A, Magadla B, Moloi V, Seabi O, et al. Impact of cotrimoxazole on non-susceptibility to antibiotics in Streptococcus pneumoniae carriage isolates among HIV-infected mineworkers in South Africa. The Journal of infection. 2008 Mar;56(3):171–8.

122. Ditse Z, Adrian PV, Kuwanda L, Madhi SA. Association of Streptococcus pneumoniae common protein antigen (CPA) antibodies and pneumococcal nasopharyngeal colonization in HIV-infected and HIV-uninfected African children. Vaccine. 2013 Sep;31(40):4421–7.

123. Madhi SA, Izu A, Nunes MC, Violari A, Cotton MF, Jean-Philippe P, et al. Longitudinal study on Streptococcus pneumoniae, Haemophilus influenzae and Staphylococcus aureus nasopharyngeal colonization in HIV-infected and -uninfected infants vaccinated with pneumococcal conjugate vaccine. Vaccine. 2015 May;33(23):2662–9.

124. Olwagen CP, Adrian PV, Madhi SA. Evaluation of the impact of HIV-1 infection and density of common nasopharyngeal bacterial colonizers in South African children immunized with 7-valent pneumococcal conjugate vaccine. Vaccine. 2020 Feb;38(7):1762–9.

125. Albrich WC, Madhi SA, Adrian PV, Telles JN, Paranhos-Baccalà G, Klugman KP. Genomic load from sputum samples and nasopharyngeal swabs for diagnosis of pneumococcal pneumonia in HIV-infected adults. Journal of clinical microbiology. 2014 Dec;52(12):4224–9.

126. Nunes MC, Jones SA, Groome MJ, Kuwanda L, Van Niekerk N, von Gottberg A, et al. Acquisition of Streptococcus pneumoniae in South African children vaccinated with 7-valent pneumococcal conjugate vaccine at 6, 14 and 40 weeks of age. Vaccine. 2015 Jan;33(5):628–34.

127. Madhi SA, Moreira M, Koen A, van Niekerk N, de Gouveia L, Jose L, et al. Impact of HIV status and vaccination schedule on bacterial nasopharyngeal carriage following infant immunisation with the pneumococcal non-typeable Haemophilus influenzae protein D conjugate vaccine in South Africa. Vaccine. 2020 Feb;38(10):2350–60.

128. Nzenze SA, Shiri T, Nunes MC, Klugman KP, Kahn K, Twine R, et al. Temporal changes in pneumococcal colonization in a rural African community with high HIV prevalence following routine infant pneumococcal immunization. The Pediatric infectious disease journal. 2013 Nov;32(11):1270–8.

129. Nzenze SA, von Gottberg A, Shiri T, van Niekerk N, de Gouveia L, Violari A, et al. Temporal changes in pneumococcal colonization in HIV-infected and HIV-uninfected mother-child pairs following transitioning from 7-valent to 13-valent pneumococcal conjugate vaccine, soweto, south africa. The Journal of infectious diseases. 2015 Oct;212(7):1082–92.

130. Wright MS, McCorrison J, Gomez AM, Beck E, Harkins D, Shankar J, et al. Strain level streptococcus colonization patterns during the first year of life. Frontiers in microbiology. 2017;8:1661.

131. Vanker A, Nduru PM, Barnett W, Dube FS, Sly PD, Gie RP, et al. Indoor air pollution and tobacco smoke exposure: impact on nasopharyngeal bacterial carriage in mothers and infants in an African birth cohort study. ERJ open research. 2019 Feb;5(1).

132. Madhi SA, Nzenze SA, Nunes MC, Chinyanganya L, Van Niekerk N, Kahn K, et al. Residual colonization by vaccine serotypes in rural South Africa four years following initiation of pneumococcal conjugate vaccine immunization. Expert review of vaccines. 2020 Apr;19(4):383–93.

133. Velaphi SC, Westercamp M, Moleleki M, Pondo T, Dangor Z, Wolter N, et al. Surveillance for incidence and etiology of early-onset neonatal sepsis in Soweto, South Africa. PLoS ONE [Internet]. 2019;14(4). Available from: https://www.embase.com/search/results?subaction=viewrecord&id=L2001810568&from=export http://dx.doi.org/10.1371/journal.pone.0214077

134. Skosana Z, Von Gottberg A, Olorunju S, Mohale T, Du Plessis M, Adams T, et al. Non-vaccine serotype pneumococcal carriage in healthy infants in South Africa following introduction of the 13-valent pneumococcal conjugate vaccine. South African medical journal = Suid-Afrikaanse tydskrif vir geneeskunde. 2021 Feb;111(2):143–8.
